# The Fossil Record of Elateridae (Coleoptera: Elateroidea): Described Species, Current Problems and Future Prospects

**DOI:** 10.3390/insects12040286

**Published:** 2021-03-25

**Authors:** Robin Kundrata, Gabriela Packova, Alexander S. Prosvirov, Johana Hoffmannova

**Affiliations:** 1 Department of Zoology, Faculty of Science, Palacky University, 17. listopadu 50, 77146 Olomouc, Czech Republic; gabriela.packova01@upol.cz (G.P.); johana.hoffmannova01@upol.cz (J.H.); 2 Department of Entomology, Faculty of Biology, Moscow State University, Leninskie Gory 1/12, 119234 Moscow, Russia; carrabus69@mail.ru

**Keywords:** catalogue, classification, Cenozoic, click-beetles, Eucnemidae, evolution, Mesozoic, palaeodiversity, systematics

## Abstract

**Simple Summary:**

Beetle fossils play an important role in our understanding of the origin and evolutionary history of this insect order. Despite the recently increasing rate of fossil research focused on the click-beetles (Coleoptera: Elateridae), the major group in the superfamily Elateroidea, their palaeodiversity has still remained largely understudied. In this study, we summarized current knowledge on the click-beetle fossil record with a main emphasis on the described diversity. We compiled an annotated catalogue of all described fossil species in Elateridae, assessed the reliability of their systematic placement, and discuss the current state of knowledge and prospects of research of the fossil record in the group. This study should serve as a comprehensive basis for all subsequent research dealing with the origin, early evolution and diversity of Elateridae.

**Abstract:**

The Elateridae (click-beetles) are the largest family in Elateroidea; however, their relationships, systematics and classification remain unclear. Our understanding of the origin, evolution, palaeodiversity and palaeobiogeography of Elateridae, as well as reconstruction of a reliable time-calibrated phylogeny for the group, are hampered by the lack of detailed knowledge of their fossil record. In this study, we summarize the current knowledge on all described fossil species in Elateridae, including their type material, geographic origin, age, bibliography and remarks on their systematic placement. Altogether, 261 fossil species classified in 99 genera and nine subfamilies are currently listed in this family. The Mesozoic click-beetle diversity includes 143 species, with most of them described from the Jurassic Karatau, and 118 described species are known from the Cenozoic deposits, mainly from the Eocene North American Florissant Formation and European Baltic amber. Available data on the described past diversity of Elateridae suggest that almost all fossil lineages in this group are in urgent need of revision and numerous Mesozoic species might belong to different families. Our study is intended to serve as a comprehensive basis for all subsequent research focused on the click-beetle fossil record.

## 1. Introduction

The click-beetles (Elateridae) are the major family in Elateroidea, comprising more than 10,000 described species worldwide [1]. Despite the efforts of numerous studies using morphological or molecular data, the classification and phylogenetic relationships within the family remain far from fully understood [2,3,4,5,6,7,8,9,10,11]. Taking this into consideration, further development of click-beetle systematics and understanding their evolution would certainly benefit from integrating modern molecular-based methods and morphology with fossils into a combined phylogenetic approach. While our knowledge on the systematics and diversity of recent click-beetle lineages has grown considerably in recent decades, their fossil record has been only scarcely investigated, lacking any comprehensive studies [12].

The first fossil elaterid species were reported and described during the mid-19th century from European localities [13,14,15,16]. Those were usually compression fossils ranging in their age from the Upper Triassic to Miocene. Later, several other species were added from Europe and the USA [17,18,19,20,21]. Scudder [22,23,24] summarized information on fossil Coleoptera of that time, including Elateridae. Lomnicki [25] described a new species from the Miocene of Ukraine. Handlirsch [26], in his monumental work “Die fossilen Insekten und die Phylogenie der rezenten Formen”, listed numerous fossil taxa in Elateridae, many of them of doubtful placement. Wickham [27,28] described the diversity of Elateridae in the Florissant deposit in Colorado, USA, and he later provided a checklist of all North American fossil beetles, including Elateridae [29]. Klebs [30] provided a checklist of elaterid genera found in Eocene North European Baltic amber. Cockerell published hundreds of papers on various taxa and fossil deposits, including several ones treating Elateridae from the Triassic of the United Kingdom [31], Burmese amber [32,33], the Eocene of the United Kingdom [34], the Paleocene of Argentina [35] or the Eocene of the USA [36]. Tillyard [37,38] and Dunstan [39] studied beetle fossils from Triassic deposits in Australia and attributed several taxa to Elateridae. Later authors continued in dealing with just a single or several species of Elateridae without any revision or a more comprehensive picture, including Ping [40] (a new monotypic genus from the Cretaceous of China), Wickham [41] (a single new species from the Eocene of the USA), Theobald [42] (a single species from the Oligocene of Germany), Piton [43] (two species from the Paleocene of France), Haupt [44] (a single species from the Eocene of Germany), Gardiner [45] (a new monotypic genus), and Becker [46] (one new genus and three new species from Miocene Mexican amber).

Iablokoff-Khnzorian [47] was the first author who provided a comprehensive revision of Elateridae in Baltic amber. In that study, he described seven genera, three subgenera and 11 species, which were also included in subsequent studies devoted to the Baltic amber [48,49]. Dolin [50] studied a Mesozoic fossil in Kyrgyzstan and described two monotypic genera which he classified in his newly established tribe Protagrypnini within Agrypninae. Later, he focused on the detailed examination of click-beetle fossils from the Jurassic deposit of Karatau, Kazakhstan. First, he elevated Protagrypnini to the subfamily level and defined three tribes in it, describing 15 species in three genera [51]. Later, he described eight species in four genera in the subfamilies Cardiophorinae and Negastriinae [52], and finally summarized the diversity of Elateridae as a whole from the Karatau deposit [53], with 107 species classified in 31 genera and five subfamilies known from that locality. Crowson [54] was the first who reported two undescribed elaterids from Cretaceous Lebanese amber. Particular attention was also paid to Chinese fossil deposits. Several click-beetles were reported from Jurassic [55,56], Cretaceous [57] and Miocene sediments [58,59]. Whalley [60] revised Jurassic insects of Dorset, England, including Elateridae, and Zaragoza-Caballero [61] descibed a new species from Mexican amber. Tröster [62,63,64,65,66,67] studied in detail Elateridae of the Eocene Grube Messel Pit and Eckfeldt Maar deposits in Germany, and focused mainly on the agrypnine genus *Macropunctum* Tröster, 1991. The comprehensive, illustrated catalogue “Treatise on Invertebrate Paleontology” by Carpenter [68] was an important contribution, in which he also included a list of fossil Elateridae, and placed many dubious taxa, especially those described by Handlirsch [26], as Coleoptera *incertae sedis*.

In the 21st century, Wappler [69] and Alekseev [70] described additional species of Eocene *Macropunctum* from Germany and the United Kingdom, respectively. Martins-Neto et al. [71] and Martins-Neto and Gallego [72] reported several putative elaterids from the Triassic fossil deposits in Argentina, Martin [73] described a new click-beetle species from the Jurassic of Australia, and Alekseev [74,75] and Sohn et al. [76] discovered new fossil click-beetles in the Cretaceous deposits in Russia and South Korea, respectively. In their revision of fossil Cerophytidae, Chang et al. [77] discovered that some taxa which Dolin [53] described in Elateridae are in fact cerophytids. However, the main attention regarding the fossil Elateridae in the 21st century was definitely paid to the Chinese Mesozoic deposits and inclusions in various ambers. Regarding the Elateridae fossils discovered in China, they belong either to Jurassic [78,79,80,81] or Cretaceous deposits [82,83,84,85,86] (for reviews, see [87,88,89]). Regarding click-beetles included in amber, their diversity in Miocene Mexican “Chiapas” amber, along with other insects, was summarized by Solórzano Kraemer [90]. Schimmel [91] described several new species of Megapenthini from North European Baltic amber. Alekseev [92] provided a checklist of beetles described from that amber, and later Kirejtshuk and Kovalev [93] added the first representative of Omalisidae (currently the elaterid subfamily Omalisinae [7]), and Kundrata et al. [94] described a new genus in the lissomine tribe Protelaterini. Kirejtshuk and Azar [95] reported several unnamed Elateridae from Cretaceous Lebanese amber. Otto [96] added a new genus from Cretaceous Burmese amber, which he classified in Pityobiinae.

A comprehensive online checklist of fossil beetles by Kirejtshuk and Ponomarenko [97] also covers Elateridae but requires some updates, including changes in classification. Oberprieler et al. [98] reported a possible elaterid fossil from the Upper Jurassic of Australia. Most recently, Kundrata et al. [12] compiled an updated comprehensive summary of the fossil genera in Elateridae, including their systematic placement and information on the type species, gender, number of species, age range, and relevant bibliography, and Muona et al. [99] revised the clicking Elateroidea from Chinese Mesozoic deposits, with several former click-beetle taxa transferred to Throscidae and Eucnemidae. This study is a follow up to the annotated catalogue of fossil genera in Elateridae [12]. Our understanding of the origin, evolution, palaeodiversity and palaeobiogeography of Elateridae, as well as the reconstruction of a reliable time-calibrated phylogeny for the group, are hampered by the lack of a detailed investigation of their known fossil record. Therefore, this study aims to summarize the current knowledge on all described fossil species in Elateridae, including their synonyms, misspellings, type material, geographic origin, age, bibliography and their systematic placement according to the most recent publications. It should serve as a comprehensive basis for all subsequent research dealing with a click-beetle fossil record, including the studies on the early evolution and diversity of Elateridae.

## 2. Materials and Methods

We compiled information on all fossil species in Elateridae. Inclusions in the Holocene copal *sensu* Solórzano Kraemer et al. [100] were not included in this study as they most probably represent recent species [101,102,103,104,105]. The higher classification of Elateridae follows Kundrata et al. [6,12]. The compositions of the subfamilies and tribes follow Johnson [106], Cate [107], Douglas [108], Kundrata et al. [12,94,109], and citations therein. We follow the style used in the first part of the World catalogue of the genus-group names in extant Elateridae [109] and the catalogue of the genus-group names in fossil Elateridae [12]. The names of the family, genus- and species-group taxa are given with the name of the author, and the year and page of publication. The page given is the page where the taxon name and description are printed. The year and page given for the incorrect subsequent spellings are the first year and page in which they are used. The detailed information for family group names is given in Bouchard et al. [110] and that for genus-group names (including synonyms, misspellings, unavailable names, type species and their designations) can be found in various publications and catalogues [12,107,109,111,112] and is not repeated here. Only generic misspellings and unavailable names which were not included in the catalogue of fossil genera [12] are reported here, and these are given under genus-names only and are not repeated under species-names. Misspellings and unavailable names are followed by colon “:”. For each fossil species in Elateridae, we provide all synonyms, information on the type series and type depositories, fossil deposit and age, and relevant bibliography. Information on the type depositories was taken from the original descriptions, the museum webpages, curators, or from the Paleobiology Database (https://paleobiodb.org/; accessed on 10 October 2020). Taxa marked with an asterisk (*) also contain recent species. The age of fossils was taken from the Paleobiology Database (https://paleobiodb.org/; accessed on 10 October 2020). Divisions of geological time and their boundaries follow the ICS International Chronostratigraphic Chart v. 2020/03 (http://www.stratigraphy.org/; accessed on 10 October 2020) [113]. We assessed each fossil species based on its original description and available illustrations to conclude whether its position in Elateridae and its generic attribution can be considered reliable or not. Most doubtful family or generic placements are discussed within the Remark section under the particular species. General information about species within the same genus are given in the Remark section under the particular genus. Proper taxonomic placement of listed fossil click-beetle lineages needs a detailed investigation of the type material which was far beyond the scope of this study, and hence the remarks on potential systematic misplacement of some taxa do not represent formal taxonomic decisions unless stated otherwise. An overview of the fossil Elateridae is summarized in Appendix A (Table A1).

### Abbreviations

Abbreviations for museums and collections:
BMNHNatural History Museum, London, The United KingdomCNUKey Laboratory of Insect Evolution and Environmental Changes, College of Life Science, Capital Normal University, Beijing, ChinaCUBUniversity of Colorado Museum of Natural History, Boulder, Colorado, USAETHSwiss Federal Institute of Technology, Zurich, SwitzerlandFISForschungsstation Grube Messel of the Senckenberg Forschungsinstitut und Naturmuseum, Frankfurt am Main, Germany (= SMF)GIHGeologisches Institut Halle (Saale), GermanyGNUEGongju National University of Education, Gongju, South KoreaGPIBOSteinmann Institute for Geology, Mineralogy and Palaeontology, University of Bonn, GermanyGPIUHGeological-Paleontological Museum of the University of Hamburg, GermanyGSCGeological Survey of Canada, Ottawa, CanadaHLMDHessisches Landesmuseum, Darmstadt, GermanyMCZMuseum of Comparative Zoology, Harvard University, Cambridge, Massachusetts, USAMNHNMuseum National d’Histoire Naturelle, Paris, FranceNHMBNaturhistorisches Museum, Basel, SwitzerlandNHMMNaturhistorisches Museum Mainz, Mainz, GermanyNIGPNanjing Institute of Geology and Palaeontology, Nanjing, ChinaPINPalaeontological Institute of the Russian Academy of Sciences, Moscow, RussiaQMQueensland Museum, Brisbane, AustraliaSGMSShandong Geological Museum, Jinan, Shandong, ChinaSMJSShandong Museum (= Shandong Provincial Museum), Jinan, Shandong, ChinaSMNHSwedish Museum of Natural History (Naturhistoriska Riksmuseet), Stockholm, SwedenSMNKStaatliches Museum für Naturkunde Karlsruhe, GermanySNAAStiftung Naturama Aargau, Aarau, SwitzerlandUCMPUniversity of California Museum of Paleontology, Berkeley, California, USAUNAMInstituto de Biología, Universidad Nacional Autónoma de México, MexicoUSNMSmithsonian Institution, National Museum of Natural History, Washington, D.C., USAUZHUniversity of Zurich, SwitzerlandWAMInvertebrate Paleontology collection, Western Australian Museum, AustraliaWIRCWisconsin Insect Research Collection, Department of Entomology at the University of Wisconsin, Madison, Wisconsin, USAYPMYale Peabody Museum of Natural History, New Haven, Connecticut, USAZINZoological Institute of Russian Academy of Sciences, St. Petersburg, Russia

## 3. Results

Family Elateridae Leach, 1815 *

Elaterides Leach, 1815: 85 [114]. Type genus: *Elater* Linnaeus, 1758: 404 [115]. For more information, including synonyms, see Bouchard et al. [110] and Kundrata et al. [109].

### 3.1. Subfamily Agrypninae Candèze, 1857 *

Agrypnides Candèze, 1857: 17 [116]. Type genus: *Agrypnus* Eschscholtz, 1829: 32 [117]. For more information, including synonyms, see Kundrata et al. [109].

#### 3.1.1. Tribe Agrypnini Candèze, 1857 *

Agrypnides Candèze, 1857: 17 [116]. Type genus: *Agrypnus* Eschscholtz, 1829: 32 [117]. For more information, including synonyms, see Kundrata et al. [109].

 

- Genus *Adelocera* Latreille, 1829 *

*Adelocera* Latreille, 1829: 451 [118]. Type species: *Elater ovalis* Germar, 1823: 49 [119] (ICZN application needed). For more information, including synonyms, see Kundrata et al. [109].

 

***Adelocera perantiqua* Cockerell and LeVeque, 1931**

*Adelocera perantiqua* Cockerell and LeVeque, 1931: 359 [36].

Type material. Holotype, sex unknown, compression fossil, No. 15,571 (CUB).

Fossil deposit/age. USA: Colorado, Green River Formation, Station 20, Roan Plateau; 50.3–46.2 Ma (Eocene).

Literature. Cockerell and LeVeque (1931: 359): original description [36].

Remark. According to the body proportions, shape of antenna and structure of thorax (e.g., pronotosternal sutures apparently closed, posterior angles of pronotum sharp and long, and prosternal process long, acute and with a tooth), this species does not belong neither to the currently defined genus *Adelocera* nor to the tribe Agrypnini. Based on the general habitus, long posterior angles of pronotum and marked elytra, it might be a member of Agrypninae: Oophorini. We prefer to postpone any taxonomic changes pending a comprehensive review including the type material.

 

- **Genus *Ageratus* Dolin, 1980**

*Ageratus* Dolin, 1980: 72 [53]. Type species: *Ageratus ponomarenkoi* Dolin, 1980: 73 [53]. For more information, see Kundrata et al. [12].

Remark. Type species of *Ageratus* is morphologically similar to the genera currently classified in Pseudomelanactini, and, therefore, the systematic position of this genus needs further examination.

 

***Ageratus delicatus* Dolin, 1980**

*Ageratus delicatus* Dolin, 1980: 73 [53].

Type material. Holotype, sex unknown, exoskeleton, compression fossil, No. 2452/87 (PIN).

Fossil deposit/age. Kazakhstan: Karabastau Formation, Karatau, Galkino; 166.1–157.3 Ma (Jurassic).

Literature. Dolin (1980: 73): original description [53]; Korneev and Cate (2005: 14): checklist [120].

Remark. This species differs from the type species of *Ageratus* in the body proportions and smaller body size, relatively shorter and broader prothorax, and the presence of long sublateral carinae on pronotum. Based on these characters, *A. delicatus* reminds representatives of genera *Agrypnus* Eschscholtz, 1829 or *Compsolacon* Reitter, 1905.

 

***Ageratus ponomarenkoi* Dolin, 1980**

*Ageratus ponomarenkoi* Dolin, 1980: 73 [53].

Type material. Holotype, sex unknown, exoskeleton, compression fossil, No. 1739/35 (part + counterpart) (PIN).

Fossil deposit/age. Kazakhstan: Karabastau Formation, Karatau, Galkino; 166.1–157.3 Ma (Jurassic).

Literature. Dolin (1980: 73): original description [53]; Carpenter (1992: 304): generic catalogue [68]; Korneev and Cate (2005: 9): checklist [120]; Kundrata et al. (2020: 5): generic catalogue [12].

Remark. This species is similar in the shape of antennae, the elongated prothorax with deep, fully excavated pronotosternal sutures, and the elongated elytra to genera *Lanelater* Arnett, 1952 and *Anthracalaus* Fairmaire, 1888 (Agrypninae: Pseudomelanactini). Its placement in Agrypnini should be re-evaluated.

 

- Genus *Agrypnus* Eschscholtz, 1829 *

*Agrypnus* Eschscholtz, 1829: 32 [117]. Type species: *Elater murinus* Linnaeus, 1758: 406 [115]. For more information, including synonyms, see Sánchez-Ruiz [121] and Kundrata et al. [109].

 

***Agrypnus exhumatus* (Wickham, 1916), comb. nov.**

*Lacon exhumatus* Wickham, 1916: 501 [28].

Type material. Holotype, sex unknown, exoskeleton, compression fossil, No. 2776, No. 4456 in Scudder coll. (MCZ).

Fossil deposit/age. USA: Colorado, Florissant Formation, Florissant; 37.2–33.9 Ma (Eocene).

Literature. Wickham (1916: 501): original description [28]; Wickham (1920: 354): catalogue [29].

Remark. This species was originally compared with *Lacon rectangularis* (Say, 1825) [28], which has been currently classified in *Agrypnus. Lacon exhumatus* Wickham, 1916 morphologically fits to the genus *Agrypnus sensu* Hayek [122].

 

- **Genus *Compsoderus* Dolin, 1980**

*Compsoderus* Dolin, 1980: 71 [53]. Type species: *Compsoderus priscus* Dolin, 1980: 72 [53]. For more information, see Kundrata et al. [12].

Remark. This genus might represent Eucnemidae based on the habitus and shape of prothorax; however, the type specimen should be examined to find supporting characters as defined by Muona et al. [99].

 

***Compsoderus priscus* Dolin, 1980**

*Compsoderus priscus* Dolin, 1980: 72 [53].

Type material. Holotype, sex unknown, exoskeleton, compression fossil, No. 2066/2975 (PIN).

Fossil deposit/age. Kazakhstan: Karabastau Formation, Karatau, Mikhailovka; 166.1–157.3 Ma (Jurassic).

Literature. Dolin (1980: 72): original description [53]; Carpenter (1992: 304): generic catalogue [68]; Korneev and Cate (2005: 10): checklist [120]; Kundrata et al. (2020: 5): generic catalogue [12].

 

- Genus *Lacon* Laporte, 1838 *

*Lacon* Laporte, 1838: 11 [123]. Type species: *Elater atomarius* Fabricius, 1798: 139 [124] (=*Elater punctatus* Herbst, 1784: 110 [125]). For more information, including synonyms, see Kundrata et al. [109].

 

***Lacon granulatus* (Heer, 1847), comb. nov.**

*Adelocera granulata* Heer, 1847: 139 [14].

Type material. Holotype, sex unknown, compression fossil (SNAA).

Fossil deposit/age. Germany: Upper Freshwater-Molasse Formation, Öhningen; 12.7–11.608 Ma (Miocene).

Literature. Heer (1847: 139): original description [14]; Giebel (1852: 651): catalogue [126]; Giebel (1856: 96): redescription [16]; Scudder (1891: 459): catalogue [24]; Handlirsch (1907: 743): catalogue [127]; Piton (1940: 179): remark [43].

Remark. Based on the generic diagnoses of *Adelocera* and *Lacon* by Hayek [122] (see also [111,128]), we transfer *A. granulata* to *Lacon*. However, this placement should be taken as tentative due to the absence of crucial diagnostic characters in the fossil.

 

***Lacon jungi* (Piton, 1940), comb. nov.**

*Adelocera jungi* Piton, 1940: 178 [43].

Type material. Holotype, sex unknown, exoskeleton, compression fossil, No. 76 (MNHN).

Fossil deposit/age. France: Menat Formation, Menat; 61.6–59.2 Ma (Paleocene).

Literature. Piton (1940: 178): original description [43].

Remark. Based on the general habitus, the length and shape of antenna, and the shape of pronotum, we transfer *A. jungi* to *Lacon*. For the generic diagnoses of *Adelocera* and *Lacon* see Hayek [122].

 

***Lacon primordialis* Heer, 1847**

*Lacon primordialis* Heer, 1847: 138 [14].

Type material. Holotype, sex unknown, compression fossil, No. 7887 (ETH).

Fossil deposit/age. Germany: Upper Freshwater-Molasse Formation, Öhningen; 12.7–11.608 Ma (Miocene).

Literature. Heer (1847: 138): original description [14]; Giebel (1852: 651): catalogue [126]; Giebel (1856: 96): redescription [16]; Scudder (1891: 544): catalogue [24]; Handlirsch (1907: 743): catalogue [127].

Remark. Based on the body proportions and the closed pronotosternal sutures, this species does not fit into the current diagnosis of neither the genus *Lacon* nor the tribe Agrypnini [122]. Superficially this species resembles Dendrometrinae *sensu lato* [6]. However, we prefer to postpone any taxonomic changes pending a comprehensive review including the type material.

 

- **Genus *Litholacon* Dolin, 1980**

*Litholacon* Dolin, 1980: 67 [53]. Type species: *Litholacon derumpens* Dolin, 1980: 68 [53]. For more information, see Kundrata et al. [12].

Remark. This genus is in a need of revision since some species strongly differ from the type species (and also from each other) in various diagnostic characters, e.g., the body proportions and shapes of antenna, prothorax and elytra. Several species, especially those with closed pronotosternal sutures, which is a character typical for Agrypnini, resemble representatives of Negastriinae or Dendrometrinae: Hypnoidini. However, many species of this genus, if not all, might be in fact Eucnemidae, which is suggested by their rather short and broad thorax (in some species with furrowed pronotosternal sutures), short and broad prosternal process, compact elytra, and the shape of antenna.

 

***Litholacon conicicollis* Dolin, 1980**

*Litholacon conicicollis* Dolin, 1980: 70 [53].

Type material. Holotype, sex unknown, exoskeleton, compression fossil, No. 2784/1371 (PIN).

Fossil deposit/age. Kazakhstan: Karabastau Formation, Karatau, Mikhailovka; 166.1–157.3 Ma (Jurassic).

Literature. Dolin (1980: 70): original description [53]; Korneev and Cate (2005: 14): checklist [120].

Remark. Pronotosternal sutures of this species are closed (Figure 76 in [53]) which does not correspond neither with the diagnosis of genus *Litholacon* nor with Agrypnini.

 

***Litholacon derumpens* Dolin, 1980**

*Litholacon derumpens* Dolin, 1980: 67/68 [53].

Type material. Holotype, sex unknown, exoskeleton, compression fossil, No. 2554/699 (PIN).

Fossil deposit/age. Kazakhstan: Karabastau Formation, Karatau, Mikhailovka; 166.1–157.3 Ma (Jurassic).

Literature. Dolin (1980: 68): original description [53]; Carpenter (1992: 305): generic catalogue [68]; Korneev and Cate (2005: 10): checklist [120]; Kundrata et al. (2020: 5): generic catalogue [12].

Remark. This species was designated as the type species of *Litholacon* ([53], p. 67) and the concept of the species is clear from Figure 72 (without a page number), with the legend as follows: “*Litholacon derumpens* Dolin, sp. nov., holotype Nr. 2554/699”. Dolin obviously described *L. derumpens* on page 68 (based on the holotype number and reference to Figure 72) but under the name *L. panphilovi*, which he used also for the other species on the same page.

 

***Litholacon exilis* Dolin, 1980**

*Litholacon exilis* Dolin, 1980: 71 [53].

Type material. Holotype, sex unknown, exoskeleton, compression fossil, No. 2997/2005 (PIN).

Fossil deposit/age. Kazakhstan: Karabastau Formation, Karatau, Mikhailovka; 166.1–157.3 Ma (Jurassic).

Literature. Dolin (1980: 71): original description [53]; Korneev and Cate (2005: 15): checklist [120].

Remark. Pronotosternal sutures of this species are closed (Figure 78 in [53]) which does not correspond neither with the diagnosis of genus *Litholacon* nor with Agrypnini. The systematic placement of this species requires further study.

 

***Litholacon major* Dolin, 1980**

*Litholacon major* Dolin, 1980: 71 [53].

Type material. Holotype, sex unknown, exoskeleton, compression fossil, No. 2239/1452 (part + counterpart) (PIN).

Fossil deposit/age. Kazakhstan: Karabastau Formation, Karatau, Mikhailovka; 166.1–157.3 Ma (Jurassic).

Literature. Dolin (1980: 71): original description [53]; Korneev and Cate (2005: 19): checklist [120].

 

***Litholacon panphilovi* Dolin, 1980**

*Litholacon panphilovi* Dolin, 1980: 68 [53].

Type material. Holotype, sex unknown, exoskeleton, compression fossil, No. 2066/2400 (PIN). Paratype, sex unknown, exoskeleton, compression fossil, No. 2239/1407.

Fossil deposit/age. Kazakhstan: Karabastau Formation, Karatau, Mikhailovka; 166.1–157.3 Ma (Jurassic).

Literature. Dolin (1980: 68): original description [53]; Korneev and Cate (2005: 21): checklist [120].

 

***Litholacon ohiri* Dolin, 1980**

*Litholacon ohiri* Dolin, 1980: 69 [53].

Type material. Holotype, sex unknown, exoskeleton, compression fossil, No. 2997/420 (PIN).

Fossil deposit/age. Kazakhstan: Karabastau Formation, Karatau, Mikhailovka; 166.1–157.3 Ma (Jurassic).

Literature. Dolin (1980: 69): original description [53]; Korneev and Cate (2005: 21): checklist [120].

Remark. Pronotosternal sutures of this species are closed (Figure 74 in [53]) which does not correspond neither with the diagnosis of genus *Litholacon* nor with Agrypnini. The systematic placement of this species requires further study.

 

***Litholacon petrorsus* Dolin, 1980**

*Litholacon petrorsus* Dolin, 1980: 69 [53].

Type material. Holotype, sex unknown, exoskeleton, compression fossil, No. 2066/2456 (PIN).

Fossil deposit/age. Kazakhstan: Karabastau Formation, Karatau, Mikhailovka; 166.1–157.3 Ma (Jurassic).

Literature. Dolin (1980: 69): original description [53]; Korneev and Cate (2005: 21): checklist [120].

 

- **Genus *Macropunctum* Tröster, 1991**

*Macropunctum* Tröster, 1991: 100 [62]. Type species: *Macropunctum messelense* Tröster, 1991: 106 [62]. For more information, see Kundrata et al. [12].

Remark. This genus is similar in the body proportions and the structure of thorax and elytra to the Nearctic *Agrypnus rectangularis* (Say, 1825) and related species (previously assigned under a separate genus *Colaulon* Arnett, 1952). The relationships between these groups require more detailed examination.

 

***Macropunctum angulosum* Tröster, 1999**

*Macropunctum angulosum* Tröster 1999: 13 [67].

Type material. Holotype, sex unknown, exoskeleton, compression fossil, SMF MeI 4120 (FIS). Two paratypes, exoskeletons, compression fossils, sex unknown, SMF MeI 381, 796 (FIS).

Fossil deposit/age. Germany: Messel Formation, Grube Messel Pit (type locality: E10, 3.52–3.66 below alpha); 48.6–40.4 Ma (Eocene).

Literature. Tröster (1999: 13): original description [67]; Wappler (2003: 88): revision [69].

 

***Macropunctum angustiscutellum* Tröster, 1994**

*Macropunctum angustiscutellum* Tröster, 1994: 154 [66].

Type material. Holotype, sex unknown, exoskeleton, compression fossil, SMF MeI 2571 (FIS). 10 paratypes, sex unknown, exoskeletons, compression fossils, SMF MeI 637, 3357, 233, 253, 373, 794, 1109, 2262, 163 (originally as a paratype of *M. messelense*), 271 (originally as a paratype of *M. messelense*) (FIS).

Fossil deposit/age. Germany: Messel Formation, Grube Messel Pit (type locality: E9, 0.43–0.97 m above alpha); 48.6–40.4 Ma (Eocene).

Literature. Tröster (1994: 154): original description [66]; Wappler (2003: 89): revision [69].

 

***Macropunctum densipunctum* Wappler, 2003**

*Macropunctum densipunctum* Wappler, 2003: 87 [69].

*Macropunctum densepunctum*: Kirejtshuk et al., 2019: 48 [70] [unavailable name, incorrect subsequent spelling not in prevailing usage; [129], Art. 33.3].

Type material. Holotype, sex unknown, exoskeleton, PE_2000/955, LS (NHMM). Paratype, sex unknown, exoskeleton, abdomen, PE_2000/744 a+b, LS (NHMM).

Fossil deposit/age. Germany: Eifel Formation, Eckfeld Maar; 48.6–40.4 Ma (Eocene).

Literature. Wappler (2003: 87): original description [69]; Kirejtshuk et al. (2019: 48): remark [70].

 

***Macropunctum eckfeldi* Tröster, 1992**

*Macropunctum eckfeldi* Tröster, 1992: 114 [63].

Type material. Holotype, sex unknown, exoskeleton, compression fossil, PE 1990/974-LS (NHMM).

Fossil deposit/age. Germany: Eifel Formation, Eckfeld Maar; 48.6–40.4 Ma (Eocene).

Literature. Tröster (1992: 114): original description [63]; Wappler (2003: 89): revision [69].

 

***Macropunctum eocaenicum* (Meunier, 1921)**

*Ancylochira eocaenica* Meunier, 1921: 7 [130].

*Macropunctum eocaenicum*: Tröster, 1991: 102 [62].

Type material. Lectotype, sex unknown, exoskeleton, compression fossil, HLMD Me 1082 (HLMD). Nine paralectotypes, sex unknown, exoskeletons, compression fossils, HLMD 937, 1225, 1338, 593, 1182, 4090, 1393, 925, 927 (HLMD).

Fossil deposit/age. Germany: Messel Formation, Grube Messel Pit; 48.6–40.4 Ma (Eocene).

Literature. Meunier (1921: 7): original description [130]; Tröster (1991: 102): revision [62]; Tröster (1994: 148): remark [66]; Wappler (2003: 89): revision [69]; Schimmel (2005: 27): remark [91]; Schimmel and Tarnawski (2010: 363): remark [131]; Schimmel and Tarnawski (2012: 265): remark [132].

 

***Macropunctum latiscutellum* Tröster, 1994**

*Macropunctum latiscutellum* Tröster, 1994: 151 [66].

Type material. Holotype, sex unknown, exoskeleton, compression fossil, SMF MeI 628 (originally as a paratype of *M. messelense*) (FIS). 13 paratypes, sex unknown, exoskeletons, compression fossils, SMF MeI 696 (originally as a paratype of *M. messelense*), 2671, 5, 14, 230, 244, 256, 627, 1055, 1399, 2591, 2882, 3411 (FIS).

Fossil deposit/age. Germany: Messel Formation, Grube Messel Pit (type locality: F11, 1.0 m above alpha); 48.6–40.4 Ma (Eocene).

Literature. Tröster (1994: 151): original description [66].

 

***Macropunctum messelense* Tröster, 1991**

*Macropunctum messelense* Tröster, 1991: 106 [62].

Type material. Holotype, sex unknown, exoskeleton, compression fossil, SMF MeI 2392 (FIS). 10 paratypes, sex unknown, exoskeletons, compression fossils, SMF MeI 675, 2293, 8, 760, 911, 1103, 1132, 1351, 2487, 2490 (FIS).

Fossil deposit/age. Germany: Messel Formation, Grube Messel Pit (type locality: E11, 5.06–5.71 m below alpha); 48.6–40.4 Ma (Eocene).

Literature. Tröster (1991: 106): original description [62]; Tröster (1994: 148): remark [66]; Tröster (1999: 13): remark [67]; Wappler (2003: 89): revision [69]; Kirejtshuk et al. (2019: 48): remark [70]; Kundrata et al. (2020: 5): generic catalogue [12].

 

***Macropunctum meunieri* Tröster, 1991**

*Macropunctum meunieri* Tröster, 1991: 112 [62].

Type material. Holotype, sex unknown, exoskeleton, compression fossil, SMF MeI 2627 (FIS). Four paratypes, sex unknown, exoskeletons, compression fossils, SMF MeI 430, 536, 669, 2136 (FIS).

Fossil deposit/age. Germany: Messel Formation, Grube Messel Pit (type locality: grid square i8, 0.26–1.29 m above alpha); 48.6–40.4 Ma (Eocene).

Literature. Tröster (1991: 112): original description [62]; Wappler (2003: 89): revision [69].

 

***Macropunctum minutum* (Meunier, 1921)**

*Ancylochira minuta* Meunier, 1921: 8 [130].

*Macropunctum minutum*: Tröster, 1994: 160 [66].

Type material. Lectotype, sex unknown, exoskeleton, compression fossil, HLMD Me 1206. (HLMD). Two paralectotypes reported by Tröster [66], sex unknown, exoskeletons, compression fossils, HLMD Me 1276 and 1124 (HLMD). Additional 12 specimens listed in the original description (988, 877, 1087, 1261, 1227, 1263, 1229, 1140, 1077, 1226, 4142, 648) [130].

Fossil deposit/age. Germany: Messel Formation, Grube Messel Pit; 48.6–40.4 Ma (Eocene).

Literature. Meunier (1921: 8): original description [130]; Tröster (1994: 160): remark, nomenclature [66].

 

***Macropunctum promptum* (Meunier, 1921)**

*Ancylochira prompta* Meunier, 1921: 8 [130].

*Macropunctum promptum*: Tröster, 1991: 105 [62].

Type material. Holotype, sex unknown, exoskeleton, compression fossil, HMLD Me 1003 (HLMD). Eight paratypes, sex unknown, exoskeletons, compression fossils, HMLD Me 755, 1330, 650, 1093, 809, 932, 1184, 952 (HLMD). Both Meunier [130] and Tröster [62] listed nine paratypes but No. 650 was mentioned twice.

Fossil deposit/age. Germany: Messel Formation, Grube Messel Pit; 48.6–40.4 Ma (Eocene).

Literature. Meunier (1921: 8): original description [130]; Tröster (1991: 105): remark [62], Tröster (1994: 160): remark [66].

 

***Macropunctum rebugense* Tröster, 1994**

*Macropunctum rebugense* Tröster, 1994: 158 [66].

Type material. Holotype, sex unknown, exoskeleton, compression fossil, SMF MeI 1145 (FIS). Three paratypes, sex unknown, exoskeletons, compression fossils, SMF MeI 143, 699, and 3474 (FIS).

Fossil deposit/age. Germany: Messel Formation, Grube Messel Pit (type locality: 5, E15, 1 m below to 2 m above alpha); 48.6–40.4 Ma (Eocene).

Literature. Tröster (1994: 158): original description [66]; Wappler (2003: 89): revision [69].

 

***Macropunctum rossi* Alekseev, 2019**

*Macropunctum rossi* Alekseev in Kirejtshuk et al., 2019: 48 [70].

Type material. Holotype, sex unknown, exoskeleton, compression fossil, NHMUK I.10085 (BMNH).

Fossil deposit. United Kingdom: England, Isle of Wight, Bouldnor Formation, Bembridge Marls; 38.0–33.9 Ma (Eocene).

Literature. Kirejtshuk et al. (2019: 48): original description [70].

 

***Macropunctum senckenbergi* Tröster, 1994**

*Macropunctum senckenbergi* Tröster, 1994: 148 [66].

Type material. Holotype, sex unknown, exoskeleton, compression fossil, SMF MeI 297 (FIS). Four paratypes, sex unknown, exoskeletons, compression fossils, SMF MeI 672, 2549, 3225, 3232 (FIS).

Fossil deposit/age. Germany: Messel Formation, Grube Messel Pit (type locality: grid square F11, 1.0 m above alpha); 48.6–40.4 Ma (Eocene).

Literature. Tröster (1991: 104): description, without formal name [62]; Tröster (1994: 148): original description [66]; Wappler (2003: 89): revision [69].

 

- **Genus *Plagioraphes* Iablokoff-Khnzorian, 1961**

*Plagioraphes* Iablokoff-Khnzorian, 1961: 84 [47]. Type species: *Plagioraphes fasciatus* Iablokoff-Khnzorian, 1961. For more information, see Kundrata et al. [12].

 

***Plagioraphes fasciatus* Iablokoff-Khnzorian, 1961**

*Plagioraphes fasciatus* Iablokoff-Khnzorian, 1961: 85 [47].

Type material. Holotype, sex unknown, exoskeleton, amber inclusion, No. 364/346 (PIN).

Fossil deposit/age. Baltic amber; 38.0–33.9 Ma (Eocene).

Literature. Iablokoff-Khnzorian (1961: 85): original description [47]; Larsson (1978: 153): catalogue [48]; Spahr (1981: 49): catalogue [49]; Keilbach (1982: 246): catalogue [133]; Carpenter (1992: 305): generic catalogue [68]; Alekseev (2013: 7): checklist [92]; Kundrata et al. (2020: 5): generic catalogue [12].

#### 3.1.2. Tribe Cryptocardiini Dolin, 1980

Cryptocardiini Dolin, 1980: 74 [53]. Type genus: *Cryptocardius* Dolin, 1980: 74 [53].

Remark. This group most probably does not belong to Agrypninae. Dolin [53] compared Cryptocardiini with Oophorini due to similar habitus, closed pronotosternal sutures, and structure of prosternum (without further details). He wrote that Cryptocardiini differ from Oophorini in the narrowed mesoventrite, less broadened metacoxal plates, and simple tarsi. However, Dolin [53] mentioned that the shape of antennae (i.e., antennomeres being shortened from antennomere VI to apex) and the cordate scutellar shield of *Cryptocardius* are not typical for Agrypninae at all. Drawings of *Cryptocardius* in Dolin [53] show that this click-beetle has enlarged pronotum which is sinuate near posterior angles, and has short basal furrows. Additionally, there is a short incision near basal furrow on the posterior angle of pronotum. These characters suggest that *Cryptocardius* might have been related to Hypnoidini.

 

- **Genus *Cryptocardius* Dolin, 1980**

*Cryptocardius* Dolin, 1980: 74 [53]. Type species: *Cryptocardius mirabilis* Dolin, 1980: 75 [53]. For more information, see Kundrata et al. [12].

 

***Cryptocardius mirabilis* Dolin, 1980**

*Cryptocardius mirabilis* Dolin, 1980: 75 [53].

Type material. Holotype, sex unknown, exoskeleton, compression fossil, No. 2554/649 (part + counterpart) (PIN).

Fossil deposit/age. Kazakhstan: Karabastau Formation, Karatau, Mikhailovka; 166.1–157.3 Ma (Jurassic).

Literature. Dolin (1980: 75): original description [53]; Carpenter (1992: 304): generic catalogue [68]; Korneev and Cate (2005: 10): checklist [120]; Alekseev (2011: 423): checklist [75]; Kundrata et al. (2020: 6): generic catalogue [12].

#### 3.1.3. Tribe Hemirhipini Candèze, 1857 *

Hémirhipides Candèze, 1857: 199 [116]. Type genus: *Hemirhipus* Berthold, 1827: 336 [134]. For more information, including synonyms, see Kundrata et al. [109].

 

- Genus *Alaus* Eschscholtz, 1829 *

*Alaus* Eschscholtz, 1829: 33 [117]. Type species: *Elater oculatus* Linnaeus, 1758: 404 [115]. For more information, including synonyms, see Kundrata et al. [109].

 

***Alaus spectabilis* (Heer, 1865)**

*Elater (Alaus) spectabilis* Heer, 1865: 378 [17].

*Alaus spectabilis*: Handlirsch, 1907: 744 [127].

Type material. Holotype, sex unknown, compression fossil (UZH).

Fossil deposit/age. Germany: Upper Freshwater-Molasse Formation, Öhningen; 12.7–11.608 Ma (Miocene).

Literature. Heer (1865: 378): original description, figure [17]; Heer (1870: 75): remark [18]; Heer (1872: 463): remark [135]; Heer (1876: 34): remark [136]; Heer (1883: 404): figure [137]; Scudder (1891: 518): catalogue [24]; Handlirsch (1907: 744): catalogue [127].

#### 3.1.4. Tribe Oophorini Gistel, 1848 *

Oophoridae Gistel, 1848: 5 [138]. Type genus: *Oophorus* Dejean, 1833: 93 [139] (syn. of *Aeolus* Eschscholtz, 1829: 33 [117]). For more information, including synonyms, see Kundrata et al. [109].

 

- Genus *Monocrepidius* Eschscholtz, 1829 *

*Monocrepidius* Eschscholtz, 1829: 31 [117]. Type species: *Monocrepidius pallipes* Eschscholtz, 1829: 32 [117]. For more information, including synonyms, see Kundrata et al. [109].

 

***Monocrepidius dubiosus* Wickham, 1916**

*Monocrepidius dubiosus* Wickham, 1916: 508 [28].

Type material. Holotype, sex unknown, compression fossil, No. 90,483 (USNM).

Fossil deposit/age. USA: Colorado, Florissant Formation, Wilson Ranch; 37.2–33.9 Ma (Eocene).

Literature. Wickham (1916: 508): original description [28]; Wickham (1920: 354): catalogue [29].

#### 3.1.5. Tribe Pseudomelanactini Arnett, 1967 *

Pseudomelanactini Arnett, 1967: 111 [140]. Type genus: *Pseudomelanactes* Mathieu, 1961: 474 [141] (synonym of *Anthracalaus* Fairmaire, 1888: 349 [142]).

 

- Genus *Lanelater* Arnett, 1952 *

*Lanelater* Arnett, 1952: 105 [143]. Type species: *Agrypnus schotti* LeConte, 1853: 492 [144]. For more information, including synonyms, see Kundrata et al. [109].

 

***Lanelater nicoleae* Wappler, 2003**

*Lanelater nicoleae* Wappler, 2003: 90 [69].

Type material. Holotype, sex unknown, exoskeleton, compression fossil, PE_2000/349 a+b LS (NHMM). Three paratypes, sex unknown, exoskeletons, compression fossils, PE_1994/79 a+b, LS, PE_1993/256 a+b, LS, PE_1992/79, LS (NHMM).

Fossil deposit/age. Germany: Eifel Formation, Eckfeld Maar; 48.6–40.4 Ma (Eocene).

Literature. Wappler (2003: 90): original description [69].

 

***Lanelater verae* Tröster, 1993**

*Lanelater verae* Tröster, 1993: 51 [64].

Type material. Holotype, sex unknown, exoskeleton, compression fossil, SMF MeI 3735 (FIS). Five paratypes, sex unknown, exoskeletons, compression fossils, SMF MeI 1384, SMF MeI 1969, SMF MeI 1593, SMF MeI 1978, SMF MeI 226 (FIS).

Fossil deposit/age. Germany: Messel Formation, Grube Messel Pit (type locality: grid square H12, 0.00–0.26 m below horizon M); 48.6–40.4 Ma (Eocene).

Literature. Tröster (1993: 51): original description [64]; Wappler (2003: 92): remark [69].

#### 3.1.6. Tribe Pyrophorini Candèze, 1863 *

Pyrophorites Candèze, 1863: 3 [145]. Type genus: *Pyrophorus* Billberg, 1820: 20 [146]. For more information, including synonyms, see Kundrata et al. [109].

 

- **Genus *Eopyrophorus* Haupt, 1950**

*Eopyrophorus* Haupt, 1950: 101 [44]. Type species: *Eopyrophorus mixtus* Haupt, 1950: 107 [44]. For more information, see Kundrata et al. [12].

Remark. Although this genus might indeed belong to Pyrophorini, its placement needs further examination since important tribal characters were not discussed by Haupt [44].

 

***Eopyrophorus mixtus* Haupt, 1950**

*Eopyrophorus mixtus* Haupt, 1950: 107 [44].

Type material. Type, sex unknown, compression fossil (GIH).

Fossil deposit/age. Germany: Geiseltal; 47.8–41.3 Ma (Eocene).

Literature. Haupt (1950: 107): original description [44]; Haupt (1956: 48): catalogue [147]; Carpenter (1992: 304): generic catalogue [68]; Kundrata et al. (2020: 6): generic catalogue [12].

### 3.2. Subfamily Cardiophorinae Candèze, 1859 *

Cardiophorites Candèze, 1859: 4 [148]. Type genus: *Cardiophorus* Eschscholtz, 1829: 34 [117]. For more information, including synonyms, see Bouchard et al. [110] and Douglas [108].

Remark. Type material of most species should be examined in order to confirm their placement in Cardiophorinae. Some species may represent Negastriinae. Considering the current limits and diagnoses of cardiophorine genera, it is impossible for most species to assign them to a proper genus based on available characters [108].

 

- Genus *Cardiophorus* Eschscholtz, 1829 *

*Cardiophorus* Eschscholtz, 1829: 34 [117]. Type species: *Elater thoracicus* Fabricius, 1801: 236 [149] (synonym of *Cardiophorus gramineus* (Scopoli, 1763: 95) [150]). For more information, see Douglas [108].

 

***Cardiophorus braunii* Heer, 1847**

*Cardiophorus braunii* Heer, 1847: 134 [14].

*Cardiophorus brauni*: Giebel (1852: 651) [126] [unavailable name, incorrect subsequent spelling not in prevailing usage; [129], Art. 33.4].

Type material. Holotype, sex unknown, compression fossil (SMNK).

Fossil deposit/age. Germany: Upper Freshwater-Molasse Formation, Öhningen; 12.7–11.608 Ma (Miocene).

Literature. Heer (1847: 134): original description [14]; Giebel (1852: 651): catalogue [126]; Giebel (1856: 97): redescription [16]; Scudder (1891: 486): catalogue [24]; Handlirsch (1907: 746): catalogue [127]; Cockerell (1926: 10): comparison with other species [35].

 

***Cardiophorus cockerelli* Wickham, 1916**

*Cardiophorus cockerelli* Wickham, 1916: 503 [28].

Type material. Holotype, sex unknown, exoskeleton, compression fossil, MCZ 2765 (=1916 Scudder coll.) (MCZ).

Fossil deposit. USA: Colorado, Florissant Formation, Florissant; 37.2–33.9 Ma (Eocene).

Literature. Wickham (1916: 503): original description [28]; Wickham (1920: 354): catalogue [29].

 

***Cardiophorus deprivatus* Wickham, 1916**

*Cardiophorus deprivatus* Wickham, 1916: 504 [28].

Type material. Holotype, sex unknown, compression fossil, No. 8206 (99/127) (CUB).

Fossil deposit/age. USA: Colorado, Florissant Formation, Chadronian, Station 13; 37.2–33.9 Ma (Eocene).

Literature. Wickham (1916: 504): original description [28]; Wickham (1920: 354): catalogue [29].

 

***Cardiophorus exhumatus* Cockerell, 1926**

*Cardiophorus exhumatus* Cockerell, 1926: 9 [35].

Type material. Holotype, sex unknown, exoskeleton, compression fossil, No. 69614 (USNM).

Fossil deposit/age. USA: Colorado, Green River Formation, head of East Alkali Creek, approximately 8 miles south of De Beque; 50.3–46.2 Ma (Eocene).

Literature. Cockerell (1926: 9): original description [35]; Wickham (1927: 55): catalogue [151].

 

***Cardiophorus florissantensis* Wickham, 1916**

*Cardiophorus florissantensis* Wickham, 1916: 502 [28].

Type material. Holotype, sex unknown, compression fossil, No. 8205 (CUB).

Fossil deposit/age. USA: Colorado, Florissant Formation, Florissant, Station 13; 37.2–33.9 Ma (Eocene).

Literature. Wickham (1916: 502): original description [28]; Wickham (1920: 354): catalogue [29].

 

***Cardiophorus lithographus* Wickham, 1916**

*Cardiophorus lithographus* Wickham, 1916: 501 [28].

Type material. Holotype, sex unknown, compression fossil, 90,611 (USNM).

Fossil deposit/age. USA: Colorado, Florissant Formation, Wilson Ranch; 37.2–33.9 Ma (Eocene).

Literature. Wickham (1916: 401): original description [28]; Cockerell (1926: 10): comparison with other species [29].

 

***Cardiophorus requiescens* Wickham, 1916**

*Cardiophorus requiescens* Wickham, 1916: 504 [28].

Type material. Holotype, sex unknown, compression fossil, 90,612 (USNM).

Fossil deposit/age. USA: Colorado, Florissant Formation, Wilson Ranch; 37.2–33.9 Ma (Eocene).

Literature. Wickham (1916: 504): original description [28]; Wickham (1920: 354): catalogue [29].

 

***Cardiophorus yatsenkokhmelevskyi* Iablokoff-Khnzorian, 1961**

*Cardiophorus yatsenkokhmelevskyi* Iablokoff-Khnzorian, 1961: 94 [47].

Type material. Holotype, sex unknown, exoskeleton, amber inclusion, No. 364/656 (PIN).

Fossil deposit/age. Baltic amber; 38.0–33.9 Ma (Eocene).

Literature. Iablokoff-Khnzorian (1961: 94): original description [47]; Larsson (1978: 153): catalogue [48]; Spahr (1981: 46): catalogue [49]; Keilbach (1982: 246): catalogue [133]; Hawkeswood et al. (2009: 189): catalogue [105]; Alekseev (2013: 7): checklist [92].

 

- Genus *Horistonotus* Candèze, 1860 *

*Horistonotus* Candèze, 1860: 243 [152]. Type species: *Horistonotus flavidus* Candèze, 1860: 250 [152]. For more information, see Douglas [108].

 

***Horistonotus coloradensis* Wickham, 1916**

*Horistonotus coloradensis* Wickham, 1916: 505 [28].

Type material. Holotype, sex unknown, compression fossil, No. 90,547 (USNM).

Fossil deposit/age. USA: Colorado, Florissant Formation; 37.2–33.9 Ma (Eocene).

Literature. Wickham (1916: 505): original description [28]; Wickham (1920: 354): catalogue [29].

### 3.3. Subfamily Dendrometrinae Gistel, 1848 *

Dendrometridae Gistel, 1848: 5 [138]. Type genus: *Dendrometrus* Gistel, 1848: 5 [138]. For more information, including synonyms, see Bouchard et al. [110].

#### 3.3.1. Tribe Dendrometrini Gistel, 1848 *

Dendrometridae Gistel, 1848: 5 [138]. Type genus: *Dendrometrus* Gistel, 1848: 5 [138]. For more information, including synonyms, see Bouchard et al. [110].

 

- Genus *Athous* Eschscholtz, 1829 *

*Athous* Eschscholtz, 1829: 33 [117]. Type species: *Elater vittatus* Fabricius, 1792: 224 [153]. For more information, including synonyms, see Sánchez-Ruiz [121] and Cate [107].

 

**Subgenus *Athousiomorphus* Iablokoff-Khnzorian, 1961**

*Athousiomorphus* Iablokoff-Khnzorian, 1961: 92 [47]. Type species: *Athous* (*Athousiomorphus*) *olgae* Iablokoff-Khnzorian, 1961: 92 [47]. For more information, see Kundrata et al. [12].

 

***Athous* (*Athousiomorphus*) *olgae* Iablokoff-Khnzorian, 1961**

*Athous* (*Athousiomorphus*) *olgae* Iablokoff-Khnzorian, 1961: 92 [47].

*Athous olgae* Larsson, 1978: 153 [48].

Type material. Holotype, male, exoskeleton, amber inclusion, No. 364/655 (PIN).

Fossil deposit/age. Baltic amber; 38.0–33.9 Ma (Eocene).

Literature. Iablokoff-Khnzorian (1961: 92): original description [47]; Larsson (1978: 153): catalogue [48]; Spahr (1981: 46): catalogue [49]; Keilbach (1982: 246): catalogue [133]; Schimmel (2005: 27): remark [91]; Alekseev (2013: 7): checklist [92]; Kundrata et al. (2020: 7): generic catalogue [12].

 

**Subgenus *incertae sedis***

 

***Athous contusus* Wickham, 1916**

*Athous contusus* Wickham, 1916: 519 [28].

Type material. Holotype, sex unknown, exoskeleton, compression fossil, MCZ 2727 (=8346 in Scudder coll.) (MCZ).

Fossil deposit/age. USA: Colorado, Florissant Formation, Florissant; 37.2–33.9 Ma (Eocene).

Literature. Wickham (1916: 519): original description [28]; Wickham (1920: 354): catalogue [29].

 

***Athous fractus* Wickham, 1916**

*Athous fractus* Wickham, 1916: 519 [28].

Type material. Holotype, sex unknown, compression fossil, No. 8240 (CUB).

Fossil deposit/age. USA: Colorado, Florissant Formation, Florissant, Station 14; 37.2–33.9 Ma (Eocene).

Literature. Wickham (1916: 519): original description [28]; Wickham (1920: 354): catalogue [29].

Remark. Based on the body size, general habitus, structure of antenna and thorax, this species might belong to Prosternini or Selatosomini. Most striking character which challenges the position of this species in *Athous* is the pronotum, which is rather broad, more or less rounded at sides and sinuate near posterior angles (usually parallel sided and rather narrowed in *Athous*), and with each posterior angle bearing a sublateral carina (not typical for *Athous*). We prefer to postpone any taxonomic changes pending a comprehensive review including the type material.

 

***Athous holmgreni* (Heer, 1870)**

*Elater holmgreni* Heer, 1870: 75 [18].

*Athous holmgreeni*: Birket-Smith, 1977: 18 [154] [unavailable name, incorrect subsequent spelling not in prevailing usage; [129], Art. 33.3].

Type material. Holotype, sex unknown, elytron, compression fossil, No. 53 (SMNH).

Fossil deposit/age. Norway: Svalbard and Jan Mayen, Firkanten Formation, Cap Staratschin; 66.0–59.2 Ma (Paleocene).

Literature. Heer (1870: 75): original description [18]; Scudder (1891: 517): catalogue [24]; Handlirsch (1907: 745): catalogue [127]; Birket-Smith (1977: 17): taxonomic remark [154]; Tröster (1994: 39): remark [65].

Remark. The description of this species was based on a part of isolated elytron and, therefore, its generic attribution is rather problematic.

 

***Athous lethalis* Wickham, 1916**

*Athous lethalis* Wickham, 1916: 518 [28].

Type material. Holotype, sex unknown, exoskeleton, compression fossil, MCZ 2728 and 2729 (=8464 and 8713 in Scudder coll.) (MCZ).

Fossil deposit. USA: Colorado, Florissant Formation, Florissant; 37.2–33.9 Ma (Eocene).

Literature. Wickham (1916: 518): original description [28]; Wickham (1920: 354): catalogue [29].

 

- Genus *Limonius* Eschscholtz, 1829 *

*Limonius* Eschscholtz, 1829: 33 [117]. Type species: *Elater minutus* Linnaeus, 1758: 406 [115]. For more information, including synonyms, see Sánchez-Ruiz [121], Cate [107] and Etzler [155].

 

**Subgenus *Paralimonius* Iablokoff-Khnzorian, 1961**

*Paralimonius* Iablokoff-Khnzorian, 1961: 91 [47]. Type species: *Limonius* (*Paralimonius*) *barovskyi* Iablokoff-Khnzorian, 1961: 91 [47]. For more information, see Kundrata et al. [12].

 

***Limonius (Paralimonius) barovskyi* Iablokoff-Khnzorian, 1961**

*Limonius* (*Paralimonius*) *barovskyi* Iablokoff-Khnzorian, 1961: 91 [47].

*Limonius barovskyi:* Larsson, 1978: 153 [48].

Type material. Holotype, sex unknown, exoskeleton, amber inclusion, No. 364/654 (PIN).

Fossil deposit/age. Baltic amber; 38.0–33.9 Ma (Eocene).

Literature. Iablokoff-Khnzorian (1961: 91): original description [47]; Larsson (1978: 153): catalogue [48]; Spahr (1981: 48): catalogue [49]; Keilbach (1982: 246): catalogue [133]; Schimmel (2005: 27): remark [91]; Alekseev (2013: 7): checklist [92]; Kundrata et al. (2020: 7): generic catalogue [12].

 

**Subgenus *incertae sedis***

 

***Limonius aboriginalis* Wickham, 1916**

*Limonius aboriginalis* Wickham, 1916: 514 [28].

Type material. Holotype, sex unknown, compression fossil, No. 90,474 (USNM).

Fossil deposit/age. USA: Colorado, Florissant Formation, Wilson Ranch, Florissant; 37.2–33.9 Ma (Eocene).

Literature. Wickham (1916: 514): original description [28]; Wickham (1920: 354): catalogue [29].

Remark. Representatives of *Limonius* and related genera have more elongated elytra and more or less campaniform or parallel-sided pronotum, whereas this species has elytra only slightly elongate, and the pronotum arcuate at sides and sinuate near posterior angles. Such body proportions and shape of thorax are usually found in Cardiophorinae and Negastriinae. Based on the image of prothorax [28], *L. aboriginalis* is most probably a member of Cardiophorinae; its prosternum is narrow and prosternal sutures almost parallel sided while in Negastriinae the prosternum is rather broad and prosternal sutures curved outward. However, we prefer to postpone any taxonomic changes pending a comprehensive review including the type material.

 

***Limonius florissantensis* Wickham, 1916**

*Limonius florissantensis* Wickham, 1916: 515 [28].

Type material. Two syntypes (one with counterpart), sex unknown, compression fossils, No. 90,473 (USNM).

Fossil deposit/age. USA: Colorado, Florissant Formation, Wilson Ranch, Florissant; 37.2–33.9 Ma (Eocene).

Literature. Wickham (1916: 515): original description [28]; Wickham (1920: 354): catalogue [29].

Remark. Representatives of *Limonius* and related genera have more elongated elytra and more or less campaniform or parallel-sided pronotum, whereas this species has elytra only slightly elongate, and the pronotum arcuate at sides and sinuate near posterior angles. Such body proportions and shape of thorax are usually found in Cardiophorinae and Negastriinae. Based on the prothorax image in Wickham [28], *L. florissantensis* is most probably a member of Negastriinae as its pronotum is less globular than in typical Cardiophorinae. However, the type material should be thoroughly examined before any taxonomic change can be made.

 

***Limonius impunctus* Scudder, 1895**

*Limonius impunctus* Scudder, 1895: 37 [156].

Type material. Holotype, sex unknown, elytron, compression fossil, No. 100a, b (GSC).

Fossil deposit/age. Canada: Allenby Formation (Princeton Group), North Fork of Similkameen River; 56.0–47.8 Ma (Eocene).

Literature. Scudder (1895: 37): original description [156]; Scudder (1900: 96): catalogue [157]; Handlirsch (1907: 746): catalogue [127]; Wickham (1920: 354): catalogue [29].

Remark. The generic attribution of this species is unclear as it was described based only on elytral characters.

 

***Limonius optabilis* Heer, 1847**

*Limonius optabilis* Heer, 1847: 137 [14].

Type material. Holotype, sex unknown, compression fossil (ETH).

Fossil deposit/age. Germany: Upper Freshwater-Molasse Formation, Öhningen, Upper Öhningen beds; 12.7–11.608 Ma (Miocene).

Literature. Heer (1847: 137): original description [14]; Giebel (1852: 651): catalogue [126]; Giebel (1856: 95): redescription [16]; Heyden (1862: 69): remark [158]; Scudder (1891: 547): catalogue [24]; Handlirsch (1907: 746): catalogue [127].

Remark. Generic attribution of this species is doubtful. It might belong to Agrypnini based on the body proportions and the structure of thorax, especially as figured in Figure 6b in Heer [14]. Note, that drawing in Figure 6c in Heer [14] differs considerably in many features from Figure 6b in the same study.

 

***Limonius praecursor* Wickham, 1916**

*Limonius praecursor* Wickham, 1916: 516 [28].

Type material. Holotype, sex unknown, exoskeleton, compression fossil, MCZ 2730 and 2731 (=9417 and 10,558 in Scudder coll.) (MCZ).

Fossil deposit/age. USA: Colorado, Florissant Formation, Florissant; 37.2–33.9 Ma (Eocene).

Literature. Wickham (1916: 516): original description [28]; Wickham (1920: 354): catalogue [29].

 

***Limonius shoshonis* Wickham, 1916**

*Limonius shoshonis* Wickham, 1916: 517 [28].

Type material. Holotype, sex unknown, compression fossil, No. 8251 (58) (CUB).

Fossil deposit/age. USA: Colorado, Florissant Formation, Florissant, Station 14; 37.2–33.9 Ma (Eocene).

Literature. Wickham (1916: 517): original description [28]; Wickham (1920: 354): catalogue [29].

Remark. Representatives of *Limonius* and related genera have more elongated elytra and more or less campaniform or parallel-sided pronotum, whereas this species has elytra only slightly elongate, and the pronotum arcuate at sides and sinuate near posterior angles. Such body proportions and shape of thorax are usually found in Cardiophorinae and Negastriinae. Based on the prothorax image in Wickham [28], *L. shoshonis* is most probably a member of Negastriinae as its pronotum is less globular than in typical Cardiophorinae. However, the type material should be thoroughly examined before any taxonomic change can be made.

 

***Limonius volans* Wickham, 1916**

*Limonius volans* Wickham, 1916: 517 [28].

Type material. Holotype, sex unknown, compression fossil, No. 8252 (CUB).

Fossil deposit/age. USA: Colorado, Florissant Formation, Florissant, Station 14; 37.2–33.9 Ma (Eocene).

Literature. Wickham (1916: 517): original description [28]; Wickham (1920: 354): catalogue [29].

 

#### 3.3.2. Tribe Dimini Candèze, 1863 *

Dimites Candèze, 1863: 237 [145]. Type genus: *Dima* Charpentier, 1825: 191 [159]. For more information, see Kundrata et al. [12,160].

 

- **Genus *Alaodima* Dolin, 1980**

*Alaodima* Dolin, 1980: 75 [53]. Type species: *Alaodima grandis* Dolin, 1980: 76 [53]. For more information, see Kundrata et al. [12].

Remark. We keep this genus tentatively under Dimini although Schimmel [91] and Schimmel and Tarnawski [131] placed it to Elaterinae without any explanation. Some characters of *A. grandis*, e.g., large body size, pointed last ventrite, attenuate elytral apices, transverse scutellar shield, are more typical for species of Oxynopterini rather than Dimini, though *A. grandis* differs from Oxynopterini in the structure of metacoxal plate and the shape of prosternal process. Unfortunately, this fossil lacks head and legs so many crucial diagnostic characters are absent.

 

***Alaodima grandis* Dolin, 1980**

*Alaodima grandis* Dolin, 1980: 76 [53].

Type material. Holotype, sex unknown, exoskeleton, compression fossil, No. 2066/2970 (part + counterpart) (PIN).

Fossil deposit/age. Kazakhstan: Karabastau Formation, Karatau, Mikhailovka; 166.1–157.3 Ma (Jurassic).

Literature. Dolin (1980: 76): original description [53]; Carpenter (1992: 304): generic catalogue [68]; Korneev and Cate (2005: 9): checklist [120]; Schimmel (2005: 28): remark, photo [91]; Schimmel and Tarnawski (2010: 363): remark [131]; Kundrata et al. (2018: 69): catalogue [160]; Kundrata et al. (2020: 7): generic catalogue [12]; Kundrata et al. (2020: 8): remark [94].

#### 3.3.3. Tribe Hypnoidini Schwarz, 1906 *

Hypnoidini Schwarz, 1906: 150 [161]. Type genus: *Hypnoidus* Dillwyn, 1829: 32 [162]. For more information, including synonyms, see Bouchard et al. [110].

 

- Genus *Ligmargus* Stibick, 1976 *

*Ligmargus* Stibick, 1976: 210 [163]. Type species: *Cryptohypnus funebris* Candèze, 1860: 62 [152]. For more information, see Cate [107].

 

***Ligmargus terrestris* (Scudder, 1879)**

*Cryptohypnus terrestris* Scudder, 1879: 180 [20].

*Ligmargus terrestris*: Stibick, 1981: 247 [164].

Type material Holotype, sex unknown, compression fossil, No. 59 (GSC).

Fossil deposit/age. Canada: British Columbia, Princeton Group, Nicola river; 56.0–47.8 Ma (Eocene).

Literature. Scudder (1879: 180): original description [20]; Scudder (1890: 497): catalogue [165]; Scudder (1891: 503): catalogue [24]; Scudder (1895: 38): catalogue [156]; Scudder (1900: 96): catalogue [157]; Handlirsch (1907: 745): catalogue [127]; Wickham (1920: 354): catalogue [29]; Stibick (1981: 247): revision [164].

 

#### 3.3.4. Tribe Oxynopterini Candèze, 1857 *

Oxynopterini Candèze, 1857: 355 [116]. Type genus: *Oxynopterus* Hope, 1842: 77 [166]. For more information, including synonyms, see Bouchard et al. [110].

 

- Genus *Campsosternus* Latreille, 1834 *

*Campsosternus* Latreille, 1834: 141 [167]. Type species: *Elater fulgens* Olivier, 1790: 12 [168] (syn. of *Elater auratus* Drury, 1773: 65 [169]). For more information, see Cate [107].

 

***Campsosternus atavus* Deichmüller, 1881**

*Campsosternus atavus* Deichmüller, 1881: 306 [21].

Type material. Unknown number of type specimens, probably only one, sex unknown, compression fossil (type depository unknown).

Fossil deposit/age. Czech Republic: Kučlín (u Bíliny); 37.2–33.9 Ma (Eocene).

Literature. Deichmüller (1881: 306): original description [21]; Scudder (1891: 483): catalogue [24]; Handlirsch (1907: 743): catalogue [127].

 

- Genus *Melanactes* LeConte, 1853 *

*Melanactes* LeConte, 1853: 493 [144]. Type species. *Melanactes densus* LeConte, 1853: 494 [144]. For more information, see Mathieu [141].

 

***Melanactes cockerelli* Wickham, 1908**

*Melanactes cockerelli* Wickham, 1908: 77 [27].

Type material. Holotype, sex unknown, exoskeleton, compression fossil, No. 3 (YPM).

Fossil deposit/age. USA: Colorado, Florissant Formation, Florissant, Station 14; 37.2–33.9 Ma (Eocene).

Literature. Wickham (1908: 77): original description [27]; Wickham (1916: 527): catalogue [28]; Wickham (1920: 354): catalogue [29].

#### 3.3.5. Tribe Prosternini Gistel, 1856 *

Prosternidae Gistel, 1856: 367 [170]. Type genus: *Prosternon* Latreille, 1834: 151 [167]. This tribe includes also Ctenicerini Jakobson, 1913 and Corymbitini LeConte, 1861, which both have been currently synonyms of Prosternini. For more information, see Cate [107] and Bouchard et al. [110].

 

- Genus *Ctenicera* Latreille, 1829 *

*Ctenicera* Latreille, 1829: 454 [118]. Type species: *Elater pectinicornis* Linnaeus, 1758: 406 [115]. This genus includes also species earlier attributed to genera *Ludius* Eschscholtz, 1829 (*nec* Berthold [134], *nec* Latreille [167]) and *Corymbites* Latreille, 1834, which are currently both synonyms of *Ctenicera*. For more information, see Hyslop [111] and Cate [107].

Remark. The generic assignment of all species classified under *Ctenicera* needs serious re-examination. Many of them probably belong to Selatosomini.

 

***Ctenicera emblemoelytra* (Zhang, 1989), comb. nov.**

*Corymbites emblemoelytrus* Zhang, 1989: 125 [58].

Type material. Holotype, sex unknown, exoskeleton, compression fossil, 750115/750116 (part + counterpart) (SMJS).

Fossil deposit/age. China: Shanwang Formation, Linqu County; 20.44–15.97 Ma (Miocene).

Literature. Zhang (1989: 125): original description [58]; Dong and Huang (2011: 1225): checklist [81].

Remark. This species was described in genus *Corymbites*, which is, however, a synonym of *Ctenicera* [107,111]. Generic attribution of this species remains unclear. Since it was compared with a recent species of *Pristilophus* Latreille, 1834 and with fossil *C. primitiva* (Wickham, 1908) [58], which might in fact belong to *Selatosomus* Stephens, 1830, affinities of this species to the tribe Selatosomini should be taken into consideration.

 

***Ctenicera euprepes* (Zhang, Sun and Zhang, 1994), comb. nov.**

*Corymbites euprepes* Zhang, Sun and Zhang, 1994: 92 [59].

Type material. Holotype, sex unknown, compression fossil, K0253 (SGMS).

Fossil deposit/age. China: Shanwang Formation, Linqu County; 20.44–15.97 Ma (Miocene).

Literature. Zhang et al. (1994: 92): original description [59]; Dong and Huang (2011: 1225): checklist [81].

Remark. This species was described in genus *Corymbites*, which is, however, a synonym of *Ctenicera* [107,111]. Its generic placement is unclear, since it superficially resembles the representatives of tribe Selatosomini.

 

***Ctenicera granulicollis* (Wickham, 1908), comb. nov.**

*Corymbites granulicollis* Wickham, 1908: 76 [27].

*Ludius granulicollis*: Wickham, 1920: 354 [29].

Type material. Holotype, sex unknown, exoskeleton, compression fossil, No. 1 (YPM).

Fossil deposit/age. USA: Colorado, Florissant Formation, Florissant Station 14; 37.2–33.9 Ma (Eocene).

Literature. Wickham (1908: 76): original description [27]; Wickham (1916: 524): catalogue [28]; Wickham (1920: 354): catalogue [29]; Zhang et al. (1994: 93): remark [59].

Remark. This species was described in genus *Corymbites* and later transferred to *Ludius* Eschscholtz, 1829 (*nec* Berthold [134], *nec* Latreille [167]) [29] which are currently both synonyms of *Ctenicera* [107,111]. This species most probably belongs to genus *Selatosomus* based on the body proportions and shapes of prothorax and elytra. However, we prefer to postpone any taxonomic changes pending a detailed examination of the type material.

 

***Ctenicera primitiva* (Wickham, 1908), comb. nov.**

*Corymbites primitivus* Wickham, 1908: 77 [27].

*Ludius primitivus*: Wickham, 1920: 354 [29].

Type material. Holotype, sex unknown, exoskeleton, compression fossil, No. 2 (YPM).

Fossil deposit/age. USA: Colorado, Florissant Formation, Florissant Station 13; 37.2–33.9 Ma (Eocene).

Literature. Wickham (1908: 77): original description [27]; Wickham (1916: 524): catalogue [28]; Wickham (1920: 354): catalogue [29]; Zhang (1989: 126): remark [58].

Remark. This species was described in genus *Corymbites* and later transferred to *Ludius* Eschscholtz, 1829 (*nec* Berthold [134], *nec* Latreille [167]) [29] which are currently both synonyms of *Ctenicera* [107,111]. This species most probably belongs to genus *Selatosomus* based on the body proportions and shapes of prothorax and elytra. However, we prefer to postpone any taxonomic changes pending a detailed examination of the type material.

 

***Ctenicera prophetica* (Wickham, 1916), comb. nov.**

*Corymbites propheticus* Wickham, 1916: 526 [28].

*Ludius propheticus*: Wickham, 1920: 354 [29].

Type material. Holotype, sex unknown, exoskeleton, compression fossil, MCZ 2724 (=13,657 in Scudder coll.) (MCZ).

Fossil deposit/age. USA: Colorado, Florissant Formation, Florissant; 37.2–33.9 Ma (Eocene).

Literature. Wickham (1916: 526): original description [28]; Wickham (1920: 354): catalogue [29].

Remark. This species was described in genus *Corymbites* and later transferred to *Ludius* Eschscholtz, 1829 (*nec* Berthold [134], *nec* Latreille [167]) [29] which are currently both synonyms of *Ctenicera* [107,111]. This species superficially resembles Selatosomini but its placement remains unclear.

 

***Ctenicera restructa* (Wickham, 1916), comb. nov.**

*Corymbites restructus* Wickham, 1916: 525 [28].

*Ludius restructus*: Wickham, 1920: 354 [29].

Type material. Holotype, sex unknown, compression fossil, No. 8215 (CUB).

Fossil deposit. USA: Colorado, Florissant Formation, Florissant, Station 14; 37.2–33.9 Ma (Eocene).

Literature. Wickham (1916: 525): original description [28]; Wickham (1920: 354): catalogue [29].

Remark. This species was described in genus *Corymbites* and later transferred to *Ludius* Eschscholtz, 1829 (*nec* Berthold [134], *nec* Latreille [167]) [29] which are currently both synonyms of *Ctenicera* [107,111]. This species superficially resembles Selatosomini but its placement remains unclear.

 

***Ctenicera sincera* (Zhang, Sun and Zhang, 1994), comb. nov.**

*Corymbites sincerus* Zhang, Sun and Zhang, 1994: 92 [59].

Type material. Holotype, sex unknown, compression fossil, SK000434 (Shanwang Fossil Protection Post collection; information taken from the Paleobiology Database, https://paleobiodb.org, accessed on 10 October 2020. May be “Shanwang Palaeontological Museum” in Linqu County (L. Qiu, personal communication). We have not been able to confirm depository information).

Fossil deposit/age. China: Shanwang Formation, Linqu County; 20.44–15.97 Ma (Miocene).

Literature. Zhang et al. (1994: 92): original description [59]; Dong and Huang (2011: 1225): checklist [81].

Remark. This species was described in genus *Corymbites*, which is currently a synonym of *Ctenicera* [107,111]. Generic attribution of this species remains unclear.

 

***Ctenicera submersa* (Wickham, 1916), comb. nov.**

*Corymbites submersus* Wickham, 1916: 524 [28].

*Ludius submersus*: Wickham, 1920: 354 [29].

Type material. Holotype, sex unknown, compression fossil, No. 8216 (CUB).

Fossil deposit/age. USA: Colorado, Florissant Formation, Florissant, Station 14; 37.2–33.9 Ma (Eocene).

Literature. Wickham (1916: 524): original description [28]; Wickham (1920: 354): catalogue [29].

Remark. This species was described in genus *Corymbites* and later transferred to *Ludius* Eschscholtz, 1829 (*nec* Berthold [134], *nec* Latreille [167]) [29] which are currently both synonyms of *Ctenicera* [107,111]. This species superficially resembles Oxynopterini but its placement remains unclear.

 

***Ctenicera sutor* (Heer, 1847), comb. nov.**

*Diacanthus sutor* Heer, 1847: 136 [14].

*Corymbites sutor*: Heer, 1861: 204 [171].

Type material. Two syntypes, sex unknown, compression fossils (SMNK, ETH (No. 7911)).

Fossil deposit/age. Germany: Upper Freshwater-Molasse Formation, Öhningen, MN 7 mammal zone; 12.7–11.608 Ma (Miocene).

Literature. Heer (1847: 136): original description [14]; Giebel (1852: 651): catalogue [126]; Giebel (1856: 95): revision, redescription [16]; Heer (1861: 204): catalogue [171]; Scudder (1891: 508): catalogue [24]; Handlirsch (1907: 746): catalogue [127]; Theobald (1937: 175): remark [42].

Remark. This species was classified in genus *Corymbites* [171] which is currently a synonym of *Ctenicera* [107,111]. This species is superficially similar to some species of *Limonius* but its placement remains unclear.

 

***Ctenicera velata* (Scudder, 1876), comb. nov.**

*Corymbites velatus* Scudder, 1876: 81 [19].

*Ludius velatus*: Wickham, 1920: 354 [29].

Type material. Holotype, sex unknown, compression fossil, No. 3458 (MCZ).

Fossil deposit/age. USA: Wyoming, Green River Formation, Laney Member, Petrified fish cut; 50.3–46.2 Ma (Eocene).

Literature. Scudder (1876: 81): original description [19]; Scudder (1878: 762): catalogue [172]; Scudder (1890: 496): catalogue [165]; Scudder (1891: 501): catalogue [24]; Scudder (1900: 96): catalogue [157]; Handlirsch (1907: 746): catalogue [127]; Wickham (1920: 354): catalogue [29].

Remark. This species was described in genus *Corymbites* and later transferred to *Ludius* Eschscholtz, 1829 (*nec* Berthold [134], *nec* Latreille [167]) [29] which are currently both synonyms of *Ctenicera* [107,111]. The generic attribution of this species is unclear since it was described based almost exclusively on elytral characters.

 

- **Genus** *Eanus* LeConte, 1861 *

*Eanus* LeConte, 1861: 171 [173]. Type species: *Limonius estriatus* LeConte, 1853: 434 [144]. This genus includes also species earlier attributed to genus *Paranomus* Kiesenwetter, 1858 (earlier also as part of *Ludius* Eschscholtz, 1829, *nec* Berthold [134], *nec* Latreille [167]), which is currently a synonym of *Eanus*. For more information, see Hyslop [111], Johnson [106] and Cate [107].

Remark. All fossil species listed under this genus should be re-examined as their body proportions and structure of prothorax resemble more Negastriinae or Cardiophorinae rather than *Eanus*. Current representatives of *Eanus* have more elongated elytra and more or less campaniform pronotum, while in the here listed fossil species, elytra are relatively shorter and the pronotum is more or less rounded and sinuate near posterior angles. The shapes of scutellar shields in drawings by Wickham [28] also suggest similarity with Negastriinae.

 

***Eanus exanimatus* (Wickham, 1916), comb. nov.**

*Paranomus exanimatus* Wickham, 1916: 520 [28].

*Ludius exanimatus*: Wickham, 1920: 354 [29].

Type material. Holotype, sex unknown, compression fossil, No. 90,496 (USNM).

Fossil deposit/age. USA: Colorado, Florissant Formation, Wilson Ranch, Florissant; 37.2–33.9 Ma (Eocene).

Literature. Wickham (1916: 520): original description [28]; Wickham (1920: 354): catalogue [29].

Remark. This species was described in genus *Paranomus* (earlier also as part of *Ludius* Eschscholtz, 1829, *nec* Berthold [134], *nec* Latreille [167]), which is currently a synonym of *Eanus*.

 

***Eanus heeri* (Wickham, 1916), comb. nov.**

*Paranomus heeri* Wickham, 1916: 521 [28].

*Ludius heeri*: Wickham, 1920: 354 [29].

Type material. Holotype, sex unknown, compression fossil (?CUB).

Fossil deposit/age. USA: Colorado: Florissant Formation, Florissant, Station 14; 37.2–33.9 Ma (Eocene).

Literature. Wickham (1916: 521): original description [28]; Wickham (1920: 354): catalogue [29].

Remark. This species was described in genus *Paranomus* (later also as part of *Ludius* Eschscholtz, 1829, *nec* Berthold [134], *nec* Latreille [167]), which is currently a synonym of *Eanus*. Its generic placement is unclear, and already Wickham [28] mentioned that this was probably not true *Paranomus* (now *Eanus*).

 

***Eanus laevissimus* (Wickham, 1916), comb. nov.**

*Paranomus laevissimus* Wickham, 1916: 521 [28].

*Ludius laevissimus*: Wickham, 1920: 354 [29].

Type material. Holotype, sex unknown, compression fossil (?CUB).

Fossil deposit/age. USA: Colorado, Florissant Formation, Florissant, Station 14; 37.2–33.9 Ma (Eocene).

Literature. Wickham (1916: 521): original description [28]; Wickham (1920: 354): catalogue [29].

Remark. This species was described in genus *Paranomus* (earlier also as part of *Ludius* Eschscholtz, 1829, *nec* Berthold [134], *nec* Latreille [167]), which is currently a synonym of *Eanus*.

 

- Genus *Oxygonus* LeConte, 1863 *

*Oxygonus* LeConte, 1863: 48 [174]. Type species: *Elater obesus* Say, 1823: 168 [175]. For more information, see Hyslop [111] and Johnson [106].

 

***Oxygonus mortuus* Scudder, 1876**

*Oxygonus mortuus* Scudder, 1876: 81 [19].

Type material. Holotype, sex unknown, elytron, compression fossil (type depository has not been identified).

Fossil deposit/age. USA: Utah, Green River Formation, Fossil Cañon; 50.3–46.2 Ma (Eocene).

Literature. Scudder (1876: 81): original description [19]; Scudder (1877: 759): catalogue [176]; Scudder (1890: 496): catalogue [165]; Scudder (1891: 562): catalogue [24]; Scudder (1900: 97): catalogue [157]; Handlirsch (1907: 747): catalogue [127]; Wickham (1920: 354): catalogue [29].

Remark. The generic attribution of this species is unclear as it was described based only on elytral characters.

 

***Oxygonus primus* Wickham, 1916**

*Oxygonus primus* Wickham, 1916: 526 [28].

**Figure 1:**
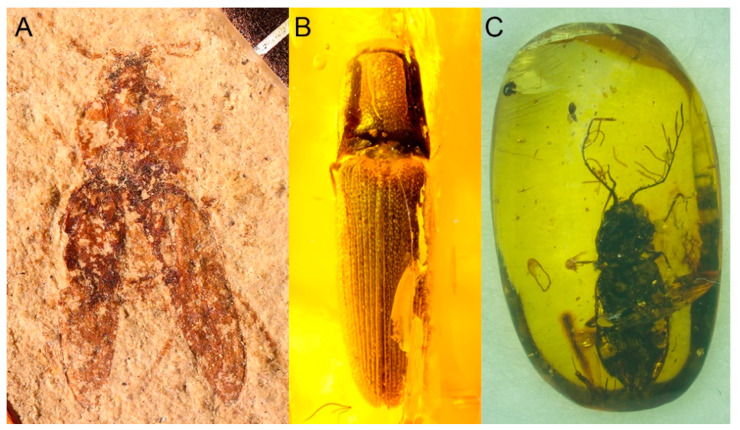
Type specimens of fossil Elateridae. (**A**) *Oxygonus primus* Wickham, 1916, holotype (MCZ), Florissant, Colorado, USA, body length: 6.0 mm (credit: MCZ—H. Meyer, M. Aja); (**B**) *Megapenthes voigti* Schimmel, 2005, paratype (BMNH), North European Baltic amber, body length: 5.9 mm (credit: BMNH—K. Matsumoto); (**C**) *Cretopityobius pankowskiorum* Otto, 2019, paratype (WIRC), Burmese amber, Myanmar, body length: 6.5 mm.

Type material. Holotype, sex unknown, exoskeleton, compression fossil, No. 4069 (=6381 in Scudder coll.) (MCZ) (Figure 1A).

Fossil deposit/age. USA: Colorado, Florissant Formation, Florissant; 37.2–33.9 Ma (Eocene).

Literature. Wickham (1916: 526): original description [28]; Wickham (1920: 354): catalogue [29].

Remark. This species most probably does not belong to *Oxygonus* as it differs considerably in the shape of prothorax (almost globular versus more or less elongate, with arcuate sides in *Oxygonus*) [177].

#### 3.3.6. Tribe Selatosomini Schimmel, Tarnawski, Han and Platia, 2015 *

Selatosomini Schimmel, Tarnawski, Han and Platia, 2015: 30 [178]. Type genus: *Selatosomus* Stephens, 1830: 268 [179]. For more information, see Schimmel et al. [178].

 

- Genus *Selatosomus* Stephens, 1830 *

*Selatosomus* Stephens, 1830: 268 [179]. Type species: *Elater aeneus* Linnaeus, 1758: 406 [115]. For more information, see Cate [107].

 

***Selatosomus miegi* Theobald, 1937**

*Selatosomus miegi* Theobald, 1937: 175 [42].

Type material. Holotype, sex unknown, elytron, compression fossil, R 624 (NHMB).

Fossil deposit/age. Germany: Middle Member (Salt Formation), Kleinkembs; 33.9–28.4 Ma (Oligocene).

Literature. Theobald (1937: 175): original description [42].

Remark. The generic attribution of this species is unclear as this was described based only on elytral characters.

 

#### 3.3.7. Tribe Semiotini Jakobson, 1913 *

Semiotina Jakobson, 1913: 736 [180]. Type genus: *Semiotus* Eschscholtz, 1829: 31 [117]. For more information, see Bouchard et al. [110].

 

- Genus *Semiotus* Eschscholtz 1829 *

*Semiotus* Eschscholtz 1829: 31 [117]. Type species: *Elater furcatus* Fabricius, 1775: 224 [181]. For more information, see Hyslop [111].

 

***Semiotus ehrenswaerdi* (Heer, 1870)**

*Elater ehrenswaerdi* Heer, 1870: 74 [18].

*Elater ehrenwaerdi*: Scudder, 1891: 517 [24] [unavailable name, incorrect subsequent spelling not in prevailing usage; [129], Art. 33.3].

*Elater ehrenwärdi* [sic!]: Handlirsch, 1907: 745 [127].

*Semiotus ehrensvaerdi*: Birket-Smith, 1977: 18 [154] [unavailable name, incorrect subsequent spelling not in prevailing usage; [129], Art. 33.3].

Type material. Lectotype, sex unknown, elytron, compression fossil, No. 54a,b (SMNH). Paralectotype, sex unknown, elytron, compression fossil, No. 54c (SMNH).

Fossil deposit/age. Norway: Svalbard and Jan Mayen, Firkanten Formation, Cap Staratschin; 66.0–59.2 Ma (Paleocene).

Literature. Heer (1870: 74): original description [18]; Scudder (1891: 517): catalogue [24]; Handlirsch (1907: 745): catalogue [127]; Birket-Smith (1977: 18): taxonomic revision [154].

Remark. The description is based on a part of isolated elytron so the generic attribution of this species is rather problematic. However, based on the elytron reconstruction by Birket-Smith [154], it is really similar to that of *Semiotus*.

 

***Semiotus menatensis* Piton, 1940**

*Semiotus menatensis* Piton, 1940: 179 [43].

Type material. Holotype, sex unknown, exoskeleton, compression fossil, No. 943 (MNHN).

Fossil deposit/age. France: Menat Formation, Menat; 61.6–59.2 Ma (Paleocene).

Literature. Piton (1940: 179): original description [43].

Remark. Based on the body proportions, shape of antenna and rather broad and short pronotum, this species strongly resembles recent *Campsosternus* (Oxynopterini). However, we prefer to postpone any taxonomic changes pending a comprehensive review including the type material.

### 3.4. Subfamily Elaterinae Leach, 1815 *

*Elaterides* Leach, 1815: 85 [114]. Type genus: *Elater* Linnaeus, 1758: 404 [115]. For more information including synonyms, see Bouchard et al. [110].

#### 3.4.1. Tribe Agriotini Laporte, 1840 *

Agriotites Laporte, 1840: 233 [182]. Type genus: *Agriotes* Eschscholtz, 1829: 34 [117]. For more information, see Cate [107] and Bouchard et al. [110].

 

- Genus *Agriotes* Eschscholtz, 1829 *

*Agriotes* Eschscholtz, 1829: 34 [117]. Type secies: *Elater sputator* Linnaeus, 1758: 405 [115]. For more information, see Cate [107].

 

***Agriotes comminutus* Wickham, 1916**

*Agriotes comminutus* Wickham, 1916: 513 [28].

Type material. Holotype, sex unknown, exoskeleton, compression fossil, MCZ 2747 (= 11,800 in Scudder coll.) (MCZ).

Fossil deposit/age. USA: Colorado, Florissant Formation, Florissant; 37.2–33.9 Ma (Eocene).

Literature. Wickham (1916: 513): original description [28]; Wickham (1920: 354): catalogue [29].

 

***Agriotes nearcticus* Wickham, 1916**

*Agriotes nearcticus* Wickham, 1916: 513 [28].

Type material. Holotype, sex unknown, exoskeleton, compression fossil, MCZ 2748 (=6653 in Scudder coll.) (MCZ).

Fossil deposit/age. USA: Colorado, Florissant Formation, Florissant; 37.2–33.9 Ma (Eocene).

Literature. Wickham (1916: 513): original description [28]; Wickham (1920: 354): catalogue [29].

 

***Agriotes succiniferus*** **Becker, 1963**

*Agriotes succiniferus* Becker, 1963: 127 [46].

*Agriotes succinifer*: Zaragoza Caballero, 1990: 147 [61] [unavailable name, incorrect subsequent spelling not in prevailing usage; [129], Art. 33.3].

Type material. Holotype, female, exoskeleton, amber inclusion, No. 12,972 (UCMP).

Fossil deposit/age. Mexico: Simojovel region, Mexican (Chiapas) amber; 23.03–15.97 Ma (Miocene).

Literature. Becker (1963: 127): original description [46]; Spahr (1981: 46): catalogue [49]; Keilbach (1982: 247): catalogue [133]; Zaragoza Caballero (1990: 147): remark [61]; Schimmel (2005: 27): remark [91]; Solórzano Kraemer (2007: 119): checklist [90]; Schimmel and Tarnawski (2010: 363): remark [131]; Schimmel and Tarnawski (2012: 265): remark [132].

#### 3.4.2. Tribe Ampedini Gistel, 1848 *

Ampedidae Gistel, 1848: 5 [138]. Type genus: *Ampedus* Dejean, 1833: 92 [139]. For more information, see Bouchard et al. [110].

 

- Genus *Ampedus* Dejean, 1833 *

*Ampedus* Dejean, 1833: 92 [139]. Type species: *Elater sanguineus* Linnaeus, 1758: 405 [115]. For more information see Sánchez-Ruiz [121] and Cate [107].

 

**Subgenus *Octamenogonoides* Iablokoff-Khnzorian, 1961**

*Octamenogonoides* Iablokoff-Khnzorian, 1961: 88 [47]. Type species: *Elater* (*Octamenogonoides*) *gebleri* Iablokoff-Khnzorian, 1961: 88 [47]. For more information, see Alekseev [92] and Kundrata et al. [12].

Remark. Iablokoff-Khnzorian [47] described *Octamenogonoides* as a subgenus in *Elater* Linnaeus, 1758, and Schimmel and Tarnawski [131] treated it is a member of the tribe Elaterini. Alekseev [92] transferred *Octamenogonoides* to *Ampedus* and kept its subgeneric status.

 

***Ampedus (Octamenogonoides) gebleri* (Iablokoff-Khnzorian, 1961)**

*Elater (Octamenogonoides) gebleri* Iablokoff-Khnzorian, 1961: 88 [47].

*Elater gebleri*: Larsson, 1978: 153 [48].

Ampedus (Octamenogonoides) gebleri: Alekseev, 2013: 7 [92].

Type material. Holotype, sex unknown, exoskeleton, amber inclusion, No. 364/641 (PIN).

Fossil deposit/age. Baltic amber; 38.0–33.9 Ma (Eocene).

Literature. Iablokoff-Khnzorian (1961: 88): original description [47]; Larsson (1978: 153): catalogue [48]; Spahr (1981: 48): catalogue [49]; Keilbach (1982: 246): catalogue [133]; Schimmel (2005: 27): remark [91]; Schimmel and Tarnawski (2010: 364): remark [131]; Alekseev (2013: 7): checklist [92]; Kundrata et al. (2020: 8): generic catalogue [12].

 

**Subgenus *Ampedus* Dejean, 1833**

*Ampedus* Dejean, 1833: 92 [139]. Type species: *Elater sanguineus* Linnaeus, 1758: 405 [115]. For more information see Sánchez-Ruiz [121] and Cate [107].

 

***Ampedus seyfriedii* Heer, 1847**

*Ampedus seyfriedii* Heer, 1847: 131 [14].

*Ampedus seyfriedi*: Giebel, 1852: 651 [126] [unavailable name, incorrect subsequent spelling not in prevailing usage; [129], Art. 33.4].

Type material. Holotype, sex unknown, compression fossil (ETH).

Fossil deposit/age. Germany: Upper Freshwater-Molasse Formation, Öhningen, MN-7 mammal zone, Upper Öhningen beds Member; 12.7–11.608 Ma (Miocene).

Literature. Heer (1847: 131): original description [14]; Giebel (1852: 651): catalogue [126]; Giebel (1856: 97): redescription [16]; Heer (1861: 204): catalogue [171]; Heer (1865: 362): remark [17]; Heer (1872: 444): remark [135]; Heer (1876: 16): remark [136]; Handlirsch (1907: 743): catalogue [127].

 

- **Genus** *Ischnodes* Germar, 1844 *

*Ischnodes* Germar, 1844: 180 [183]. Type species: *Elater sanguinicollis* Panzer, 1793: 13 [184]. For more information, see Cate [107].

 

***Ischnodes gracilis* Heer, 1847**

*Ischnodes gracilis* Heer, 1847: 133 [14].

Type material. Holotype, sex unknown, compression fossil, No. 7908 (ETH).

Fossil deposit/age. Germany: Upper Freshwater-Molasse Formation, Öhningen, Upper Öhningen beds; 12.7–11.608 Ma (Miocene).

Literature. Heer (1847: 133): original description [14]; Giebel (1852: 651): catalogue [126]; Giebel (1856: 97): redescription [16]; Scudder (1891: 542): catalogue [24]; Handlirsch (1907: 745): catalogue [127].

#### 3.4.3. Tribe Elaterini Leach, 1815 *

Elaterides Leach, 1815: 85 [114]. Type genus: *Elater* Linnaeus, 1758: 404 [115]. For more information, including synonyms, see Bouchard et al. [110].

 

- **Genus *Diaraphes* Iablokoff-Khnzorian, 1961**

*Diaraphes* Iablokoff-Khnzorian, 1961: 89 [47]. Type species: *Diaraphes kozhantshikovi* Iablokoff-Khnzorian, 1961: 89 [47]. For more information, see Kundrata et al. [12].

 

***Diaraphes kozhantshikovi* Iablokoff-Khnzorian, 1961**

*Diaraphes kozhantshikovi* Iablokoff-Khnzorian, 1961: 89 [47].

Type material. Holotype, sex unknown, exoskeleton, amber inclusion, No. 364/645 (PIN).

Fossil deposit/age. Baltic amber; 38.0–33.9 Ma (Eocene).

Literature. Iablokoff-Khnzorian (1961: 89): original description [47]; Larsson (1978: 153): catalogue [48]; Spahr (1981: 47): catalogue [49]; Keilbach (1982: 246): catalogue [133]; Carpenter (1992: 304): generic catalogue [68]; Schimmel (2005: 27): remark [91]; Alekseev (2013: 7): checklist [92]; Kundrata et al. (2020: 8): generic catalogue [12].

 

- Genus *Elater* Linnaeus, 1758 *

*Elater* Linnaeus, 1758: 404 [115]. Type species: *Elater ferrugineus* Linnaeus, 1758: 405 [115]. For more information, see Cate [107].

Remark. Fossil species assigned to this genus urgently need a revision. It is possible that most, if not all, species in fact belong to another click-beetle genera. It should be also noted, that many older authors used the name “*Elater*” in erroneuous way (nec *Elater* Linnaeus [115]), i.e., for *Ampedus* (for more information, see Hyslop [111]), so they actually compared these fossil species with *Ampedus* and not with *Elater*.

 

***Elater asmodeus* Zhang, 1989**

*Elater asmodeus* Zhang, 1989: 123 [58].

Type material. Holotype, sex unknown, exoskeleton, compression fossil, No. s82747 (SMJS).

Fossil deposit/age. China: Shanwang Formation, Shanwang, Linqu County; 20.44–15.97 Ma (Miocene).

Literature. Zhang (1989: 123): original description [58]; Dong and Huang (2011: 1225): checklist [81].

Remark. This species superficially (i.e., in the body proportions and the structure of thorax) resembles Dendrometrinae rather than Elaterinae.

 

***Elater berryi* Wickham, 1929**

*Elater berryi* Wickham, 1929: 148 [41].

Type material. Holotype, sex unknown, elytron, compression fossil, No. 80,474 (USNM).

Fossil deposit/age. USA: Tennessee, Cockfield Formation, 4 miles north of Jackson, Madison County; 41.3–38.0 Ma (Eocene).

Literature. Wickham (1929: 148): original description [41]; Wickham (1933: 103): catalogue [185].

 

***Elater burmitinus* Cockerell, 1917**

*Elater burmitinus* Cockerell, 1917: 325 [33].

Type material. Holotype, sex unknown, exoskeleton, amber inclusion, No. 19,102 (BMNH).

Fossil deposit/age. Myanmar: Burmese amber; 99.6–93.5 Ma (Cretaceous).

Literature. Cockerell (1917: 325): original description [33]; Fletcher (1920: 987): remark [186]; Zherikhin (1978: 114): remark [187]; Spahr (1981: 47): catalogue [49]; Keilbach (1982: 247): checklist [133]; Poinar (1992: 144): remark [188]; Ross and York (2000: 12): catalogue [189]; Peris and Háva (2016: 496): remark [190].

Remark. Cockerell [33] suggested that it is not a member of true *Elater*; however, he was not able to assign it to any other genus. It was listed as “Elateridae sens. l. *burmitinus*” by Keilbach [133]. The original description and available figure are not enough to make any conclusions about the placement of this species, and the proper study of the type specimen should be carried out in order to exclude the possibility that it is a member of Eucnemidae.

 

***Elater canabinus* Zhang, 1989**

*Elater canabinus* Zhang, 1989: 124 [58].

Type material. Holotype, sex unknown, exoskeleton, compression fossil, No. 840,105 (SMJS).

Fossil deposit/age. China: Shanwang Formation, Shanwang, Linqu County; 20.44–15.97 Ma (Miocene).

Literature. Zhang (1989: 124): original description [58]; Dong and Huang (2011: 1225): checklist [81].

 

***Elater florissantensis* Wickham, 1916**

*Elater florissantensis* Wickham, 1916: 510 [28].

Type material. Holotype, sex unknown, exoskeleton, compression fossil, MCZ 2752 (=8034 in Scudder coll.) (MCZ).

Fossil deposit/age. USA: Colorado, Florissant Formation, Florissant; 37.2–33.9 Ma (Eocene).

Literature. Wickham (1916: 510): original description [28]; Wickham (1920: 354): catalogue [29].

 

***Elater mitrus* Zhang, 1989**

*Elater mitrus* Zhang, 1989: 122 [58].

Type material. Holotype, sex unknown, exoskeleton, compression fossil, No. s82713 (SMJS).

Fossil deposit/age. China: Shanwang Formation, Shanwang, Linqu County; 20.44–15.97 Ma (Miocene).

Literature. Zhang (1989: 122): original description [58]; Dong and Huang (2011: 1225): checklist [81].

Remark. This species is similar to Ampedini in the body proportions, the narrowed campaniform pronotum, the elongate, almost parallel-sided elytra and the slightly serrated antenna. In Elaterini, the pronotum is usually wider, elytra not parallel sided, often somewhat wedge-shaped, and antenna more serrated. However, we prefer to keep this species tentatively in *Elater* until the holotype is examined in detail.

 

***Elater naumanni* Giebel, 1856**

*Elater naumanni* Giebel, 1856: 91 [16].

Type material. Holotype, sex unknown, amber inclusion (Leipzig University collection).

Fossil deposit/age. Baltic amber; 38.0–33.9 Ma (Eocene).

Literature. Giebel (1856: 91): original description [16]; Handlirsch (1907: 744): catalogue [127]; Larsson (1978: 153): catalogue [48]; Keilbach (1982: 246): catalogue [133]; Alekseev (2013: 7): checklist [92].

Remark. This species was considered by Larsson [48] more similar to *Limonius* than to *Elater*. Type material should be studied in order to confirm the placement of this species.

 

***Elater rohweri* Wickham, 1916**

*Elater rohweri* Wickham, 1916: 509 [28].

Type material. Holotype, sex unknown, compression fossil, No. 8227 (CUB).

Fossil deposit. USA: Colorado, Florissant Formation, Florissant, Station 14; 37.2–33.9 Ma (Eocene).

Literature. Wickham (1916: 509): original description [28]; Wickham (1920: 354): catalogue [29].

Remark. This species superficially resembles Ampedini in having slightly arcuate pronotum which is sinuate near posterior angles, and slightly elongated subparallel sided. However, we prefer to keep this species tentatively in *Elater* until the holotype is examined in detail.

 

***Elater scudderi* Wickham, 1916**

*Elater scudderi* Wickham, 1916: 510 [28].

Type material. Holotype, sex unknown, exoskeleton, compression fossil, MCZ 2751 (=12,485 in Scudder coll.) (MCZ).

Fossil deposit. USA: Colorado, Florissant Formation, Florissant; 37.2–33.9 Ma (Eocene).

Literature. Wickham (1916: 510): original description [28]; Wickham (1920: 354): catalogue [29].

Remark. Wickham [28] already mentioned that this species probably does not belong to true *Elater*. Indeed, it has notably arcuate pronotum, with small posterior angles which is not a character usually found in Elaterinae. The generic attribution of *E. scudderi* needs further investigation.

 

***Elater wisniowskii* Lomnicki, 1902**

*Elater wisniowskii* Lomnicki, 1902: 11 [25].

Type material. Holotype (probably), sex unknown, elytron, compression fossil (type depository unknown).

Fossil deposit/age. Ukraine: Bashkev Formation: Myszyn, Galicia; 13.65–12.7 Ma (Miocene).

Literature. Lomnicki (1902: 11): original description [25]; Handlirsch (1907: 745): catalogue [127].

Remark. The generic attribution of this species is unclear as it was described based only on elytral characters.

 

- **Genus *Elatron* Iablokoff-Khnzorian, 1961**

*Elatron* Iablokoff-Khnzorian, 1961: 90 [47]. Type species: *Elatron semenovi* Iablokoff-Khnzorian, 1961: 90 [47]. For more information, see Kundrata et al. [12].

 

***Elatron semenovi* Iablokoff-Khnzorian, 1961**

*Elatron semenovi* Iablokoff-Khnzorian, 1961: 90 [47].

Type material. Holotype, sex unknown, exoskeleton, amber inclusion, No. 364/650 (PIN).

Fossil deposit/age. Baltic amber; 38.0–33.9 Ma (Eocene).

Literature. Iablokoff-Khnzorian (1961: 90): original description [47]; Larsson (1978: 153): catalogue [48]; Spahr (1981: 48): catalogue [49]; Keilbach (1982: 246): catalogue [133]; Carpenter (1992: 304): generic catalogue [68]; Schimmel (2005: 27): remark [91]; Alekseev (2013: 7): checklist [92]; Kundrata et al. (2020: 8): generic catalogue [12].

 

- **Genus *Holopleurus* Iablokoff-Khnzorian, 1961**

*Holopleurus* Iablokoff-Khnzorian, 1961: 86 [47]. Type species: *Holopleurus succineus* Iablokoff-Khnzorian, 1961: 86 [47]. For more information, see Kundrata et al. [12].

 

***Holopleurus succineus* Iablokoff-Khnzorian, 1961**

*Holopleurus succineus* Iablokoff-Khnzorian, 1961: 86 [47].

Type material. Holotype, sex unknown, exoskeleton, amber inclusion, No. 364/530 (PIN).

Fossil deposit/age. Baltic amber; 38.0–33.9 Ma (Eocene).

Literature. Iablokoff-Khnzorian (1961: 86): original description [47]; Larsson (1978: 153): catalogue [48]; Spahr (1981: 48): catalogue [49]; Keilbach (1982: 246): catalogue [133]; Carpenter (1992: 304): generic catalogue [68]; Schimmel (2005: 27): remark [91]; Alekseev (2013: 7): checklist [92]; Kundrata et al. (2020: 8): generic catalogue [12].

 

- **Genus** ***Orthoraphes* Iablokoff-Khnzorian, 1961**

*Orthoraphes* Iablokoff-Khnzorian, 1961: 86 [47]. Type species: *Orthoraphes reichardti* Iablokoff-Khnzorian, 1961: 87 [47]. For more information, see Kundrata et al. [12].

 

***Orthoraphes reichardti* Iablokoff-Khnzorian, 1961**

*Orthoraphes reichardti* Iablokoff-Khnzorian, 1961: 87 [47].

Type material. Holotype, sex unknown, exoskeleton, amber inclusion, No. 364/469 (PIN).

Fossil deposit/age. Baltic amber; 38.0–33.9 Ma (Eocene).

Literature. Iablokoff-Khnzorian (1961: 87): original description [47]; Larsson (1978: 153): catalogue [48]; Spahr (1981: 49): catalogue [49]; Keilbach (1982: 246): catalogue [133]; Carpenter (1992: 305): generic catalogue [68]; Schimmel (2005: 27): remark [91]; Alekseev (2013: 7): checklist [92]; Kundrata et al. (2020: 8): generic catalogue [12].

#### 3.4.4. Tribe Megapenthini Gurjeva, 1973 *

Megapenthini Gurjeva, 1973: 448 [191]. Type genus: *Megapenthes* Kiesenwetter, 1858: 353 [192]. For more information, see Bouchard et al. [110].

 

- Genus *Abelater* Fleutiaux, 1947 *

*Abelater* Fleutiaux, 1947: 383 [193]. Type species: *Melanoxanthus rubiginosus* Candèze, 1878: 138 [194]. For more information, see Cate [107].

 

***Abelater succineus* Schimmel, 2005**

*Abelater succineus* Schimmel, 2005: 106 [91].

Type material. Holotype, female, exoskeleton, amber inclusion, No. 4462 (CGG 2450) (GPIUH).

Fossil deposit/age. Baltic Amber; 38.0–33.9 Ma (Eocene).

Literature. Schimmel (2005: 106): original description [91]; Schimmel and Tarnawski (2010: 363): remark [131]; Schimmel and Tarnawski (2012: 265): remark [132]; Alekseev (2013: 7): checklist [92].

 

- Genus *Megapenthes* Kiesenwetter, 1858 *

*Megapenthes* Kiesenwetter, 1858: 353 [192]. Type species: *Elater lugens* Redtenbacher, 1842: 11 [195]. For more information, see Cate [107].

 

***Megapenthes groehni* Schimmel, 2005**

*Megapenthes groehni* Schimmel, 2005: 107 [91].

Type material. Holotype, male, exoskeleton, amber inclusion, No. 4463 (CGG 1184) (GPIUH).

Fossil deposit/age. Baltic Amber; 38.0–33.9 Ma (Eocene).

Literature. Schimmel (2005: 107): original description [91]; Schimmel and Tarnawski (2010: 363): remark [131]; Schimmel and Tarnawski (2012: 265): remark [132]; Alekseev (2013: 7): checklist [92].

 

***Megapenthes primaevus* Wickham, 1916**

*Megapenthes primaevus* Wickham, 1916: 511 [28].

Type material. Holotype, sex unknown, exoskeleton, compression fossil, MCZ 2750 (=10,859 in Scudder coll.) (MCZ).

Fossil deposit/age. USA: Colorado, Florissant Formation, Florissant; 37.2–33.9 Ma (Eocene).

Literature. Wickham (1916: 511): original description [28]; Wickham (1920: 354): catalogue [29]; Alekseev (2013: 7): checklist [92].

 

***Megapenthes voigti* Schimmel, 2005**

*Megapenthes voigti* Schimmel, 2005: 110 [91].

Type material. Holotype, female, exoskeleton, amber inclusion, No. 4469 (CGG 4612) (GPIUH). Paratype, male, exoskeleton, amber inclusion, CGG 2389 (BMNH) (Figure 1B).

Fossil deposit/age. Baltic Amber; 38.0–33.9 Ma (Eocene).

Literature. Schimmel (2005: 110): original description [91]; Schimmel and Tarnawski (2010: 363): remark [131]; Schimmel and Tarnawski (2012: 265): remark [132]; Alekseev (2013: 7): checklist [92].

 

#### 3.4.5. Tribe Physorhinini Candèze, 1859 *

Physorhinites Candèze, 1859: 384 [148]. Type genus: *Physorhinus* Germar, 1840: 244 [196]. For more information, see Bouchard et al. [110].

 

- Genus *Anchastus* LeConte, 185 *

*Anchastus* LeConte, 1853: 459 [144]. Type species: *Anchastus digitatus* LeConte, 1853: 459 [144]. For more information, see Cate [107] and Johnson [106].

 

***Anchastus diluvialis* Wickham, 1916**

*Anchastus diluvialis* Wickham, 1916: 507 [28].

Type material. Holotype, sex unknown, exoskeleton, compression fossil, MCZ 2759 (=11,277 in Scudder coll.) (MCZ).

Fossil deposit/age. USA: Colorado, Florissant Formation, Florissant; 37.2–33.9 Ma (Eocene).

Literature. Wickham (1916: 506): original description [28]; Wickham (1920: 354): catalogue [29].

 

***Anchastus eruptus* Wickham, 1916**

*Anchastus eruptus* Wickham, 1916: 507 [28].

Type material. Holotype, sex unknown, exoskeleton, compression fossil, MCZ 2760 (=11,281 in Scudder coll.) (MCZ).

Fossil deposit/age. USA: Colorado, Florissant Formation, Florissant; 37.2–33.9 Ma (Eocene).

Literature. Wickham (1916: 506): original description [28]; Wickham (1920: 354): catalogue [29].

#### 3.4.6. Tribe Synaptini Gistel, 1856 *

Synaptidae Gistel, 1856: 366 [170]. Type genus: *Synaptus* Eschscholtz, 1829: 32 [117]. For more information, see Cate [107] and Bouchard et al. [110].

 

- **Genus** *Glyphonyx* Candèze, 1863 *

*Glyphonyx* Candèze, 1863: 451 [145]. Type species: *Glyphonyx gundlachii* Candèze, 1863: 453 [145]. For more information, see Hyslop [111] and Cate [107].

 

***Glyphonyx chiapasensis* Zaragoza Caballero, 1990**

*Glyphonyx chiapasensis* Zaragoza Caballero, 1990: 148 [61].

Type material. Holotype, probably male, amber inclusion (UNAM).

Fossil deposit/age. Mexico: Simojovel region, Mexican (Chiapas) amber; 23.03–15.97 Ma (Miocene).

Literature. Zaragoza Caballero (1990: 148): original description [61]; Solórzano Kraemer (2007: 119): catalogue [90].

Remark. The generic attribution of this species needs re-examination. Since this species has a very small body size and a rather unusual shape of body and prothorax (including a lack of the Synaptini-characteristic lateral basal indentations of pronotum), it might belong to another genus than *Glyphonyx*.

 

***Glyphonyx punctatus* Becker, 1963**

*Glyphonyx punctatus* Becker, 1963: 127 [46].

Type material. Holotype, female, exoskeleton, amber inclusion, Nr. 12,873 (UCMP).

Fossil deposit/age. Mexico: Simojovel region, Mexican (Chiapas) amber; 23.03–15.97 Ma (Miocene).

Literature. Becker (1963: 127): original description [46]; Spahr (1981: 48): catalogue [49]; Keilbach (1982: 247): catalogue [133]; Zaragoza Caballero (1990: 147): redescription [61]; Schimmel (2005: 27): remark [91]; Solórzano Kraemer (2007: 119): catalogue [90]; Schimmel and Tarnawski (2010: 363): remark [131]; Schimmel and Tarnawski (2012: 265): remark [132].

#### 3.4.7. Elaterinae Incertae Sedis

 

- **Genus** ***Crioraphes* Iablokoff-Khnzorian, 1961**

*Crioraphes* Iablokoff-Khnzorian, 1961: 93 [47]. Type species: *Crioraphes rohdendorfi* Iablokoff-Khnzorian, 1961: 94 [47]. For more information, see Douglas [4] and Kundrata et al. [12].

 

***Crioraphes rohdendorfi* Iablokoff-Khnzorian, 1961**

*Crioraphes rohdendorfi* Iablokoff-Khnzorian, 1961: 94 [47].

*Crioraphes rhodendorfi*: Larsson, 1978: 153 [48] [unavailable name, incorrect subsequent spelling not in prevailing usage; [129], Art. 33.3].

Type material. Holotype, sex unknown, exoskeleton, amber inclusion, No. 364/460 (PIN).

Fossil deposit/age. Baltic amber; 38.0–33.9 Ma (Eocene).

Literature. Iablokoff-Khnzorian (1961: 94): original description [47]; Larsson (1978: 153): catalogue [48]; Spahr (1981: 46): catalogue [49]; Keilbach (1982: 246): catalogue [133]; Carpenter (1992: 304): generic catalogue [68]; Alekseev (2013: 7): checklist [92]; Kundrata et al. (2020: 9): generic catalogue [12].

### 3.5. Subfamily Lissominae Laporte, 1835 *

Lissomidae Laporte, 1835: 178 [197]. Type genus: *Lissomus* Dalman, 1824: 13 [198]. For more information, including synonyms, see Kundrata et al. [12].

#### 3.5.1. Tribe Lissomini Laporte, 1835 *

Lissomidae Laporte, 1835: 178 [197]. Type genus: *Lissomus* Dalman, 1824: 13 [198]. For more information, including synonyms, see Kundrata et al. [12].

 

- Genus *Lissomus* Dalman, 1824 *

*Lissomus* Dalman, 1824: 13 [198]. Type species: *Lissomus punctulatus* Dalman, 1824: 14 [198]. For more information, see Cate [107].

 

***Lissomus taxodii* (Heer, 1870)**

*Curculionites taxodii* Heer, 1870: 76 [18].

*Lissomus taxodii*: Birket-Smith, 1977: 21 [154].

Type material. Holotype, sex unknown, elytron, compression fossil, No. 40 (SMNH).

Fossil deposit/age. Norway: Svalbard and Jan Mayen, Firkanten Formation, Cap Staratschin; 66.0–59.2 Ma (Paleocene).

Literature. Heer (1870: 76): original description [18]; Birket-Smith (1977: 21): revision, redescription [154]; Legalov (2015: 1497): catalogue (as *Curculionites* Heer, 1847) [199]; Legalov (2020: 23): catalogue (as *Curculionites*) [200].

Remark. The description of this species is based on a part of isolated elytron and, therefore, its generic attribution is rather problematic. It was originally described in *Curculionites* Heer, 1847 (Curculionoidea) and only later transferred to *Lissomus* in Elateridae [154]. Based on the reconstruction by Birket-Smith [154], it seems that this species belongs rather to Dendrometrinae than Lissominae. Legalov [199,200] kept this species in original genus in Curculionoidea in the catalogues of fossil curculionoids. The identity of this species should be confirmed by study of the type material.

#### 3.5.2. Tribe Protelaterini Schwarz, 1902 *

Protelateridae Schwarz, 1902: 365 [201]. Type genus: *Protelater* Sharp, 1877: 482 [202]. For more information, including synonyms, see Kundrata et al. [94,109].

 

- **Genus *Baltelater* Kundrata, Bukejs, Prosvirov and Hoffmannova, 2020**

*Baltelater* Kundrata, Bukejs, Prosvirov and Hoffmannova, 2020: 2 [94]. Type species: *Baltelater bipectinatus* Kundrata, Bukejs, Prosvirov and Hoffmannova, 2020: 3 [94]. For more information, see Kundrata et al. [94].

 

***Baltelater bipectinatus* Kundrata, Bukejs, Prosvirov and Hoffmannova, 2020**

*Baltelater bipectinatus* Kundrata, Bukejs, Prosvirov and Hoffmannova, 2020: 3 [94].

Type material. Holotype, male, exoskeleton, amber inclusion, No. 6685 (JDC 8374) (MAIG, ex coll. Jonas Damzen).

Fossil deposit/age. Baltic Amber; 38.0–33.9 Ma (Eocene).

Literature. Kundrata et al. (2020): original description [94].

### 3.6. Subfamily Negastriinae Nakane and Kishii, 1956 *

Negastriinae Nakane and Kishii, 1956: 203 [203]. Type genus: *Negastrius* Thomson, 1859: 106 [204]. For more information, see Bouchard et al. [110].

 

- **Genus *Ganestrius* Dolin, 1976**

*Ganestrius* Dolin, 1976: 69 [52]. Type species: *Ganestrius stibicki* Dolin, 1976: 71 [52]. For more information, see Kundrata et al. [12].

 

***Ganestrius elongatus* Dolin, 1976**

*Ganestrius elongatus* Dolin, 1976: 71 [52].

Type material. Holotype, sex unknown, exoskeleton, compression fossil, No. 2066/2528 (part + counterpart) (PIN).

Fossil deposit/age. Kazakhstan: Karabastau Formation, Karatau, Mikhailovka; 166.1–157.3 Ma (Jurassic).

Literature. Dolin (1976: 71): original description [52]; Dolin (1980: 77): revision, catalogue [53]; Korneev and Cate (2005: 15): checklist [120].

 

***Ganestrius stibicki* Dolin, 1976**

*Ganestrius stibicki* Dolin, 1976: 71 [52].

Type material. Holotype, sex unknown, exoskeleton, compression fossil, No. 2066/2823 (PIN).

Fossil deposit/age. Kazakhstan: Karabastau Formation, Karatau, Mikhailovka; 166.1–157.3 Ma (Jurassic).

Literature. Dolin (1976: 71): original description [52]; Dolin (1980: 77): revision, catalogue [53]; Carpenter (1992: 304): generic catalogue [68]; Korneev and Cate (2005: 10): checklist [120]; Kundrata et al. (2020: 9): generic catalogue [12].

 

- Genus *Paradonus* Stibick, 1971 *

*Paradonus* Stibick, 1971: 386 [205]. Type species: *Elater pectoralis* Say, 1839: 173 [206]. For more information, see Stibick [205].

 

***Paradonus exterminatus* (Wickham, 1916), comb. nov.**

*Cryptohypnus exterminatus* Wickham, 1916: 506 [28].

Type material. Holotype, sex unknown, exoskeleton, compression fossil, MCZ 2762 (= 11,280 in Scudder coll.) (MCZ).

Fossil deposit/age. USA: Colorado, Florissant Formation, Florissant; 37.2–33.9 Ma (Eocene).

Literature. Wickham (1916: 506): original description [28]; Wickham (1920: 354): catalogue [29]; Stibick (1981: 246): systematic remark [164].

Remark. In his revision of Hypnoidinae, Stibick [164] examined *Cryptohypnus exterminatus* and suggested that it does not belong to Hypnoidinae. Instead, he suggested its placement in Negastriinae, close to *Negastrius pectoralis* group (=genus *Paradonus sensu* Stibick [205]), based on the “overall sculpture” and non-striate elytra. This relationship was already mentioned by Wickham [28] in the original description of *C. exterminatus*. Here, we formally place this species in *Paradonus*.

 

***Paradonus hesperus* (Wickham, 1916)**

*Cryptohypnus hesperus* Wickham, 1916: 506 [28].

*Paradonus hesperus*: Stibick, 1971: 386 [205].

Type material. Holotype, sex unknown, exoskeleton, compression fossil, MCZ 2761 (=5294 in Scudder coll.) (MCZ).

Fossil deposit/age. USA: Colorado, Florissant Formation, Florissant; 37.2–33.9 Ma (Eocene).

Literature. Wickham (1916: 506): original description [28]; Wickham (1920: 354): catalogue [29]; Stibick (1971: 386): systematic remark [205]; Stibick (1981: 246): systematic remark [164].

 

- **Genus *Protoquasimus* Dolin, 1976**

*Protoquasimus* Dolin, 1976: 69 [52]. Type species: *Protoquasimus brevicollis* Dolin, 1976: 69 [52]. For more information, see Kundrata et al. [12].

 

***Protoquasimus brevicollis* Dolin, 1976**

*Protoquasimus brevicollis* Dolin, 1976: 69 [52].

Type material. Holotype, sex unknown, exoskeleton, compression fossil, No. 2066/2993 (part + counterpart) (PIN).

Fossil deposit/age. Kazakhstan: Karabastau Formation, Karatau, Mikhailovka; 166.1–157.3 Ma (Jurassic).

Literature. Dolin (1976: 69): original description [52]; Dolin (1980: 76): revision, catalogue [53]; Carpenter (1992: 305): generic catalogue [68]; Korneev and Cate (2005: 10): checklist [120]; Dong and Huang (2011: 1227): checklist [81]; Kundrata et al. (2020: 9): generic catalogue [12].

 

### 3.7. Subfamily Omalisinae Lacordaire, 1857 *

Homalisides Lacordaire, 1857: 303 [207]. Type genus: *Omalisus* Geoffroy, 1762: 179 [208]. For more information, see Bouchard et al. [110].

 

- **Genus *Jantarokrama* Kirejtshuk and Kovalev, 2015**

*Jantarokrama* Kirejtshuk and Kovalev, 2015: 1413 [93]. Type species: *Jantarokrama utilis* Kirejtshuk and Kovalev, 2015: 1414 [93]. For more information, see Kundrata et al. [12].

 

***Jantarokrama utilis* Kirejtshuk and Kovalev, 2015**

*Jantarokrama utilis* Kirejtshuk and Kovalev, 2015: 1414 [93].

Type material. Holotype, male, amber inclusion, A52062 (283) (MNHN).

Fossil deposit/age. Baltic Amber; 38.0–33.9 Ma (Eocene).

Literature. Kirejtshuk and Kovalev (2015: 1414): original description [93]; Kundrata et al. (2020: 9): generic catalogue [12]; Kundrata et al. (2020: 8): remark [94].

### 3.8. Subfamily Pityobiinae Hyslop, 1917 *

Pityobini Hyslop, 1917: 249 [209]. Type genus: *Pityobius* LeConte, 1853: 428 [144]. For more information, see Bouchard et al. [110].

 

- **Genus *Cretopityobius* Otto, 2019**

*Cretopityobius* Otto, 2019: 4 [96]. Type species: *Cretopityobius pankowskiorum* Otto, 2019: 4 [96]. For more information, see Otto [96] and Kundrata et al. [12]. The assignment of this genus to Pityobiinae needs further investigation.

 

***Cretopityobius pankowskiorum* Otto, 2019**

*Cretopityobius pankowskiorum* Otto, 2019: 4 [96].

Type material. Holotype, sex unknown, amber inclusion (USNM). One paratype, sex unknown, amber inclusion (WIRC) (Figure 1C).

Fossil deposit/age. Myanmar: Burmese amber; 99.6–93.5 Ma (Cretaceous).

Literature. Otto (2019: 4): original description [96]; Kundrata et al. (2020: 9): generic catalogue [12]; Kundrata et al. (2020: 8): remark [94].

### 3.9. Subfamily Protagrypninae Dolin, 1973

Protagrypnini Dolin, 1973: 74 [50]. Type genus: *Protagrypnus* Dolin, 1973: 75 [50]. For more information, see Bouchard et al. [110] and Kundrata et al. [12].

Remark. This group was originally classified in Agrypninae [50,210]. Protagrypninae are mainly defined by the conspicuous structures on the prosternum and mesoventrite [50,53,75,76]. The validity of the first character was recently discussed and questioned by Muona et al. [99]. Protagrypninae are in urgent need of revision. Their monophyly, limits and systematic placement are unclear and it is possible that many lineages currently classified in this subfamily belong to another Elateridae group or even to other elateroid families, mainly Eucnemidae (see discussion in Muona et al. [99] and herein).

#### 3.9.1. Tribe Desmatini Dolin, 1975

Desmatini Dolin, 1975: 60 [51]. Type genus: *Desmatus* Dolin, 1975: 60 [51]. For more information, see Bouchard et al. [110] and Kundrata et al. [12].

Remark. Representatives of this tribe differ from related groups mainly in the strongly developed metacoxal plates. This characters is typical for Elaterinae: Physorhinini but can be found in some other Elateridae and also in some other clicking elateroids such as Eucnemidae. Many species of this tribe indeed resemble Physorhinini in external characters, but not in all cases, so the composition and systematic position of Desmatini require further investigation (see discussion in Muona et al. [99]). Regarding genera formerly placed in Desmatini, Muona et al. [99] transferred *Anoixis* Chang, Kirejtshuk and Ren, 2010 (monotypic) to Eucnemidae: Palaeoxeninae, and *Apoclion* Chang, Kirejtshuk and Ren, 2010 (all 3 spp.) and *Paradesmatus* Chang, Kirejtshuk and Ren, 2009 (2 of 3 spp., including the type species) to Eucnemidae: Schizophilinae. One species of *Paradesmatus* was transferred to *Desmatus* Dolin, 1975.

 

- **Genus *Desmatinus* Chang, Kirejtshuk and Ren, 2010**

*Desmatinus* Chang, Kirejtshuk and Ren, 2010: 868 [86]. Type species: *Desmatinus cognatus* Chang, Kirejtshuk and Ren, 2010: 869 [86]. For more information, see Kundrata et al. [12].

 

***Desmatinus cognatus* Chang, Kirejtshuk et Ren, 2010**

*Desmatinus cognatus* Chang, Kirejtshuk and Ren, 2010: 869 [86].

Type material. Holotype, sex unknown, exoskeleton, compression fossil, CNU-COL-LB2008836 (CNU).

Fossil deposit. China: Liaoning Province, Shangyuan County, Beipiao City, Yixian Formation, Huangbanjigou, near Chaomidian Village; 125.45–122.46 Ma (Cretaceous).

Literature. Chang et al. (2010: 869): original description [86]; Dong and Huang (2011: 1225): checklist [81]; Yu et al. (2019: 383): remark [89]; Kundrata et al. (2020: 10): generic catalogue [12]; Muona et al. (2020: 9): revision [99].

 

- **Genus *Desmatus* Dolin, 1975**

*Desmatus* Dolin, 1975: 60 [51]. Type species: *Desmatus lapidarius* Dolin, 1975: 61 [51]. For more information, see Kundrata et al. [12].

Remark. This genus needs a revision since some species differ from the type species (and also from each other) in the body proportions, the shape of antenna, thorax, etc. The systematic placement of all species should be re-evaluated since they might represent Eucnemidae, based mainly on the presence of short and broad pronotum and enlarged metacoxal plates.

 

***Desmatus affinis* Dolin, 1975**

*Desmatus affinis* Dolin, 1975: 62 [51].

Type material. Holotype, sex unknown, exoskeleton, compression fossil, No. 2554/672 (PIN).

Fossil deposit/age. Kazakhstan: Karabastau Formation, Karatau, Mikhailovka; 166.1–157.3 Ma (Jurassic).

Literature. Dolin (1975: 62): original description [51]; Dolin (1980: 65): key, catalogue [53]; Korneev and Cate (2005: 11): checklist [120].

 

***Desmatus beckeri* Dolin, 1975**

*Desmatus beckeri* Dolin, 1975: 62 [51].

Type material. Holotype, sex unknown, exoskeleton, compression fossil, No. 2066/2364 (part + counterpart) (PIN). Two paratypes, sex unknown, exoskeletons, compression fossils, Nos. 2554/689, 2384/489 (PIN).

Fossil deposit/age. Kazakhstan: Karabastau Formation, Karatau, Mikhailovka; 166.1–157.3 Ma (Jurassic).

Literature. Dolin (1975: 62): original description [51]; Dolin (1980: 65): key, catalogue [53]; Korneev and Cate (2005: 12): checklist [120].

 

***Desmatus lapidarius* Dolin, 1975**

*Desmatus lapidarius* Dolin, 1975: 61 [51].

Type material. Holotype, sex unknown, exoskeleton, compression fossil, No. 2066/3274 (PIN). Paratype, sex unknown, exoskeleton, compression fossil, No. 2784/1377 (PIN).

Fossil deposit/age. Kazakhstan: Karabastau Formation, Karatau, Mikhailovka; 166.1–157.3 Ma (Jurassic).

Literature. Dolin (1975: 61): original description [51]; Dolin (1980: 64, 65): key, catalogue [53]; Carpenter (1992: 304): generic catalogue [68]; Korneev and Cate (2005: 10): checklist [120]; Kundrata et al. (2020: 10): generic catalogue [12].

 

***Desmatus ponomarenkoi* (Chang, Kirejtshuk and Ren, 2009)**

*Paradesmatus ponomarenkoi* Chang, Kirejtshuk and Ren, 2009: 10 [78].

*Desmatus ponomarenkoi*: Muona et al., 2020: 10 [99].

Type material. Holotype, female, exoskeleton, impression, CNU-COL-NN2006876PC (CNU). Paratype, sex unknown, exoskeleton, impression, CNU-C-NN2007870 (CNU).

Fossil deposit/age. China: Inner Mongolia, Ningcheng County, Jiulongshan Formation, Daohugou; 166.1–157.3 Ma (Jurassic).

Literature. Chang et al. (2009: 10): original description [78]; Chang et al. (2010: 867): remark [86]; Kirejtshuk et al. (2010: 791): checklist [87]; Dong and Huang (2011: 1225): checklist [81]; Schimmel and Tarnawski (2012: 265): remark [132]; Muona et al. (2020: 10): revision, nomenclatural change [99].

 

***Desmatus protensus* Dolin, 1980**

*Desmatus protensus* Dolin, 1980: 65 [53].

Type material. Holotype, sex unknown, exoskeleton, compression fossil, No. 2997/2000 (PIN).

Fossil deposit/age. Kazakhstan: Karabastau Formation, Karatau, Mikhailovka; 166.1–157.3 Ma (Jurassic).

Literature. Dolin (1980: 65): original description [53]; Korneev and Cate (2005: 22): checklist [120].

 

- **Genus *Plesiorhaphes* Dolin, 1980**

*Plesiorhaphes* Dolin, 1980: 65 [53]. Type species: *Plesiorhaphes scaber* Dolin, 1980: 66 [53]. For more information, see Kundrata et al. [12].

 

***Plesiorhaphes scaber* Dolin, 1980**

*Plesiorhaphes scaber* Dolin, 1980: 66 [53].

*Plesiorhaphes scabei*: Carpenter, 1992: 305 [68].

Type material. Holotype, sex unknown, exoskeleton, compression fossil, No. 2784/1383 (PIN).

Fossil deposit/age. Kazakhstan: Karabastau Formation, Karatau, Mikhailovka; 166.1–157.3 Ma (Jurassic).

Literature. Dolin (1980: 66): original description [53]; Carpenter (1992: 305): generic catalogue [68]; Korneev and Cate (2005: 10): checklist [120]; Kundrata et al. (2020: 10): generic catalogue [12].

#### 3.9.2. Tribe Hypnomorphini Dolin, 1975

Hypnomorphini Dolin, 1975: 54 [51]. Type genus: *Hypnomorphus* Dolin, 1975: 54 [51]. For more information, see Bouchard et al. [110] and Kundrata et al. [12].

Remark. This tribe needs a revision as it most probably includes various unrelated groups.

 

- **Genus *Abrotus* Dolin, 1980**

*Abrotus* Dolin, 1980: 62 [53]. Type species: *Abrotus sepultus* Dolin, 1980: 63 [53]. The systematic placement of this genus is uncertain and should be re-examined. For more information, see Chang et al. [77] and Kundrata et al. [12].

 

***Abrotus reconditus* Dolin, 1980**

*Abrotus reconditus* Dolin, 1980: 63 [53].

Type material. Holotype, sex unknown, exoskeleton, compression fossil, No. 2239/1451 (PIN). Paratype, sex unknown, exoskeleton, compression fossil, No. 2384/482 (PIN).

Fossil deposit/age. Kazakhstan: Karabastau Formation, Karatau, Mikhailovka; 166.1–157.3 Ma (Jurassic).

Literature. Dolin (1980: 63): original description [53]; Korneev and Cate (2005: 22): checklist [120].

 

***Abrotus sepultus* Dolin, 1980**

*Abrotus sepultus* Dolin, 1980: 63 [53].

Type material. Holotype, sex unknown, exoskeleton, compression fossil, No. 2066/3291 (PIN). Paratype, No. 2239/1418.

Fossil deposit/age. Kazakhstan: Karabastau Formation, Karatau, Mikhailovka; 166.1–157.3 Ma (Jurassic).

Literature. Dolin (1980: 63): original description [53]; Carpenter (1992: 304): generic catalogue [68]; Korneev and Cate (2005: 9): checklist [120]; Chang et al. (2011: 36): remark [77]; Kundrata et al. (2020: 11): generic catalogue [12].

 

- **Genus *Adiagnostus* Dolin, 1980**

*Adiagnostus* Dolin, 1980: 44 [53]. Type species: *Adiagnostus cardiophorinus* Dolin, 1980: 45 [53]. For more information, see Kundrata et al. [12].

 

***Adiagnostus ambiguus* Dolin, 1980**

*Adiagnostus ambiguus* Dolin, 1980: 45 [53].

Type material. Holotype, sex unknown, exoskeleton, compression fossil, No. 2239/1423 (PIN).

Fossil deposit/age. Kazakhstan: Karabastau Formation, Karatau, Mikhailovka; 166.1–157.3 Ma (Jurassic).

Literature. Dolin (1980: 45): original description [53]; Korneev and Cate (2005: 11): checklist [120].

 

***Adiagnostus cardiophorinus* Dolin, 1980**

*Adiagnostus cardiophorinus* Dolin, 1980: 45 [53].

Type material. Holotype, sex unknown, exoskeleton, compression fossil, No. 2066/3231 (part) + 2066/3164 (counterpart) (PIN).

Fossil deposit/age. Kazakhstan: Karabastau Formation, Karatau, Mikhailovka; 166.1–157.3 Ma (Jurassic).

Literature. Dolin (1980: 45): original description [53]; Carpenter (1992: 304): generic catalogue [68]; Korneev and Cate (2005: 9): checklist [120]; Kundrata et al. (2020: 11): generic catalogue [12].

 

***Adiagnostus minutulus* Dolin, 1980**

*Adiagnostus minutulus* Dolin, 1980: 46 [53].

Type material. Holotype, sex unknown, exoskeleton, compression fossil, No. 2384/455 (PIN).

Fossil deposit/age. Kazakhstan: Karabastau Formation, Karatau, Mikhailovka; 166.1–157.3 Ma (Jurassic).

Literature. Dolin (1980: 46): original description [53]; Korneev and Cate (2005: 20): checklist [120].

 

- **Genus *Codemus* Dolin, 1980**

*Codemus* Dolin, 1980: 35 [53]. Type species: *Codemus synaptoides* Dolin, 1980: 36 [53]. For more information, see Kundrata et al. [12].

 

***Codemus alatus* Dolin, 1980**

*Codemus alatus* Dolin, 1980: 39 [53].

Type material. Holotype, sex unknown, exoskeleton, compression fossil, No. 2066/2734 (part) + 2066/2725 (counterpart) (PIN). Six paratypes, sex unknown, exoskeletons, compression fossils, Nos. 2554/654 (part + counterpart), 2784/1368, 2784/1391, 2904/901 (part + counterpart), 2904/926, 2997/4462 (PIN).

Fossil deposit/age. Kazakhstan: Karabastau Formation, Karatau, Mikhailovka; 166.1–157.3 Ma (Jurassic).

Literature. Dolin (1980: 39): original description [53]; Korneev and Cate (2005: 11): checklist [120].

 

***Codemus carinatus* Dolin, 1980**

*Codemus carinatus* Dolin, 1980: 38 [53].

Type material. Holotype, sex unknown, exoskeleton, compression fossil, No. 2384/464 (PIN). Four paratypes, sex unknown, exoskeletons, compression fossils, Nos. 2384/483, 2784/1405, 2784/1372, 2384/1399 (PIN).

Fossil deposit/age. Kazakhstan: Karabastau Formation, Karatau, Mikhailovka; 166.1–157.3 Ma (Jurassic).

Literature. Dolin (1980: 38): original description [53]; Korneev and Cate (2005: 13): checklist [120].

Remark. This species strongly differs from its congeners in the presence of long sublateral carinae on pronotum, the short incision of posterior edge of pronotum, and less elongated elytra. It is probable that *C. carinatus* belongs to another genus.

 

***Codemus jejunus* Dolin, 1980**

*Codemus jejunus* Dolin, 1980: 39 [53].

Type material. Holotype, sex unknown, exoskeleton, compression fossil, No. 2066/3261 (PIN). Two paratypes, sex unknown, exoskeletons, compression fossils, Nos. 2239/1434, 2997/1960 (part) + 2997/1965 (counterpart) (PIN).

Fossil deposit/age. Kazakhstan: Karabastau Formation, Karatau, Mikhailovka; 166.1–157.3 Ma (Jurassic).

Literature. Dolin (1980: 39): original description [53]; Korneev and Cate (2005: 17): checklist [120].

 

***Codemus martynovi* Dolin, 1980**

*Codemus martynovi* Dolin, 1980: 37 [53].

Type material. Holotype, sex unknown, exoskeleton, compression fossil, No. 1784/37 (PIN).

Fossil deposit/age. Kazakhstan: Karabastau Formation, Karatau, Galkino; 166.1–157.3 Ma (Jurassic).

Literature. Dolin (1980: 37): original description [53]; Korneev and Cate (2005: 19): checklist [120].

 

***Codemus micros* Dolin, 1980**

*Codemus micros* Dolin, 1980: 40 [53].

Type material. Holotype, sex unknown, exoskeleton, compression fossil, No. 2239/1441 (PIN). Six paratypes, sex unknown, exoskeletons, compression fossils, No. 2784/1384, 2904/917, 2904/924, 2904/925, 2997/4459, 2997/4460 (PIN).

Fossil deposit/age. Kazakhstan: Karabastau Formation, Karatau, Mikhailovka; 166.1–157.3 Ma (Jurassic).

Literature. Dolin (1980: 40): original description [53]; Korneev and Cate (2005: 20): checklist [120].

 

***Codemus quadricolis* Dolin, 1980**

*Codemus quadricolis* Dolin, 1980: 38 [53].

*Codemus quadricollis* Dolin, 1980: 35 [53] [also in the legend to Figure 28] [unavailable name, incorrect original spelling ([129], Art. 19.3); First Revisers ([129], Art. 24.2): Korneev and Cate (2005: 22) [120]].

Type material. Holotype, sex unknown, exoskeleton, compression fossil, No. 2066/2947 (part + counterpart) (PIN). Seven paratypes, sex unknown, exoskeletons, compression fossils, Nos. 2066/2628, 2066/2658, 2239/1437, 2384/500, 2384/502, 2554/687, 2554/704 (PIN).

Fossil deposit/age. Kazakhstan: Karabastau Formation, Karatau, Mikhailovka; 166.1–157.3 Ma (Jurassic).

Literature. Dolin (1980: 38): original description [53]; Korneev and Cate (2005: 22): checklist [120].

 

***Codemus sharovi* Dolin, 1980**

*Codemus sharovi* Dolin, 1980: 36 [53].

Type material. Holotype, sex unknown, exoskeleton, compression fossil, No. 2384/474 (PIN). Five paratypes, sex unknown, exoskeletons, compression fossils, Nos. 2384/465, 2066/2722 (part + counterpart), 2784/1378, 2997/4471, 2997/417 (part + counterpart) (PIN).

Fossil deposit/age. Kazakhstan: Karabastau Formation, Karatau, Mikhailovka; 166.1–157.3 Ma (Jurassic).

Literature. Dolin (1980: 36): original description [53]; Korneev and Cate (2005: 23): checklist [120].

 

***Codemus synaptoides* Dolin, 1980**

*Codemus synaptoides* Dolin, 1980: 36 [53].

Type material. Holotype, sex unknown, exoskeleton, compression fossil, Nos. 2239/1411 (part) + 2239/1443 (counterpart) (PIN). 11 paratypes, sex unknown, exoskeletons, compression fossils, Nos. 2239/1433 (part + counterpart), 2066/2418, 2066/2696, 2066/3132, 2384/481 (part) + 2384/483 (counterpart), 2554/680, 2554/682, 2904/899 (part + counterpart), 2997/1994, 2997/2012, 2997/4473 (PIN).

Fossil deposit/age. Kazakhstan: Karabastau Formation, Karatau, Mikhailovka; 166.1–157.3 Ma (Jurassic).

Literature. Dolin (1980: 36): original description [53]; Carpenter (1992: 304): generic catalogue [68]; Korneev and Cate (2005: 10): checklist [120]; Kundrata et al. (2020: 12): generic catalogue [12].

 

***Codemus teres* Dolin, 1980**

*Codemus teres* Dolin, 1980: 38 [53].

Type material. Holotype, sex unknown, exoskeleton, compression fossil, No. 2904/905 (part + counterpart) (PIN). Five paratypes, sex unknown, exoskeletons, compression fossils, Nos. 2997/1996, 2997/2016, 2066/2703, 2066/2696, 2554/695 (PIN).

Fossil deposit/age. Kazakhstan: Karabastau Formation, Karatau, Mikhailovka; 166.1–157.3 Ma (Jurassic).

Literature. Dolin (1980: 38): original description [53]; Korneev and Cate (2005: 24): checklist [120].

 

***Codemus zherichini* Dolin, 1980**

*Codemus zherichini* Dolin, 1980: 37 [53].

Type material. Holotype, sex unknown, exoskeleton, compression fossil, No. 2997/1999 (PIN). Three paratypes, sex unknown, exoskeletons, compression fossils, Nos. 2997/4464, 2784/1367 (part + counterpart), 2784/1365 (part + counterpart) (PIN).

Fossil deposit/age. Kazakhstan: Karabastau Formation, Karatau, Mikhailovka; 166.1–157.3 Ma (Jurassic).

Literature. Dolin (1980: 37): original description [53]; Korneev and Cate (2005: 25): checklist [120].

 

- **Genus *Dolinelater* Huber, Marggi and Menkveld-Gfeller, 2017**

*Idiomorphus* Dolin, 1980: 60 [53]. Type species: *Idiomorphus singularis* Dolin, 1980: 60 [53]. For more information, see Kundrata et al. [12]. Preoccupied by *Idiomorphus* Chaudoir, 1846 (Coleoptera: Carabidae) [211].

*Dolinelater* Huber, Marggi and Menkveld-Gfeller, 2017: 2 [211]. Replacement name for *Idiomorphus* Dolin, 1980. Erroneously omitted in the generic catalogue by Kundrata et al. [12].

 

***Dolinelater asperatus* (Dolin, 1980)**

*Idiomorphus asperatus* Dolin, 1980: 61 [53].

*Dolinelater asperatus*: Huber et al. 2017: 2 [211].

Type material. Holotype, sex unknown, exoskeleton, compression fossil, No. 2066/3300 (PIN). Two paratypes, sex unknown, exoskeletons, compression fossils, Nos. 2997/2021, 2997/1967 (PIN).

Fossil deposit/age. Kazakhstan: Karabastau Formation, Karatau, Mikhailovka; 166.1–157.3 Ma (Jurassic).

Literature. Dolin (1980: 61): original description [53]; Korneev and Cate (2005: 12): checklist [120]; Huber et al. (2017: 2): nomenclatural remark [211].

 

***Dolinelater singularis* (Dolin, 1980)**

*Idiomorphus singularis* Dolin, 1980: 60 [53].

*Dolinelater singularis*: Huber et al., 2017: 2 [211].

Type material. Holotype, sex unknown, exoskeleton, compression fossil, No. 2239/1438 (PIN).

Fossil deposit/age. Kazakhstan: Karabastau Formation, Karatau, Mikhailovka; 166.1–157.3 Ma (Jurassic).

Literature. Dolin (1980: 60): original description [53]; Carpenter (1992: 304): generic catalogue [68]; Korneev and Cate (2005: 10): checklist [120]; Huber et al. (2017: 2): nomenclatural remark [211]; Kundrata et al. (2020: 12): generic catalogue [12].

 

- **Genus** ***Elaterophanes* Handlirsch, 1906**

*Elaterophanes* Handlirsch, 1906: 436 [26]. Type species: *Elater socius* Giebel, 1856: 91 [16] (=*Elater vetustus* Brodie, 1845: 101 [13]). For more information, see Kundrata et al. [12].

Remark. The diagnosis, limits and systematic placement of this genus are unclear.

 

***Elaterophanes acutus* Cockerell, 1916**

*Elaterophanes acutus* Cockerell, 1916: 478 [31].

Type material. Holotype, sex unknown, elytron, impression, No. 61,401 (USNM).

Fossil deposit/age. United Kingdom: Gloucestershire, Wainlode Cliff; 208.5–201.3 Ma (Triassic).

Literature. Cockerell (1916: 478): original description [31]; Handlirsch (1938: 65): catalogue [212].

Remark. The description of this species is based on an isolated elytron and, therefore, its generic attribution is rather problematic [212].

 

***Elaterophanes regius* Whalley, 1985**

*Elaterophanes regius* Whalley, 1985: 165 [60].

Type material. Holotype, sex unknown, exoskeleton, compression fossil, No. 59,385 (BMNH). One paratype, No. 53,952 (BMNH).

Fossil deposit/age. United Kingdom: Charmouth Mudstone Formation, Dorset, Flatstones, Black Ven, Charmouth; 196.5–189.6 Ma (Jurassic).

Literature. Whalley (1985: 165): original description [60]; Martin (2010: 934): remark [73].

 

***Elaterophanes vetustus* (Brodie, 1845)**

*Elater vetustus* Brodie, 1845: 101 [13].

*Elater socius* Giebel, 1856: 91 [16]. Synonymized with *E. vetustus* by Whalley (1985: 165) [60].

*Elaterophanes socius*: Handlirsch, 1906: 436 [26].

*Elaterophanes vetustus*: Handlirsch, 1906: 437 [26].

Type material. *Elater vetustus*. Holotype, sex unknown, compression fossil, No. NHM I.3576 (BMNH). *Elater socius*. Holotype, sex unknown, compression fossil, No. NHM I.3563 (BMNH).

Fossil deposit/age. United Kingdom: Apperley, Lilstock Formation; 208.5–201.3 Ma.

Literature. Brodie (1845: 101): original description of E. vetustus [13]; Giebel (1856: 91): original description of E. vetustus, revision [16]; Phillips (1871: 123): checklist [213]; Heer (1883: 98): remark [137]; Scudder (1891: 204): catalogue [24]; Handlirsch (1906: 436): redescription of E. socius and E. vetustus [26]; Cockerell (1916: 478): catalogue [31]; Handlirsch (1938: 65, 69): checklist [212]; Haupt (1950: 102): remark [44]; Dolin (1973: 73): remark [50]; Whalley (1985: 165): taxonomy, remark [60]; Carpenter (1992: 304): generic catalogue [68]; Kundrata et al. (2020: 11): generic catalogue [12].

Remark. Brodie [13] described *Elater vetustus* based on a compression fossil from Lilstock Formation, Apperley (Triassic) in the United Kingdom. Heer [17] described *Elaterites vetustus* based on a single elytron from Schambelen, Aargau (Jurassic) in Switzerland, but he attributed the species name to Brodie. It is not clear whether Heer [17] thought that the fossil from Switzerland was conspecific with the Brodie’s *Elater vetustus* from Apperley beds of the United Kingdom, and if/why he transferred that species from *Elater* to *Elaterites*. Later, Handlirsch [26] transferred *Elater vetustus* Brodie, 1845 to *Elaterophanes* (as he did with *E. socius* Giebel, 1856), and he erected a new genus, *Dysarestus* Handlirsch, 1906, for *Elaterites vetustus* Heer, 1865. He suggested that it might belong to *Elaterophanes*. Here, we follow Carpenter [68], who classified *Dysarestus* in Coleoptera *incertae sedis*.

 

- **Genus** ***Graciolacon* Dolin, 1980**

*Graciolacon* Dolin, 1980: 61 [53]. Type species: *Graciolacon aeternus* Dolin, 1980: 62 [53]. For more information, see Kundrata et al. [12].

*Graciolacou*: Dolin, 1980: legend of Figure 67 [53] [unavailable name, incorrect original spelling ([129], Art. 19.3); First Reviser ([129], Art. 24.2): Carpenter (1992: 304) [68]].

 

***Graciolacon aeternus* Dolin, 1980**

*Graciolacon aeternus* Dolin, 1980: 62 [53].

*Graciolacou* [sic!] *aethernus*: Dolin, 1980: legend of drawing 67 [53].

Type material. Holotype, sex unknown, exoskeleton, compression fossil, No. 965/39 (PIN).

Fossil deposit/age. Kazakhstan: Karabastau Formation, Galkino, East Karatau; 166.1–157.3 Ma (Jurassic).

Literature. Dolin (1980: 62): original description [53]; Carpenter (1992: 304): generic catalogue [68]; Korneev and Cate (2005: 11): checklist [120]; Kundrata et al. (2020: 11): generic catalogue [12].

 

- **Genus** ***Hypnomorphoides* Dolin, 1980**

*Hypnomorphoides* Dolin, 1980: 54 [53]. Type species: *Hypnomorphoides catachtonius* Dolin, 1980: 55 [53]. For more information, see Kundrata et al. [12].

Remark. This genus might belong to Eucnemidae as all its species have a compact body, with a short and broad thorax, and short elytra. Unfortunately, the main diagnostic characters [99] are either absent or not well visible on the original drawings or photographs [53].

 

***Hypnomorphoides angularis* Dolin, 1980**

*Hypnomorphoides angularis* Dolin, 1980: 55 [53].

Type material. Holotype, sex unknown, exoskeleton, compression fossil, No. 2997/4461 (part + counterpart) (PIN).

Fossil deposit/age. Kazakhstan: Karabastau Formation, Karatau, Mikhailovka; 166.1–157.3 Ma (Jurassic).

Literature. Dolin (1980: 55): original description [53].

 

***Hypnomorphoides catachtonius* Dolin, 1980**

*Hypnomorphoides catachtonius* Dolin, 1980: 55 [53].

Type material. Holotype, sex unknown, exoskeleton, compression fossil, No. 2066/3062 (PIN). Three paratypes, sex unknown, exoskeletons, compression fossils, Nos. 2904/916, 2997/2014, 2997/2028 (PIN).

Fossil deposit/age. Kazakhstan: Karabastau Formation, Karatau, Mikhailovka; 166.1–157.3 Ma (Jurassic).

Literature. Dolin (1980: 55): original description [53]; Carpenter (1992: 304): generic catalogue [68]; Korneev and Cate (2005: 10): checklist [120]; Kundrata et al. (2020: 11): generic catalogue [12].

 

***Hypnomorphoides latus* Dolin, 1980**

*Hypnomorphoides latus* Dolin, 1980: 56 [53].

Type material. Holotype, sex unknown, exoskeleton, compression fossil, No. 2239/1449 (PIN). Paratype, sex unknown, exoskeleton, compression fossil, No. 2384/449 (PIN).

Fossil deposit/age. Kazakhstan: Karabastau Formation, Karatau, Mikhailovka; 166.1–157.3 Ma (Jurassic).

Literature. Dolin (1980: 56): original description [53]; Korneev and Cate (2005: 19): checklist [120].

 

***Hypnomorphoides procerulus* Dolin, 1980**

*Hypnomorphoides procerulus* Dolin, 1980: 56 [53].

Type material. Holotype, sex unknown, exoskeleton, compression fossil, No. 2904/915 (PIN).

Fossil deposit/age. Kazakhstan: Karabastau Formation, Karatau, Mikhailovka; 166.1–157.3 Ma (Jurassic).

Literature. Dolin (1980: 56): original description [53]; Korneev and Cate (2005: 22): checklist [120].

 

- **Genus** ***Hypnomorphus* Dolin, 1975**

*Hypnomorphus* Dolin, 1975: 54 [51]. Type species: *Hypnomorphus rohdendorfi* Dolin, 1975: 56 [51]. For more information, see Kundrata et al. [12].

Remark. This genus needs a revision since some species differ from the type species (and also from each other) in the body size, proportions, the shape of pronotum, elytra, etc. Most species probably belong to Eucnemidae based on the compact body, with a short and broad thorax, and short elytra. Additionally, *H. rasnitzyni* has antennae with last three antennomeres enlarged, which is a character present in Eucnemidae rather than Elateridae. Unfortunately, the main diagnostic characters [99] are usually absent or not well visible on the original drawings or photographs [51,53].

 

***Hypnomorphus aemulus* Dolin, 1975**

*Hypnomorphus aemulus* Dolin, 1975: 56 [51].

Type material. Holotype, sex unknown, exoskeleton, compression fossil, No. 2554/692 (PIN). Three paratypes, sex unknown, exoskeletons, compression fossils, Nos. 2239/1417, 2239/1454, 2554/690 (PIN).

Fossil deposit/age. Kazakhstan: Karabastau Formation, Karatau, Mikhailovka; 166.1–157.3 Ma (Jurassic).

Literature. Dolin (1975: 56): original description [51]; Dolin (1980: 28, 30): key, additional specimens: Nos. 2784/1385, 2784/1388 (PIN) [53]; Korneev and Cate (2005: 11): checklist [120].

 

***Hypnomorphus angulosus* Dolin, 1980**

*Hypnomorphus angulosus* Dolin, 1980: 29 [53].

Type material. Holotype, sex unknown, exoskeleton, compression fossil, No. 2997/415 (part + counterpart) (PIN).

Fossil deposit/age. Kazakhstan: Karabastau Formation, Karatau, Mikhailovka; 166.1–157.3 Ma (Jurassic).

Literature. Dolin (1980: 29): original description [53]; Korneev and Cate (2005: 11): checklist [120].

 

***Hypnomorphus carpolithus* Dolin, 1975**

*Hypnomorphus carpolithus* Dolin, 1975: 57 [51].

Type material. Holotype, sex unknown, exoskeleton, compression fossil, No. 2066/2606 (PIN).

Fossil deposit/age. Kazakhstan: Karabastau Formation, Karatau, Mikhailovka; 166.1–157.3 Ma (Jurassic).

Literature. Dolin (1975: 57): original description [51]; Dolin (1980: 27,30): key, additional specimens: Nos. 2997/419, 2997/2007, 2997/4457, 2784/1392, 2784/1386 (PIN) [53]; Korneev and Cate (2005: 13): checklist [120].

 

***Hypnomorphus confusus* Dolin, 1975**

*Hypnomorphus confusus* Dolin, 1975: 59 [51].

Type material. Holotype, sex unknown, exoskeleton, compression fossil, No. 1789/213 (PIN).

Fossil deposit/age. Kazakhstan: Karabastau Formation, Karatau, Galkino; 166.1–157.3 Ma (Jurassic).

Literature. Dolin (1975: 59): original description [51]; Dolin (1980: 27): key, additional specimens: Nos. 2784/1382, 2784/1393, 2784/1401, 2904/903, 2904/910, 2997/1393, 2997/2009 (PIN) [53]; Korneev and Cate (2005: 14): checklist [120].

 

***Hypnomorphus curtus* Dolin, 1980**

*Hypnomorphus curtus* Dolin, 1980: 28 [53].

Type material. Holotype, sex unknown, exoskeleton, compression fossil, No. 2066/2806 (PIN). Paratype, sex unknown, exoskeleton, compression fossil, No. 2384/472 (PIN).

Fossil deposit/age. Kazakhstan: Karabastau Formation, Karatau, Mikhailovka; 166.1–157.3 Ma (Jurassic).

Literature. Dolin (1980: 28): original description [53]; Korneev and Cate (2005: 14): checklist [120].

 

***Hypnomorphus distinctus* Dolin, 1975**

*Hypnomorphus distinctus* Dolin, 1975: 56 [51].

Type material. Holotype, sex unknown, exoskeleton, compression fossil, No. 2554/653 (part + counterpart) (PIN). Three paratypes, sex unknown, exoskeletons, compression fossils, Nos. 2554/652, 2239/1469, 2066/2764 (PIN).

Fossil deposit/age. Kazakhstan: Karabastau Formation, Karatau, Mikhailovka; 164.7–155.7 Ma (Jurassic).

Literature. Dolin (1975: 56): original description [51]; Dolin (1980: 27,30): key, additional specimens: Nos. 2904/907 (part + counterpart), 2997/4470, 2904/923 (PIN) [53]; Korneev and Cate (2005: 14): checklist [120].

 

***Hypnomorphus dubius* Dolin, 1975**

*Hypnomorphus dubius* Dolin, 1975: 60 [51].

Type material. Holotype, sex unknown, exoskeleton, compression fossil, No. 2554/651 (PIN).

Fossil deposit/age. Kazakhstan: Karabastau Formation, Karatau, Mikhailovka; 166.1–157.3 Ma (Jurassic).

Literature. Dolin (1975: 60): original description [51]; Dolin (1980: 27,30): key, additional specimens: Nos. 2239/1453, 2784/1403, 2997/428, 2997/1969 (PIN) [53]; Korneev and Cate (2005: 14): checklist [120].

 

***Hypnomorphus gigas* Dolin, 1980**

*Hypnomorphus gigas* Dolin, 1980: 29 [53].

Type material. Holotype, sex unknown, exoskeleton, compression fossil, No. 2997/4472 (PIN).

Fossil deposit/age. Kazakhstan: Karabastau Formation, Karatau, Mikhailovka; 166.1–157.3 Ma (Jurassic).

Literature. Dolin (1980: 29): original description [53]; Korneev and Cate (2005: 15): checklist [120].

 

***Hypnomorphus imperspicuus* Dolin, 1975**

*Hypnomorphus imperspicuus* Dolin, 1975: 59 [51].

Type material. Holotype, sex unknown, exoskeleton, compression fossil, No. 2554/678 (PIN). Two paratypes, sex unknown, exoskeletons, compression fossils, Nos. 2066/3036, 2066/2838 (PIN).

Fossil deposit/age. Kazakhstan: Karabastau Formation, Karatau, Mikhailovka; 166.1–157.3 Ma (Jurassic).

Literature. Dolin (1975: 59): original description [51]; Dolin (1980: 28,30): key, additional specimen: No. 2904/904 (PIN) [53]; Korneev and Cate (2005: 16): checklist [120].

 

***Hypnomorphus induratus* Dolin, 1975**

*Hypnomorphus induratus* Dolin, 1975: 57 [51].

Type material. Holotype, sex unknown, exoskeleton, compression fossil, No. 2239/1427 (part + counterpart) (PIN).

Fossil deposit/age. Kazakhstan: Karabastau Formation, Karatau, Mikhailovka; 166.1–157.3 Ma (Jurassic).

Literature. Dolin (1975: 57): original description [51]; Dolin (1980: 27,30): key, additional specimen: No. 2997/4468 (PIN) [53]; Korneev and Cate (2005: 10): checklist [120].

 

***Hypnomorphus inventus* Dolin, 1975**

*Hypnomorphus inventus* Dolin, 1975: 57 [51].

Type material. Holotype, sex unknown, exoskeleton, compression fossil, No. 2384/491 (PIN). Four paratypes, sex unknown, exoskeletons, compression fossils, Nos. 2231/15 (Galkino), 2384/450, 2384/479, 2554/697 (Mikhailovka) (PIN).

Fossil deposit/age. Kazakhstan: Karabastau Formation, Karatau, Mikhailovka (type locality), Karatau, Galkino; 166.1–157.3 Ma (Jurassic).

Literature. Dolin (1975: 57): original description [51]; Dolin (1980: 27,30): key, additional specimens: Nos. 2384/498, 2784/1381, 2784/1364 (PIN) [53]; Korneev and Cate (2005: 17): checklist [120].

 

***Hypnomorphus minutus* Dolin, 1975**

*Hypnomorphus minutus* Dolin, 1975: 59 [51].

Type material. Holotype, sex unknown, exoskeleton, compression fossil, No. 2239/1431 (PIN). 10 paratypes, sex unknown, exoskeletons, compression fossils, Nos. 2239/1428, 2239/1429, 2239/1430, 2239/1440 (part + counterpart), 2239/1424, 2554/691, 2554/655, 2554/700, 2066/2318, 2066/3248 (part) + 2066/2744 counterpart) (PIN).

Fossil deposit/age. Kazakhstan: Karabastau Formation, Karatau, Mikhailovka; 164.7–155.7 Ma (Jurassic).

Literature. Dolin (1975: 59): original description [51]; Dolin (1980: 27,30): key, additional specimens: Nos. 2784/1395, 2997/1981, 2997/2004, 2997/4457, 2997/4460 (PIN) [53]; Korneev and Cate (2005: 20): checklist [120].

 

***Hypnomorphus rasnitzyni* Dolin, 1980**

Hypnomorphus rasnitzyni Dolin, 1980: 28 [53].

Type material. Holotype, male, exoskeleton, compression fossil, No. 2784/1362 (part + counterpart) (PIN). Paratype, female, sex unknown, exoskeleton, compression fossil, No. 2997/418 (PIN).

Fossil deposit/age. Kazakhstan: Karabastau Formation, Karatau, Mikhailovka; 164.7–155.7 Ma (Jurassic).

Literature. Dolin (1980: 28): original description [53]; Korneev and Cate (2005: 22): checklist [120]; Dong et al. (2011: 482): remark [214].

Remark. Based on the habitus and shapes of antenna and pronotum, this species most probably belongs to Eucnemidae.

 

***Hypnomorphus rohdendorfi* Dolin, 1975**

*Hypnomorphus rohdendorfi* Dolin, 1975: 56 [51].

*Hypnomorphus rohdendorphi*: Dolin, 1980: 28 [53] [unavailable name, incorrect subsequent spelling not in prevailing usage; [129], Art. 33.3].

Type material. Holotype, male, exoskeleton, compression fossil, No. 2384/457 (part + counterpart) (PIN). Three paratypes, sex unknown, exoskeletons, compression fossils, Nos. 2231/76 (Galkino), 2066/2341, 254/676 (Mikhailovka) (PIN).

Fossil deposit/age. Kazakhstan: Karabastau Formation, Karatau, Mikhailovka (type locality), Karatau, Galkino; 166.1–157.3 Ma (Jurassic).

Literature. Dolin (1975: 56): original description [51]; Dolin (1980: 28,30): key, additional specimens: Nos. 2997/415, 2997/416, 2997/4469 (PIN) [53]; Carpenter (1992: 304): generic catalogue [68]; Korneev and Cate (2005: 10): checklist [120]; Kundrata et al. (2020: 12): generic catalogue [12].

 

- **Genus** ***Lapidiconides* Dolin, 1980**

*Lapidiconides* Dolin, 1980: 43 [53]. Type species: *Lapidiconides excellens* Dolin, 1980: 43 [53]. For more information, see Kundrata et al. [12].

Remark. Species of this genus strongly resemble Eucnemidae in the compact body, with wide prothorax and relatively short elytra, and also Throscidae in almost trapezoidal pronotum. Unfortunately, the main diagnostic characters [99] are either absent or not well visible on the original drawings or photographs [53]. Additionally, *L. innatus* is most probably not congeneric with other two species.

 

***Lapidiconides brevis* Dolin, 1980**

*Lapidiconides brevis* Dolin, 1980: 44 [53].

Type material. Holotype, sex unknown, exoskeleton, compression fossil, No. 2066/3156 (PIN).

Fossil deposit/age. Kazakhstan: Karabastau Formation, Karatau, Mikhailovka; 166.1–157.3 Ma (Jurassic).

Literature. Dolin (1980: 44): original description [53]; Korneev and Cate (2005: 13): checklist [120].

 

***Lapidiconides excellens* Dolin, 1980**

*Lapidiconides excellens* Dolin, 1980: 43 [53].

*Lapidoconides* [sic!] *excellens* Dolin, 1980: 43 [53].

Type material. Holotype, sex unknown, exoskeleton, compression fossil, No. 2066/2453 (PIN).

Fossil deposit/age. Kazakhstan: Karabastau Formation, Karatau, Mikhailovka; 166.1–157.3 Ma (Jurassic).

Literature. Dolin (1980: 43): original description [53]; Carpenter (1992: 304): generic catalogue [68]; Korneev and Cate (2005: 10): checklist [120]; Kundrata et al. (2020: 12): generic catalogue [12].

 

***Lapidiconides innatus* Dolin, 1980**

*Lapidiconides innatus* Dolin, 1980: 44 [53].

Type material. Holotype, sex unknown, exoskeleton, compression fossil, No. 2784/1376 (part) + 2784/1400 (counterpart) (PIN).

Fossil deposit/age. Kazakhstan: Karabastau Formation, Karatau, Mikhailovka; 166.1–157.3 Ma (Jurassic).

Literature. Dolin (1980: 44): original description [53]; Korneev and Cate (2005: 17): checklist [120].

Remark. This species differs considerably from its congeners in the large body size, shape of prosternum and hypomeron, less developed longitudinal sutures on prosternum, and more broadened metacoxal plates. Its systematic placement should be re-evaluated after the study of the type material.

 

- **Genus *Lapidostenus* Dolin, 1980**

*Lapidostenus* Dolin, 1980: 30 [53]. Type species: *Lapidostenus infossus* Dolin, 1980: 31 [53]. For more information, see Kundrata et al. [12].

Remark. This genus needs a revision since some species differ from the type species (and also from each other) in the body proportions, the shape of pronotum, elytra, etc. Most species probably belong to Eucnemidae based on the compact body, with a short and broad thorax, and short elytra. Only *L. tarbinskyi* Dolin, 1980 looks like a typical elaterid. Unfortunately, the main diagnostic characters [99] are usually absent or not well visible on the original drawings or photographs [53].

 

***Lapidostenus infossus* Dolin, 1980**

*Lapidostenus infossus* Dolin, 1980: 31 [53].

*Lapidostenus intossus*: Dolin, 1980: legend of Figure 15 [53] [unavailable name, incorrect original spelling ([129], Art. 19.3); First Reviser ([129], Art. 24.2): Carpenter (1992: 304) [68]].

Type material. Holotype, sex unknown, exoskeleton, compression fossil, No. 2239/1439 (part + counterpart) (PIN). Two paratypes, sex unknown, exoskeletons, compression fossils, Nos. 2066/2431, 2997/2015 (part + counterpart) (PIN).

Fossil deposit. Kazakhstan: Karabastau Formation, Karatau, Mikhailovka; 166.1–157.3 Ma (Jurassic).

Literature. Dolin (1980: 31): original description [53]; Carpenter (1992: 304): generic catalogue [68]; Korneev and Cate (2005: 10): checklist [120]; Kundrata et al. (2020: 12): generic catalogue [12].

 

***Lapidostenus insignis* Dolin, 1980**

*Lapidostenus insignis* Dolin, 1980: 32 [53].

Type material. Holotype, sex unknown, exoskeleton, compression fossil, No. 2784/1379 (PIN).

Fossil deposit. Kazakhstan: Karabastau Formation, Karatau, Mikhailovka; 166.1–157.3 Ma (Jurassic).

Literature. Dolin (1980: 32): original description [53]; Korneev and Cate (2005: 17): checklist [120].

 

***Lapidostenus longicornis* Dolin, 1980**

*Lapidostenus longicornis* Dolin, 1980: 31/32 [53].

*Lapidostenus lognicornis* Dolin, 1980: 32 [53] [unavailable name, incorrect original spelling ([129], Art. 19.3); First Revisers ([129], Art. 24.2): Korneev and Cate (2005: 19) [120]].

Type material. Holotype, sex unknown, exoskeleton, compression fossil, No. 2784/1393 (PIN).

Fossil deposit/age. Kazakhstan: Karabastau Formation, Karatau, Mikhailovka; 166.1–157.3 Ma (Jurassic).

Literature. Dolin (1980: 32): original description [53]; Korneev and Cate (2005: 19): checklist [120].

 

***Lapidostenus scutellaris* Dolin, 1980**

*Lapidostenus scutellaris* Dolin, 1980: 31 [53].

Type material. Holotype, sex unknown, exoskeleton, compression fossil, No. 2066/2909 (PIN). Paratype, sex unknown, exoskeleton, compression fossil, No. 2997/2010 (PIN).

Fossil deposit/age. Kazakhstan: Karabastau Formation, Karatau, Mikhailovka; 166.1–157.3 Ma (Jurassic).

Literature. Dolin (1980: 31): original description [53]; Korneev and Cate (2005: 23): checklist [120].

 

***Lapidostenus tarbinskyi* Dolin, 1980**

*Lapidostenus tarbinskyi* Dolin, 1980: 32 [53].

Type material. Holotype, sex unknown, exoskeleton, compression fossil, No. 2239/1450 (PIN).

Fossil deposit/age. Kazakhstan: Karabastau Formation, Karatau, Mikhailovka; 166.1–157.3 Ma (Jurassic).

Literature. Dolin (1980: 32): original description [53]; Korneev and Cate (2005: 24): checklist [120].

Remark. This species resembles Cardiophorinae in having the strongly arcuate sides of pronotum, with posterior angles short and curved inwards.

 

- **Genus** ***Lithoptychus* Dolin, 1980**

*Lithoptychus* Dolin, 1980: 57 [53]. Type species: *Lithoptychus handlirschi* Dolin, 1980: 57 [53]. For more information, see Kundrata et al. [12].

Remark: This genus might belong to Eucnemidae because its type species, *L. handlirschi*, along with *L. minutus* Dolin, 1980, have a compact body, with a short and broad thorax, and short elytra. Unfortunately, the main diagnostic characters [99] are missing in the original figures [53], and, therefore, the taxonomic decision should be postponed until the type material is studied. Additionally, *L. incertus* Dolin, 1980 has antennae with last three antennomeres enlarged, which is a character present in Eucnemidae rather than Elateridae. *Lithoptychus carinatissimus* Dolin, 1980 externally also resembles Eucnemidae but it has very conspicuous sublateral carina on each side of pronotum.

 

***Lithoptychus carinatissimus* Dolin, 1980**

*Lithoptychus carinatissimus* Dolin, 1980: 59 [53].

Type material. Holotype, sex unknown, exoskeleton, compression fossil, No. 2904/921 (PIN).

Fossil deposit/age. Kazakhstan: Karabastau Formation, Karatau, Mikhailovka; 166.1–157.3 Ma (Jurassic).

Literature. Dolin (1980: 59): original description [53]; Korneev and Cate (2005: 13): checklist [120].

 

***Lithoptychus handlirschi* Dolin, 1980**

*Lithoptychus handlirschi* Dolin, 1980: 57 [53].

*Lithoptychus handlischi*: Dolin, 1980: legend of Figure 60 [53] [unavailable name, incorrect original spelling ([129], Art. 19.3); First Reviser ([129], Art. 24.2): Carpenter (1992: 305) [68]].

Type material. Holotype, sex unknown, exoskeleton, compression fossil, No. 2554/696 (PIN). Paratype, sex unknown, exoskeleton, compression fossil, No. 2997/4439 (PIN).

Fossil deposit/age. Kazakhstan: Karabastau Formation, Karatau, Mikhailovka; 166.1–157.3 Ma (Jurassic).

Literature. Dolin (1980: 57): original description [53]; Carpenter (1992: 305): generic catalogue [68]; Korneev and Cate (2005: 10): checklist [120]; Kundrata et al. (2020: 12): generic catalogue [12].

 

***Lithoptychus incertus* Dolin, 1980**

*Lithoptychus incertus* Dolin, 1980: 58 [53].

Type material. Holotype, sex unknown, exoskeleton, compression fossil, No. 2452/41 (PIN). Three paratypes, sex unknown, exoskeletons, compression fossils, Nos. 2784/1373, 2784/1390, 2784/1398 (Mikhailovka) (PIN).

Fossil deposit/age. Kazakhstan: Karabastau Formation, Karatau, Galkino (type locality), Mikhailovka; 166.1–157.3 Ma (Jurassic).

Literature. Dolin (1980: 58): original description [53]; Korneev and Cate (2005: 16): checklist [120]; Dong and Huang (2011: 1228): morphological remark [81].

 

***Lithoptychus minutus* Dolin, 1980**

*Lithoptychus minutus* Dolin, 1980: 58 [53].

Type material. Holotype, sex unknown, exoskeleton, compression fossil, No. 2239/1461 (PIN). Three paratypes, sex unknown, exoskeletons, compression fossils, Nos. 2239/1495, 2997/426, 2997/427 (PIN).

Fossil deposit/age. Kazakhstan: Karabastau Formation, Karatau, Mikhailovka; 166.1–157.3 Ma (Jurassic).

Literature. Dolin (1980: 58): original description [53]; Korneev and Cate (2005: 20): checklist [120].

 

- **Genus** ***Lithosomus* Dolin, 1980**

*Lithosomus* Dolin, 1980: 46 [53]. Type species: *Lithosomus erosus* Dolin, 1980: 47 [53]. For more information, see Kundrata et al. [12].

Remark. The systematic placement of this genus needs to be re-evaluated since its type species might actually represent Eucnemidae.

 

***Lithosomus erosus* Dolin, 1980**

*Lithosomus erosus* Dolin, 1980: 47 [53].

Type material. Holotype, sex unknown, exoskeleton, compression fossil, No. 2997/1987 (part) + 2997/1995 (counterpart) (PIN). Two paratypes, sex unknown, exoskeletons, compression fossils, Nos. 2997/1989, 2997/1967 (PIN).

Fossil deposit/age. Kazakhstan: Karabastau Formation, Karatau, Mikhailovka; 166.1–157.3 Ma (Jurassic).

Literature. Dolin (1980: 47): original description [53]; Carpenter (1992: 305): generic catalogue [68]; Korneev and Cate (2005: 10): checklist [120]; Kundrata et al. (2020: 12): generic catalogue [12].

Remark. This species probably belongs to Eucnemidae due to its compact body, with a short and broad thorax, short elytra, and antennae with last three antennomeres enlarged. However, since the main diagnostic characters [99] are not well visible in the original figures [53], we prefer to postpone any taxonomic decision until the type material is examined in detail.

 

***Lithosomus longicollis* Dolin, 1980**

*Lithosomus longicollis* Dolin, 1980: 47 [53].

Type material. Holotype, sex unknown, exoskeleton, compression fossil, No. 2784/1363 (PIN).

Fossil deposit/age. Kazakhstan: Karabastau Formation, Karatau, Mikhailovka; 166.1–157.3 Ma (Jurassic).

Literature. Dolin (1980: 47): original description [53]; Korneev and Cate (2005: 19): checklist [120].

Remark. Generic attribution of this species needs re-examination as it strongly differs from the type species of *Lithosomus* in the elongated pronotum, with slightly arcuate sides (pronotum short, broad, strongly campaniform in *L. erosus*), more elongated elytra (elytra rather short in *L. erosus*), more broadened metacoxal plates which are only slightly narrowed outwards (metacoxal plates notably narrowed outwards in *L. erosus*), and larger punctures in elytral striae (small punctures in *L. erosus*).

Although the body proportions of *L. longicollis* somewhat resemble Cardiophorinae or Negastriinae, with almost parallel-sided prosternal sutures and only slightly broadened prosternum being more typical for Cardiophorinae, there are no reliable characters that would point us to the proper systematic placement of this species.

 

- **Genus** ***Necrocoelus* Dolin, 1980**

*Necrocoelus* Dolin, 1980: 59 [53]. Type species: *Necrocoelus aselloides* Dolin, 1980: 59 [53]. For more information, see Kundrata et al. [12].

Remark. This genus might represent Cardiophorinae based on the globose pronotum, with short posterior angles, and a thickened and short prosternal process.

 

***Necrocoelus aselloides* Dolin, 1980**

*Necrocoelus aselloides* Dolin, 1980: 59 [53].

Type material. Holotype, sex unknown, exoskeleton, compression fossil, No. 2066/2520 (part + counterpart) (PIN). Paratype, sex unknown, exoskeleton, compression fossil, No. 2904/909 (PIN).

Fossil deposit/age. Kazakhstan: Karabastau Formation, Karatau, Mikhailovka; 166.1–157.3 Ma (Jurassic).

Literature. Dolin (1980: 59): original description [53]; Carpenter (1992: 305): generic catalogue [68]; Korneev and Cate (2005: 10): checklist [120]; Kundrata et al. (2020: 12): generic catalogue [12].

 

- Genus *Negastrioides* Dolin, 1980

*Negastrioides* Dolin, 1980: 52 [53]. Type species: *Negastrioides tenuis* Dolin, 1980: 52 [53]. For more information, see Kundrata et al. [12].

Remark. Species of this genus resemble Eucnemidae in having a broad prothorax with short pronotal posterior angles and a short prosternal process. Available figures in Dolin [53] also suggest that the type species, *N. tenuis*, along with *N. globicollis* Dolin, 1980, have a pedicel subapically attached to scape, a condition typical for Eucnemidae [99]. Basal antennomeres in remaining two species are absent in figures [53]. Systematic placement of *Negastrioides* needs further investigation.

 

***Negastrioides globicollis* Dolin, 1980**

*Negastrioides globicollis* Dolin, 1980: 53 [53].

Type material. Holotype, sex unknown, exoskeleton, compression fossil, No. 2554/705 (PIN). Three paratypes, sex unknown, exoskeletons, compression fossils, Nos. 2784/1375, 2997/1972, 2997/1993 (PIN).

Fossil deposit/age. Kazakhstan: Karabastau Formation, Karatau, Mikhailovka; 166.1–157.3 Ma (Jurassic).

Literature. Dolin (1980: 53): original description [53]; Korneev and Cate (2005: 15): checklist [120].

 

***Negastrioides tenuicornis* Dolin, 1980**

*Negastrioides tenuicornis* Dolin, 1980: 53 [53].

Type material. Holotype, sex unknown, exoskeleton, compression fossil, No. 2066/2847 (PIN). Three paratypes, sex unknown, exoskeletons, compression fossils, Nos. 2997/1971, 2997/2001, 2997/2006 (PIN).

Fossil deposit/age. Kazakhstan: Karabastau Formation, Karatau, Mikhailovka; 166.1–157.3 Ma (Jurassic).

Literature. Dolin (1980: 53): original description [53]; Korneev and Cate (2005: 24): checklist [120].

 

***Negastrioides tenuis* Dolin, 1980**

*Negastrioides tenuis* Dolin, 1980: 52 [53].

Type material. Holotype? male, exoskeleton, compression fossil, No. 2066/2320 (part + counterpart) (PIN). Three paratypes, sex unknown, exoskeletons, compression fossils, Nos. 2066/2886 (part + counterpart), 2239/1447, 2997/2002 (PIN).

Fossil deposit/age. Kazakhstan: Karabastau Formation, Karatau, Mikhailovka; 166.1–157.3 Ma (Jurassic).

Literature. Dolin (1980: 52): original description [53]; Carpenter (1992: 305): generic catalogue [68]; Korneev and Cate (2005: 10): checklist [120]; Kundrata et al. (2020: 13): generic catalogue [12].

 

***Negastrioides tscherepanovi* Dolin, 1980**

*Negastrioides tscherepanovi* Dolin, 1980: 54 [53].

Type material. Holotype, sex unknown, exoskeleton, compression fossil, No. 2066/2451 (PIN). Two paratypes, sex unknown, exoskeletons, compression fossils, Nos. 2997/424, 2997/4458 (part + counterpart) (PIN).

Fossil deposit/age. Kazakhstan: Karabastau Formation, Karatau, Mikhailovka; 166.1–157.3 Ma (Jurassic).

Literature. Dolin (1980: 54): original description [53]; Korneev and Cate (2005: 24): checklist [120].

 

- **Genus** ***Parahypnomorphus* Dolin, 1980**

*Parahypnomorphus* Dolin, 1980: 33 [53]. Type species: *Parahypnomorphus jurassicus* Dolin, 1980: 33 [53]. For more information, see Kundrata et al. [12].

Remark. This genus might belong to Eucnemidae as all its species have a compact body, with a short and broad thorax, and short elytra. Illustrations of antennae also support this hypothesis [99], especially those of *P. longicornis* Dolin, 1980; however, they should be examined directly on the type material.

 

***Parahypnomorphus jurassicus* Dolin, 1980**

*Parahypnomorphus jurassicus* Dolin, 1980: 33 [53].

Type material. Holotype, sex unknown, exoskeleton, compression fossil, No. 2554/703 (PIN).

Fossil deposit/age. Kazakhstan: Karabastau Formation, Karatau, Mikhailovka; 166.1–157.3 Ma (Jurassic).

Literature. Dolin (1980: 33): original description [53]; Carpenter (1992: 305): generic catalogue [68]; Korneev and Cate (2005: 10): checklist [120]; Kundrata et al. (2020: 13): generic catalogue [12].

 

***Parahypnomorphus longicornis* Dolin, 1980**

*Parahypnomorphus longicornis* Dolin, 1980: 34 [53].

Type material. Holotype, sex unknown, exoskeleton, compression fossil, No. 2239/1442 (PIN). Paratype, sex unknown, exoskeleton, compression fossil, No. 2997/1988 (PIN).

Fossil deposit. Kazakhstan: Karabastau Formation, Karatau, Mikhailovka; 1661.1–157.3 Ma (Jurassic).

Literature. Dolin (1980: 34): original description [53]; Korneev and Cate (2005: 19): checklist [120].

 

***Parahypnomorphus similis* Dolin, 1980**

*Parahypnomorphus similis* Dolin, 1980: 34 [53].

Type material. Holotype, sex unknown, exoskeleton, compression fossil, No. 2997/2013 (PIN).

Fossil deposit. Kazakhstan: Karabastau Formation, Karatau, Mikhailovka; 166.1–157.3 Ma (Jurassic).

Literature. Dolin (1980: 34): original description [53]; Korneev and Cate (2005: 23): checklist [120].

 

- **Genus** ***Platyelater* Dolin, 1980**

*Platyelater* Dolin, 1980: 40 [53]. Type species: *Platyelater reflexicollis* Dolin, 1980: 41 [53]. For more information, see Kundrata et al. [12].

Remark. All species other than the type species, *P. reflexicollis*, resemble Eucnemidae in having a compact body, with a short and broad thorax, and short elytra. Available figures of antennae also support this hypothesis. Additionally, *P. figeratus* Dolin, 1980 shares almost trapezoidal pronotum, with Throscidae. Systematic position of those species should be re-evaluated after study of the type material.

 

***Platyelater figeratus* Dolin, 1980**

*Platyelater figeratus* Dolin, 1980: 42 [53].

Type material. Holotype, sex unknown, exoskeleton, compression fossil, No. 2239/1963 (PIN). Seven paratypes, sex unknown, exoskeletons, compression fossils, Nos. 2239/1408, 2239/1410 (female), 2239/1457, 2066/2461, 2904/920, 2997/1990, 2997/2003 (PIN).

Fossil deposit/age. Kazakhstan: Karabastau Formation, Karatau, Mikhailovka; 166.1–157.3 Ma (Jurassic).

Literature. Dolin (1980: 42): original description [53]; Korneev and Cate (2005: 15): checklist [120].

Remark. This species notably differs from its congeners, especially in the body proportions and the shape of metacoxal plates.

 

***Platyelater quiescentus* Dolin, 1980**

*Platyelater quiescentus* Dolin, 1980: 42 [53].

Type material. Holotype, sex unknown, exoskeleton, compression fossil, No. 2997/416 (PIN). Two paratypes, sex unknown, exoskeletons, compression fossils, Nos. 2784/1402, 2997/414 (part + counterpart) (PIN).

Fossil deposit/age. Kazakhstan: Karabastau Formation, Karatau, Mikhailovka; 166.1–157.3 Ma (Jurassic).

Literature. Dolin (1980: 42): original description [53]; Korneev and Cate (2005: 22): checklist [120].

 

***Platyelater reflexicollis* Dolin, 1980**

*Platyelater reflexicollis* Dolin, 1980: 41 [53].

Type material. Holotype, sex unknown, exoskeleton, compression fossil, No. 2904/900 (part + counterpart) (PIN). Six paratypes, sex unknown, exoskeletons, compression fossils, Nos. 2384/485, 2554/683, 2784/1387, 2904/906 (part + counterpart), 2904/914, 2997/423 (PIN).

Fossil deposit/age. Kazakhstan: Karabastau Formation, Karatau, Mikhailovka; 166.1–157.3 Ma (Jurassic).

Literature. Dolin (1980: 41): original description [53]; Carpenter (1992: 305): generic catalogue [68]; Korneev and Cate (2005: 10): checklist [120]; Dong et al. (2011: 482): remark [214]; Kundrata et al. (2020: 13): generic catalogue [12].

 

***Platyelater sukatschevae* Dolin, 1980**

*Platyelater sukatschevae* Dolin, 1980: 41 [53].

Type material. Holotype, sex unknown, exoskeleton, compression fossil, No. 2997/2026 (PIN).

Fossil deposit/age. Kazakhstan: Karabastau Formation, Karatau, Mikhailovka; 166.1–157.3 Ma (Jurassic).

Literature. Dolin (1980: 41): original description [53]; Korneev and Cate (2005: 24): checklist [120].

#### 3.9.3. Tribe Pollostelaterini Alekseev, 2011

Pollostelaterini Alekseev, 2011: 424 [75]. Type genus: *Pollostelater* Alekseev, 2011: 424 [75]. For more information, see Kundrata et al. [12].

 

- **Genus *Pollostelater* Alekseev, 2011**

*Pollostelater* Alekseev, 2011: 424 [75]. Type species: *Pollostelater baissensis* Alekseev, 2011: 424 [75]. For more information, see Kundrata et al. [12].

Remark. Based on the original description and available images [75], we cannot exclude the possibility that this genus belongs to Eucnemidae due to the compact body, with a short and broad thorax. The longitudinal sutures on prosternum are obviously situated close to pronotosternal sutures, so they might be just deeply furrowed pronotosternal sutures as in some Eucnemidae. The presence of a sublateral carina on pronotum, which is a character typical for Eateridae rather than Eucnemidae, should be re-evaluated on the type material.

 

***Pollostelater baissensis* Alekseev, 2011**

*Pollostelater baissensis* Alekseev, 2011: 424 [75].

Type material. Holotype, sex unknown, exoskeleton, impression, No. 3064/7100 (PIN).

Fossil deposit/age. Russia: Buryatia, Zaza Formation, Baissa; 125.0–113.0 Ma (Cretaceous).

Literature. Alekseev (2011: 424): original description [75]; Kundrata et al. (2020: 13): generic catalogue [12].

#### 3.9.4. Tribe Protagrypnini Dolin, 1973

Protagrypnini Dolin, 1973: 74 [50]. Type genus: *Protagrypnus* Dolin, 1973: 75 [50]. For more information, see Bouchard et al. [110] and Kundrata et al. [12].

 

- **Genus *Acheonus* Dolin, 1980**

*Acheonus* Dolin, 1980: 20 [53]. Type species: *Acheonus abbreviatus* Dolin, 1980: 21 [53]. For more information, see Kundrata et al. [12].

Remark. Species of this genus externally resemble Negastriinae but their placement in Elateridae should be rather confirmed by a study of type material. There are inconsistencies in original descriptions and corresponding images in Dolin [53] regarding the presence or absence of sublateral carinae in pronotal posterior angles.

 

***Acheonus abbreviatus* Dolin, 1980**

*Acheonus abbreviatus* Dolin, 1980: 21 [53].

Type material. Holotype, sex unknown, exoskeleton, compression fossil, No. 2384/462 (PIN). Paratype, sex unknown, exoskeleton, compression fossil, No. 2384/462 [in the original description, holotype and paratype have the same number, which is probably an error] (PIN).

Fossil deposit/age. Kazakhstan: Karabastau Formation, Karatau, Mikhailovka; 166.1–157.3 Ma (Jurassic).

Literature. Dolin (1980: 21): original description [53]; Carpenter (1992: 304): generic catalogue [68]; Korneev and Cate (2005: 9): checklist [120]; Schimmel and Tarnawski (2012: 265): remark [132]; Kundrata et al. (2020: 13): generic catalogue [12].

 

***Acheonus gracilis* Dolin, 1980**

*Acheonus gracilis* Dolin, 1980: 21 [53].

Type material. Holotype, sex unknown, exoskeleton, compression fossil, No. 2784/1404 (PIN).

Fossil deposit/age. Kazakhstan: Karabastau Formation, Karatau, Mikhailovka; 166.1–157.3 Ma (Jurassic).

Literature. Dolin (1980: 21): original description [53]; Schimmel and Tarnawski (2012: 265): remark [132].

 

***Acheonus minutissimus* Dolin, 1980**

*Acheonus minutissimus* Dolin, 1980: 21 [53].

Type material. Holotype, sex unknown, exoskeleton, compression fossil, No. 2997/1992 (PIN).

Fossil deposit/age. Kazakhstan: Karabastau Formation, Karatau, Mikhailovka; 166.1–157.3 Ma (Jurassic).

Literature. Dolin (1980: 21): original description [53]; Korneev and Cate (2005: 20): checklist [120]; Schimmel and Tarnawski (2012: 265): remark [132].

 

- **Genus *Clavelater* Dong and Huang, 2011**

*Clavelater* Dong and Huang, 2011: 1225 [81]. Type species: *Clavelater ningchengensis* Dong and Huang, 2011: 1226 [81]. For more information, see Kundrata et al. [12].

Remark. Muona et al. [99] suggested that this genus may represent an unknown lineage of Eucnemidae rather than Elateridae. They based their conclusions on the available literature and figures but the type material should be studied to solve the placement of *Clavelater* within Elateroidea. Nevertheless, we agree with the conclusions made by Muona et al. [99].

 

***Clavelater ningchengensis* Dong and Huang, 2011**

*Clavelater ningchengensis* Dong and Huang, 2011: 1226 [81].

Type material. Holotype, sex unknown, exoskeleton, compression fossil, No. 151,836 (NIGP).

Fossil deposit/age. China: Inner Mongolia, Ningcheng County, Jiulongshan Formation, Daohugou; 166.1–157.3 Ma (Jurassic).

Literature. Dong and Huang (2011: 1226): original description [81]; Yu et al. (2019: 384): remark [89]; Kundrata et al. (2020: 14): generic catalogue [12]; Muona et al. (2020: 11): revision [99].

 

- **Genus *Koreagrypnus* Sohn and Nam, 2019**

*Koreagrypnus* Sohn and Nam in Sohn et al., 2019: 6 [76]. Type species: *Koreagrypnus jinju* Sohn and Nam in Sohn et al., 2019: 6 [76]. For more information, see Kundrata et al. [12].

 

***Koreagrypnus jinju* Sohn and Nam, 2019**

*Koreagrypnus jinju* Sohn and Nam in Sohn et al., 2019: 6 [76].

Type material. Holotype, sex unknown, exoskeleton, compression fossil, GNUE-I-2013002 and GNUE-I-2013002c (GNUE).

Fossil deposit/age. South Korea: Gyeongsangnamdo Province, Jinju Formation, Jeongchon Mountain, Jeongchon City, Jinju; 113.0–100.5 Ma (Cretaceous).

Literature. Sohn et al. (2019: 6): original description [76]; Kundrata et al. (2020: 14): generic catalogue [12].

 

- **Genus *Lithocoelus* Dolin, 1975**

*Lithocoelus* Dolin, 1975: 53 [51]. Type species: *Lithocoelus detrusus* Dolin, 1975: 53 [51]. For more information, see Kundrata et al. [12].

Remark. This genus might belong to Eucnemidae as both its species have a compact body, with a short and broad thorax, and relatively short elytra [51]. The type material needs to be checked for characters distinguishing (with more or less certainty) Elateridae from Eucnemidae [99].

 

***Lithocoelus detrusus* Dolin, 1975**

*Lithocoelus detrusus* Dolin, 1975: 53 [51].

Type material. Holotype, sex unknown, exoskeleton, compression fossil, No. 2384/484 (PIN).

Fossil deposit/age. Kazakhstan: Karabastau Formation, Karatau, Mikhailovka; 166.1–157.3 Ma (Jurassic).

Literature. Dolin (1975: 53): original description [51]; Dolin (1980: 20): remark [53]; Carpenter (1992: 305): generic catalogue [68]; Korneev and Cate (2005: 10): checklist [120]; Kundrata et al. (2020: 14): generic catalogue [12].

 

***Lithocoelus karatavicus* Dolin, 1975**

*Lithocoelus karatavicus* Dolin, 1975: 54 [51].

Type material. Holotype, sex unknown, exoskeleton, compression fossil, No. 2239/1425 (part + counterpart) (PIN). Paratype, sex unknown, exoskeleton, compression fossil, No. 2335/82 (PIN).

Fossil deposit/age. Kazakhstan: Karabastau Formation, Karatau, Mikhailovka; 166.1–157.3 Ma (Jurassic).

Literature. Dolin (1975: 54): original description [51]; Dolin (1980: 20): remark [53]; Korneev and Cate (2005: 18): checklist [120].

 

- **Genus *Lithomerus* Dolin, 1980**

*Lithomerus* Dolin, 1980: 23 [53]. Type species: *Lithomerus cockerelli* Dolin, 1980: 23 [53]. For more information, see Kundrata et al. [12].

Remark. This genus needs a revision since some species differ from the type species (and also from each other) in the body proportions, the shape of pronotum, elytra, etc. *Lithomerus cockerelli* (type species) and *L. brevicollis* Dolin, 1980 are probably correctly assigned to Elateridae but other species from the Palearctic deposits currently assigned to this genus might belong to Eucnemidae as they have a compact body, with a short and broad thorax, and short elytra. *Lithomerus wunda* Martin, 2010 from Australia is most probably a member of Throscidae [99].

 

***Lithomerus brachycollis* Dolin, 1980**

*Lithomerus brachycollis* Dolin, 1980: 23 [53].

Type material. Holotype, sex unknown, exoskeleton, compression fossil, No. 2997/4463 (PIN).

Fossil deposit/age. Kazakhstan: Karabastau Formation, Karatau, Mikhailovka; 166.1–157.3 Ma (Jurassic).

Literature. Dolin (1980: 23): original description [53]; Martin (2010: 934): remark [73].

Remark. This species was mentioned by Dolin [53] only in the key on page 23 but the author obviously forgot to include the usual description which was otherwise available for all other new species in that paper. The holotype was figured in Figure 9 (drawing) and Plate I, Figure 3 (photograph), its collection number was mentioned in the figure legends, and the depository was specified in the introductory part of the paper [53]. The character states mentioned in the key on page 23 in Dolin [53] fulfill the requirements of Art. 13.1.1 [129] and, therefore, *L. brachycollis* is an available name.

 

***Lithomerus brevicollis* Dolin, 1980**

*Lithomerus brevicollis* Dolin, 1980: 24 [53].

Type material. Holotype, sex unknown, exoskeleton, compression fossil, No. 2904/908 (PIN).

Fossil deposit/age. Kazakhstan: Karabastau Formation, Karatau, Mikhailovka; 166.1–157.3 Ma (Jurassic).

Literature. Dolin (1980: 24): original description [53]; Dolin and Nel (2002: 341): remark [82]; Korneev and Cate (2005: 13): checklist [120]; Martin (2010: 934): remark [73]; Schimmel and Tarnawski (2012: 265): remark [132].

 

***Lithomerus buyssoni* Dolin and Nel, 2002**

*Lithomerus buyssoni* Dolin and Nel, 2002: 341 [82].

Type material. Holotype, sex unknown, exoskeleton, compression fossil, MNHN-LP-R.55231 (MNHN).

Fossil deposit/age. China: Liaoning Province, Yixian Formation, Beipiao City, Chaomidian Village; 125.45–122.46 Ma (Cretaceous).

Literature. Dolin and Nel (2002: 341): original description [82]; Kirejtshuk et al. (2010: 792): checklist [87]; Martin (2010: 934): remark [73]; Muona et al. (2020: 11): revision [99].

Remark. Muona et al. [99] suggested that this species might belong either to Elateridae: Protagrypninae or Eucnemidae: Palaeoxeninae but they did not study the holotype.

 

***Lithomerus cockerelli* Dolin, 1980**

*Lithomerus cockerelli* Dolin, 1980: 23 [53].

Type material. Holotype, sex unknown, exoskeleton, compression fossil, No. 2384/454 (PIN). Seven paratypes, sex unknown, exoskeletons, compression fossils, Nos. 2384/460, 2384/492, 2784/1389, 2554/685, 2997/1977, 2997/1984, 2784/1366 (part + counterpart) (PIN).

Fossil deposit/age. Kazakhstan: Karabastau Formation, Karatau, Mikhailovka; 166.1–157.3 Ma (Jurassic).

Literature. Dolin (1980: 23): original description [53]; Carpenter (1992: 305): generic catalogue [68]; Dolin and Nel (2002: 341): remark [82]; Korneev and Cate (2005: 10): checklist [120]; Martin (2010: 932): remark [73]; Schimmel and Tarnawski (2012: 265): remark [132]; Yu et al. (2019: 382): remark [89]; Kundrata et al. (2020: 14): generic catalogue [12].

 

***Lithomerus contiguus* Dolin, 1980**

*Lithomerus contiguus* Dolin, 1980: 24 [53].

*Lithomerus contiguous*: Schimmel and Tarnawski, 2012: 265 [132] [unavailable name, incorrect subsequent spelling not in prevailing usage; [129], Art. 33.3].

Type material. Holotype, sex unknown, exoskeleton, compression fossil, No. 2066/2978 (PIN). Paratype, sex unknown, exoskeleton, compression fossil, No. 2997/2019 (PIN).

Fossil deposit/age. Kazakhstan: Karabastau Formation, Karatau, Mikhailovka; 166.1–157.3 Ma (Jurassic).

Literature. Dolin (1980: 24): original description [53]; Dolin and Nel (2002: 341): remark [82]; Korneev and Cate (2005: 14): checklist [120]; Martin (2010: 934): remark [73]; Schimmel and Tarnawski (2012: 265): remark [132].

 

***Lithomerus longulus* Dolin, 1980**

*Lithomerus longulus* Dolin, 1980: 24 [53].

*Lithomerus longulatus*: Schimmel and Tarnawski, 2012: 265 [132] [unavailable name, incorrect subsequent spelling not in prevailing usage; [129], Art. 33.3].

Type material. Holotype, sex unknown, exoskeleton, compression fossil, No. 2066/3018 (PIN).

Fossil deposit/age. Kazakhstan: Karabastau Formation, Karatau, Mikhailovka; 166.1–157.3 Ma (Jurassic).

Literature. Dolin (1980: 24): original description [53]; Dolin and Nel (2002: 341): remark [82]; Korneev and Cate (2005: 19): checklist [120]; Martin (2010: 934): remark [73]; Schimmel and Tarnawski (2012: 265): remark [132].

 

***Lithomerus wunda* Martin, 2010**

*Lithomerus wunda* Martin, 2010: 932 [73].

Type material. Holotype, sex unknown, exoskeleton, compression fossil, WAM 08.179 (WAM). One paratype, sex unknown, exoskeleton, compression fossil, WAM 08.180 (WAM).

Fossil deposit/age. Australia: Cattamarra Coal Measures Formation, Mintaja M1 site; 182.7–174.1 Ma (Jurassic).

Literature. Martin (2010: 932): original description [73].

Remark. This species is obviously not conspecific with any other *Lithomerus* species. Based on the figures in the original description [73], Muona et al. [99] suggested that it should be placed within Throscidae based on the body size and the shape of antenna resting in the antennal groove. They postponed its transfer to Throscidae until the holotype is examined.

 

- **Genus *Megalithomerus* Sohn and Nam, 2019**

*Megalithomerus* Sohn and Nam in Sohn et al., 2019: 3 [76]. Type species: *Megalithomerus magohalmii* Sohn and Nam in Sohn et al., 2019: 3 [76]. For more information, see Kundrata et al. [12].

 

***Megalithomerus magohalmii* Sohn and Nam, 2019**

*Megalithomerus magohalmii* Sohn and Nam in Sohn et al. 2019: 4 [76].

Type material. Holotype, sex unknown, exoskeleton, compression fossil, GNUE-I-2013001 and GNUE-I-2013001c (GNUE).

Fossil deposit/age. South Korea: Gyeongsangnamdo Province, Jinju Formation, Jeongchon Mountain, Jeongchon City; 113.0–100.5 Ma (Cretaceous).

Literature. Sohn and Nam (2019: 4): original description [76]; Kundrata et al. (2020: 14): generic catalogue [12].

 

- **Genus *Micragrypnites* Dolin, 1973**

*Micragrypnites* Dolin, 1973: 76 [50]. Type species: *Micragrypnites issykiensis* Dolin, 1973: 77 [50]. For more information, see Kundrata et al. [12].

Remark. We cannot exclude the possibility that this genus might belong to Eucnemidae based on a short and broad prothorax with short posterior pronotal angles. Unfortunately, the main diagnostic characters [99] are either absent or not well visible on the images [53] and so the type material should be examined to confirm the placement of *Micragrypnites* in Elateridae.

 

***Micragrypnites issykiensis* Dolin, 1973**

*Micragrypnites issykiensis* Dolin, 1973: 77 [50].

Type material. Holotype, sex unknown, exoskeleton, impression, No. 371/1648 (PIN).

Fossil deposit/age. Kyrgyzstan: Dzhil Formation, Ak-Bulak-Say, Sogjuta, Issyk-Kul; 201.3–190.8 Ma (Jurassic).

Literature. Dolin (1973: 77): original description [50]; Carpenter (1992: 305): generic catalogue [68]; Korneev and Cate (2005: 10): check list [120]; Dong et al. (2011: 482): remark [214]; Kundrata et al. (2020: 14): generic catalogue [12].

 

- **Genus *Paragrypnites* Dolin, 1980**

*Paragrypnites* Dolin, 1980: 22 [53]. Type species: *Paragrypnites jagemanni* Dolin, 1980: 22 [53]. For more information, see Kundrata et al. [12].

 

***Paragrypnites jagemanni* Dolin, 1980**

*Paragrypnites jagemanni* Dolin, 1980: 22 [53].

Type material. Holotype, sex unknown, exoskeleton, compression fossil, No. 2997/1980 (PIN).

Fossil deposit/age. Kazakhstan: Karabastau Formation, Karatau, Mikhailovka; 166.1–157.3 Ma (Jurassic).

Literature. Dolin (1980: 22): original description [53]; Carpenter (1992: 305): generic catalogue [68]; Korneev and Cate (2005: 10): checklist [120]; Schimmel and Tarnawski (2012: 265): remark [132]; Kundrata et al. (2020: 15): generic catalogue [12].

 

- **Genus** ***Paraprotagrypnus* Chang, Zhao and Ren, 2009**

*Paraprotagrypnus* Chang, Zhao and Ren, 2009: 1433 [79]. Type species: *Paraprotagrypnus superbus* Chang, Zhao and Ren, 2009: 1434 [79]. For more information, see Kundrata et al. [12].

Remark. Muona et al. [99] examined this genus and concluded that it should be retained in Elateridae rather than transferred to Eucnemidae or Throscidae. They supported their conclusion by several morphological features, including antennomere II being attached apically to antennomere I. However, on the figures provided by Chang et al. [79], the antennomere II is attached somewhat subapically to antennomere I, and what is more, three apical antennomeres are enlarged, which is a character usually found in other clicking elateroids than Elateridae. The shape of prothorax, with broad and stout pronotum and anteriorly truncate prosternum, is also pointing to Eucnemidae. Another interesting character in this genus is a presence of sublateral carinae on pronotum, described and figured in Chang et al. [79]. These usually run from posterior angles more or less subparallel with sides. Muona et al. [99], however, wrote that “pronotal hind angles seemed to lack carina”. Here, we follow Muona et al. [99], who studied the type material of the only species classified in *Paraprotagrypnus*, and retain this genus in Protagrypnini as originally proposed.

 

***Paraprotagrypnus superbus* Chang, Zhao and Ren, 2009**

*Paraprotagrypnus superbus* Chang, Zhao and Ren, 2009: 1434 [79].

Type material. Holotype, sex unknown, exoskeleton, compression fossil, CNU-COL-NN2006878 (CNU). One paratype, male, exoskeleton, compression fossil, CNU-COL-NN2006879PC (CNU).

Fossil deposit/age. China: Inner Mongolia, Ningcheng County, Jiulongshan Formation, Shantou Township Daohugou; 166.1–157.3 Ma (Jurassic).

Literature. Chang et al. (2009: 1434): original description [79]; Kirejtshuk et al. (2010: 791): checklist [87]; Dong and Huang (2011: 1228): remark [81]; Yu et al. (2019: 382): remark [89]; Kundrata et al. (2020: 15): generic catalogue [12]; Muona et al. (2020: 10): revision [99].

 

- **Genus *Protagrypnus* Dolin, 1973**

*Protagrypnus* Dolin, 1973: 75 [50]. Type species: *Protagrypnus exoletus* Dolin, 1973: 75 [50]. For more information, see Kundrata et al. [12].

Remarks. In the original description of the genus, Dolin erroneously used the name *Praelaterium* Dolin, 1973 and vice versa, *Protagrypnus* was used in the original description of *Praelaterium*. However, species names were used correctly in both cases ([50], pp. 75, 78). Species in *Protagrypnus* do not seem to be congeneric. They differ considerably in body size, shape of pronotum and elytra, and shape and proportions of prosternum and hypomeron. We cannot exclude the possibility that the type species belongs to Eucnemidae.

 

***Protagrypnus exoletus* Dolin, 1973**

*Protagrypnus exoletus* Dolin, 1973: 75 [50].

Type material. Holotype, sex unknown, exoskeleton, impression, No. 358/785 (PIN).

Fossil deposit/age. Kyrgyzstan: Dzhil Formation, Sogyuty, Issyk-Kul; 201.3–190.8 Ma (Jurassic).

Literature. Dolin (1973: 75): original description [50]; Carpenter (1992: 305): generic catalogue [68]; Korneev and Cate (2005: 10): checklist [120]; Dong and Huang (2009: 104): remark [80]; Chang et al. (2009: 10): remark [78]; Kundrata et al. (2020: 15): generic catalogue [12].

 

***Protagrypnus robustus* Chang, Kirejtshuk and Ren, 2009**

*Protagrypnus robustus* Chang, Kirejtshuk and Ren, 2009: 11 [78].

Type material. Holotype, male, exoskeleton, impression, CNU-COL-NN2006843 (CNU). Two paratypes, exoskeletons, impressions, males, CNU-COL-NN2006875 and CNU-COL-NN2007869 (CNU).

Fossil deposit/age. China: Inner Mongolia, Ningcheng County, Jiulongshan Formation, Daohugou;166.1–157.3 Ma (Jurassic).

Literature. Chang et al. (2009: 11): original description [78]; Kirejtshuk et al. (2010: 791): checklist [87]; Dong and Huang (2011: 1227): remark [81]; Schimmel and Tarnawski (2012: 265): remark [132]; Yu et al. (2019: 381): remark [89]; Muona et al. (2020: 10): revision [99].

Remark. This species was examined by Muona et al. [99], who concluded that it should retain in Elateridae: Protagrypninae.

 

- **Genus *Sinolithomerus* Dong and Huang, 2009**

*Sinolithomerus* Dong and Huang, 2009: 103 [80]. Type species: *Sinolithomerus dolini* Dong and Huang, 2009: 104 [80]. For more information, see Kundrata et al. [12].

Remark. This genus might belong to Eucnemidae due to its relatively short and broad thorax, and short posterior angles of pronotum. Unfortunately, the main diagnostic characters [99] are either absent or not well visible on the original figures [53].

 

***Sinolithomerus dolini* Dong and Huang, 2009**

*Sinolithomerus dolini* Dong and Huang, 2009: 104 [80].

Type material. Holotype, sex unknown, exoskeleton, compression fossil, No. 149,367 (NIGP).

Fossil deposit/age. China: Inner Mongolia, Ningcheng County, Haifanggou Formation, Beipiao City, Jiangjiagou village; 166.1–157.3 Ma (Jurassic).

Literature. Dong and Huang (2009: 104): original description [80]; Yu et al. (2019: 383): remark [89]; Kundrata et al. (2020: 15): generic catalogue [12]; Muona et al. (2020: 11): remark [99].

### 3.10. Elateridae Incertae Sedis

 

- **Genus *Adocetus* Scudder, 1900**

*Adocetus* Scudder, 1900: 97 [157]. Type species: *Adocetus buprestoides* Scudder, 1900: 97 [157]. For more information, see Kundrata et al. [12].

Remark. Based on the figure in original description, this genus might belong to Buprestidae rather than Elateridae. Therefore, the type material should be examined to confirm the proper family placement of *Adocetus*.

 

***Adocetus buprestoides* Scudder, 1900**

*Adocetus buprestoides* Scudder, 1900: 97 [157].

Type material. Holotype, sex unknown, compression fossil (Newberry coll., Columbia University, NY, USA; based on the original description).

Fossil deposit/age. USA: Wyoming, Green River Formation, Bluffs by Twin Creek; 55.8–50.3 Ma (Eocene).

Literature. Scudder (1900: 97): original description [157]; Handlirsch (1907: 747): catalogue [127]; Carpenter (1992: 304): generic catalogue [68]; Kundrata et al. (2020: 16): generic catalogue [12].

 

- **Genus *Artinama* Lin, 1986**

*Artinama* Lin, 1986: 72 [55]. Type species: *Artinama qinghuoensis* Lin, 1986: 73 [55]. For more information, see Ponomarenko et al. [88] and Kundrata et al. [12].

Remark. Muona et al. [99] suggested that this genus belongs to Elateridae. Brief description of *Artinama* does not enable its placement even to a subfamily level. According to the images in Dong and Huang [81], *Artinama* resembles members of tribe Agrypnini (note that the drawing in that publication does not fully correspond with the photograph).

 

***Artinama qinghuoensis* Lin, 1986**

*Artinama qinghuoensis* Lin, 1986: 73 [55].

*Artinama qinghuanensis*: Muona et al., 2020: 9 [99] [unavailable name, incorrect subsequent spelling not in prevailing usage; [129], Art. 33.3].

Type material. Holotype, sex unknown, exoskeleton, impression, No. 70,064 (NIGP).

Fossil deposit/age. China: Zaoshang Formation, Liuyang City, KHG 100, Shijiaba section, Wenjiashi; 199.3–190.8 Ma (Jurassic).

Literature. Lin (1986: 73): original description [55]; Dong et al. (2011: 483): remark [214]; Dong and Huang (2011: 1228): remark [81]; Ponomarenko et al. (2012: 480): revision [88]; Kundrata et al. (2020: 16): generic catalogue [12]; Muona et al. (2020: 9): revision [99].

 

- **Genus *Bilineariselater* Chang and Ren, 2008**

*Bilineariselater* Chang and Ren, 2008: 237 [84]. Type species: *Bilineariselater foveatus* Chang and Ren, 2008: 237 [84]. For more information, see Kundrata et al. [12].

Remark. This genus superficially resembles Selatosomini in having the frontal carina obsolete, the antenna weakly serrate, with elongated antennomere III, the pronotum rather broad, arcuate at sides and sinuate near hind angles, the posterior angles of pronotum moderately long, with sublateral carina, the prosternal lobe well developed, and elytra more or less ellipsoidal.

 

***Bilineariselater foveatus* Chang and Ren, 2008**

*Bilineariselater foveatus* Chang and Ren, 2008: 237 [84].

Type material. Holotype, male, exoskeleton, compression fossil, CNU-C-LB2006801 (CNU).

Fossil deposit/age. China: Liaoning Province, Shangyan County, Beipiao City, Yixian Formation, Huangbanjigou, near Chaomidian Village; 125.45–122.46 Ma (Cretaceous).

Literature. Chang and Ren (2008: 237): original description [84]; Kirejtshuk et al. (2010: 792): checklist [87]; Dong and Huang (2011: 1225): checklist [81]; Yu et al. (2019: 382): remark [89]; Kundrata et al. (2020: 16): generic catalogue [12]; Muona et al. (2020: 9): revision [99].

 

- **Genus *Cretoelaterium* Alekseev, 2008**

*Cretoelaterium* Alekseev, 2008: 56 [74]. Type species: *Cretoelaterium kazanovense* Alekseev, 2008: 57 [74]. For more information, see Kundrata et al. [12].

Remark. We cannot exclude the possibility that this genus might belong to Eucnemidae.

 

***Cretoelaterium kazanovense* Alekseev, 2008**

*Cretoelaterium kazanovense* Alekseev, 2008: 57 [74].

Type material. Holotype, sex unknown, exoskeleton, impression, No. 3693/1 (PIN).

Fossil deposit/age. Russia: Mirsanovo Formation, Chita Region, Shilka District, left bank of the Shilka river, Kazanovo railway station; 129.4–125.0 Ma (Cretaceous).

Literature. Alekseev (2008: 57): original description [74]; Kundrata et al. (2020: 16): generic catalogue [12].

 

- **Genus *Cryptagriotes* Wickham, 1916**

*Cryptagriotes* Wickham, 1916: 512 [28]. Type species: *Cryptagriotes minusculus* Wickham, 1916: 512 [28]. For more information, see Kundrata et al. [12].

Remark. Wickham [28] suggested that the body form of this genus is similar to *Cryptohypnus* Eschscholtz, 1830 (=*Hypolithus* Eschscholtz, 1829; Dendrometrinae: Hypnoidini) and the metacoxal plates are similar to those of *Agriotes* Eschscholtz, 1829 (Elaterinae: Agriotini). Based on the body size and proportions, and the more or less narrow prosternum with pronotosternal sutures almost parallel sided, this genus might belong to Cardiophorinae. However, its potential affinities to Eucnemidae should also be taken into account.

 

***Cryptagriotes minusculus* Wickham, 1916**

*Cryptagriotes minusculus* Wickham, 1916: 512 [28].

*Cryptagriotes minisculus*: Carpenter, 1992: 304 [68] [unavailable name, incorrect subsequent spelling not in prevailing usage; [129], Art. 33.3].

Type material. Holotype, sex unknown, exoskeleton, compression fossil, MCZ 2749 (=8653 in Scudder coll.) (MCZ).

Fossil deposit/age. USA: Colorado, Florissant Formation, Florissant; 37.2–33.9 Ma (Eocene).

Literature. Wickham (1916: 512): original description [28]; Wickham (1920: 354): catalogue [29]; Hyslop (1921: 637): generic catalogue [111]; Carpenter (1992: 304): generic catalogue [68]; Kundrata et al. (2020: 7): catalogue [12].

 

- **Genus *Cryptocoelus* Dolin and Nel, 2002**

*Cryptocoelus* Dolin and Nel, 2002: 342 [82]. Type species: *Cryptocoelus buffoni* Dolin and Nel, 2002: 342 [82]. For more information, see Chang et al. [83] and Kundrata et al. [12].

Remark. Species in this genus superficially resemble Selatosomini in the frontal carina obsolete, the antenna more or less weakly serrate, the pronotum rather broad, arcuate at sides and sinuate near posterior angles, the posterior angles of pronotum moderately long, with sublateral carina, and elytra more or less ellipsoidal.

 

***Cryptocoelus baissensis* Alekseev, 2011**

*Cryptocoelus baissensis* Alekseev, 2011: 428 [75].

Type material. Holotype, sex unknown, exoskeleton, impression, No. 1989/2636 (PIN).

Fossil deposit/age. Russia: Buryatia, Zaza Formation, Baissa; 125.0–113.0 Ma (Cretaceous).

Literature. Alekseev (2011: 428): original description [75].

 

***Cryptocoelus buffoni* Dolin and Nel, 2002**

*Cryptocoelus buffoni* Dolin and Nel, 2002: 342 [82].

Type material. Holotype, sex unknown, exoskeleton, compression fossil, MNHN-LP-R.55227 (MNHN). Three paratypes, sex unknown, exoskeletons, compression fossils, MNHN-LP-R.55228, MNHN-LP-R.55229, MNHN-LP-R.55230 (MNHN).

Fossil deposit/age. China: Liaoning Province, Beipiao City, Yixian Formation, Chaomidian Village; 125.45–122.46 Ma (Cretaceous).

Literature. Dolin and Nel (2002: 342): original description [82]; Korneev and Cate (2005: 13): checklist [120]; Chang et al. (2007: 1248): remark [83]; Kirejtshuk et al. (2010: 792): checklist [87]; Alekseev (2011: 424): checklist [75]; Dong and Huang (2011: 1225): checklist [81]; Kundrata et al. (2020: 16): generic catalogue [12]; Muona et al. (2020: 9): revision [99].

Remark. Based on Figures 3–10 in Dolin and Nel [82], there are more species included in the type series of *C. buffoni*. The specimen in Figure 10 has a well developed prosternal lobe, while specimens in Figures 4, 6 and 8 have anterior portion of prosternum truncate. Additionally, the shape of elytra is also different among the figured specimens.

 

***Cryptocoelus dolini* Alekseev, 2011**

*Cryptocoelus dolini* Alekseev, 2011: 430 [75].

Type material. Holotype, sex unknown, exoskeleton, impression, No. 4210/6420 (PIN).

Fossil deposit/age. Russia: Buryatia, Zaza Formation, Baissa; 125.0–113.0 Ma (Cretaceous).

Literature. Alekseev (2011: 430): original description [75].

 

***Cryptocoelus gianteus* Chang, Ren and Shih, 2007**

*Cryptocoelus gianteus* Chang, Ren and Shih, 2007: 1245 [83].

*Cryptocoelus giganteus*: Kirejtshuk et al., 2010: 792 [87] [unavailable name, incorrect subsequent spelling not in prevailing usage; [129], Art. 33.3].

Type material. Holotype, sex unknown, exoskeleton, compression fossil, CNU-C-LB2006851-1, CNU-C-LB2006851-2 (CNU).

Fossil deposit/age. China: Liaoning Province, Shangyuan County, Beipiao City, Yixian Formation, Huangbanjigou, near Chaomidian Village; 125.45–122.46 Ma (Cretaceous).

Literature. Chang et al. (2007: 1245): original description [83]; Kirejtshuk et al. (2010: 792): checklist [87]; Alekseev (2011: 425): checklist [75]; Dong and Huang (2011: 1225): checklist [81]; Muona et al. (2020: 9): revision [99].

 

***Cryptocoelus lukashevichae* Alekseev, 2011**

*Cryptocoelus lukashevichae* Alekseev, 2011: 428 [75].

Type material. Holotype, sex unknown, exoskeleton, impression, No. 3064/7102 (PIN).

Fossil deposit/age. Russia: Buryatia, Zaza Formation, Baissa; 125.0–113.0 Ma (Cretaceous).

Literature. Alekseev (2011: 428): original description [75].

 

***Cryptocoelus major* Dolin and Nel, 2002**

*Cryptocoelus major* Dolin and Nel, 2002: 343 [82].

*Crytocoleus* [sic!] *major*: Yu et al., 2019: 381 [89].

Type material. Holotype, sex unknown, exoskeleton, compression fossil, MNHN-LP-R.55226 (MNHN).

Fossil deposit/age. China: China: Liaoning Province, Shangyuan County, Beipiao City, Yixian Formation, Huangbanjigou, near Chaomidian Village; 125.45–122.46 Ma (Cretaceous).

Literature. Dolin and Nel (2002: 343): original description [82]; Korneev and Cate (2005: 10): checklist [120]; Chang et al. (2007: 1245): remark [83]; Chang et al. (2010: 873): remark [86]; Kirejtshuk et al. (2010: 787, 792): checklist [87]; Alekseev (2011: 424): checklist [75]; Dong and Huang (2011: 1225): checklist [81]; Yu et al. (2019: 381): remark [89]; Kundrata et al. (2020: 16): generic catalogue [12]; Muona et al. (2020: 9): revision [99].

 

***Cryptocoelus shcherbakovi* Alekseev, 2011**

*Cryptocoelus shcherbakovi* Alekseev, 2011: 428 [75].

Type material. Holotype, sex unknown, exoskeleton, impression, No. 4210/6417 (PIN).

Fossil deposit/age. Russia: Buryatia, Zaza Formation, Baissa; 125.0–113.0 Ma (Cretaceous).

Literature. Alekseev (2011: 428): original description [75].

 

***Cryptocoelus sinitshenkovae* Alekseev, 2011**

*Cryptocoelus sinitshenkovae* Alekseev, 2011: 427 [75].

Type material. Holotype, sex unknown, exoskeleton, No. 3002/2 (PIN).

Fossil deposit/age. Russia: Buryatia, Zaza Formation, Romanovka; 125.0–113.0 Ma (Cretaceous).

Literature. Alekseev (2011: 427): original description [75].

 

- **Genus *Curtelater* Chang and Ren, 2008**

*Curtelater* Chang and Ren, 2008: 238 [84]. Type species: *Curtelater wui* Chang and Ren, 2008: 239 [84]. For more information, see Kundrata et al. [12].

Remark. Muona et al. [99] examined this genus and confirmed that it belongs to Elateridae. However, based on the available morphological evidence, its position remains unclear, although it superficially resembles Selatosomini in several aspects including the morphology of head, antennae, and prothorax [84].

 

***Curtelater wui* Chang and Ren, 2008**

*Curtelater wui* Chang and Ren, 2008: 239 [84].

Type material. Holotype, female, exoskeleton, compression fossil, CNU-C-LB2006830 (CNU).

Fossil deposit/age. China: Liaoning Province, Shangyuan County, Beipiao City, Yixian Formation, Huangbanjigou, near Chaomidian Village; 125.45–122.46 Ma (Cretaceous).

Literature. Chang and Ren (2008: 239): original description [84]; Kirejtshuk et al. (2010: 792): checklist [87]; Dong and Huang (2011: 1225): checklist [81]; Yu et al. (2019: 382): remark [89]; Kundrata et al. (2020: 17): generic catalogue [12]; Muona et al. (2020: 9): revision [99].

 

- **Genus *Elateridium* Tillyard, 1918**

*Elateridium* Tillyard, 1918: 751 [38]. Replacement name for *Elaterites* Tillyard, 1916: 41 [37]. Type species: *Elaterites wianamattense* Tillyard, 1916: 41 [37]. For more information, see Kundrata et al. [12].

Remark. The description of the type species of *Elateridium* is based on an isolated elytron, which makes the systematic placement of this genus rather problematic. Both species described by Dunstan [39] which are listed below should be excluded from Elateridae.

 

***Elateridium subulatum* (Dunstan, 1923)**

*Elaterites subulatus* Dunstan, 1923: 44 [39].

*Elateridium subulatum*: Jell, 2004: 76 [215].

Type material. Holotype, sex unknown, elytron, compression fossil, Nr. 263a,b (part + counterpart) (QM; ex Geological Survey of Queensland).

Fossil deposit/age. Australia: Queensland, Blackstone Formation (Ipswich Coal Measures Group), Denmark Hill Insect Bed; 228.0–208.5 Ma (Triassic).

Literature. Dunstan (1923: 44): original description [39]; Handlirsch (1938: 13): catalogue [212]; Jell (2004: 76): catalogue [215]; Martins-Neto et al. (2006: 602): remark [71]; Martin (2010: 936): remark [73].

Remark. According to the drawing in the original description [39], it seems doubtful that this species belongs to Elateridae since the shape and structure of elytron are not typically elaterid like.

 

***Elateridium transversum* (Dunstan, 1923)**

*Elaterites trans* versus Dunstan, 1923: 45 [39].

*Elateridium transversum*: Handlirsch, 1938: 13 [212].

Type material. Holotype, sex unknown, elytron, compression fossil, Nr. 159a, A (part+counterpart) (QM; ex Geological Survey of Queensland).

Fossil deposit/age. Australia: Queensland, Blackstone Formation (Ipswich Coal Measures Group), Denmark Hill Insect Bed; 228.0–208.5 Ma (Triassic).

Literature. Dunstan (1923: 45): original description [39]; Handlirsch (1938: 13): catalogue [212]; Jell (2004: 76): remark [215].

Remark. According to the drawing in the original description [39], it seems doubtful that this species belongs to Elateridae since the shape and structure of elytron are not typically elaterid like.

 

***Elateridium wianamattense* (Tillyard, 1916)**

*Elaterites wianamattensis* Tillyard, 1916: 41 [37].

Elateridium wianamattense: Jell, 2004: 76 [215].

Type material. Holotype, sex unknown, elytron, impression, Nr. 130 (QM; ex Geological Survey of Queensland).

Fossil deposit. Australia: Ashfield Shales Formation, Carrington Brick Company’s clay pit, St. Peter’s; 247.2–242.0 Ma (Triassic).

Literature. Tillyard (1916: 41): original description [37]; Tillyard (1918: 751): nomenclatural remark [38]; Handlirsch (1938: 158): remark [212]; Carpenter (1992: 304): generic catalogue [68]; Jell (2004: 76): remark [215]; Kundrata et al. (2020: 17): generic catalogue [12].

 

- **Genus *Elaterites* Heer, 1847**

*Elaterites* Heer, 1847: 141 [14]. Type species: *Elaterites lavateri* Heer, 1847: 141 [14]. For more information, see Kundrata et al. [12].

Remark. This genus is obviously an assemblage of unrelated species of uncertain position. It includes taxa which authors were not possible to accommodate to any other genus in Elateridae [14,34]. Although it could be considered a “collective group” [129], some authors treated *Elaterites* as a genus with its own type species [127] and we follow this concept. The type species might represent Dendrometrinae based on the drawings in the original description [14].

 

***Elaterites amissus* Heer, 1847**

*Elaterites amissus* Heer, 1847: 142 [14].

Type material. Holotype, sex unknown, elytron, compression fossil (type depository unknown).

Fossil deposit/age. Switzerland: Greith coal mine, Hohenrhone; 28.4–23.03 Ma (Oligocene).

Literature. Heer (1847: 142): original description [14]; Giebel (1852: 651): catalogue [126]; Giebel (1856: 94): revision, redescription [16]; Scudder (1891: 518): catalogue [24]; Handlirsch (1907: 747): catalogue [127].

Remark. The description of *E. amissus* was based on an isolated elytron, which makes the generic attribution of this species rather problematic.

 

***Elaterites bruchi* Cockerell, 1926**

*Elaterites bruchi* Cockerell, 1926: 320 [35].

Type material. Holotype, sex unknown, elytron, compression fossil (BMNH).

Fossil deposit/age. Argentina: Margas Verdes Formation, Station 2; Sunchal; 66.0–56.0 Ma (Paleocene).

Literature. Cockerell (1926: 320): original description [35]; Cockerell (1936: 1): checklist [216].

Remark. The description of *E. bruchi* was based on an isolated elytron, which makes the generic attribution of this species rather problematic.

 

***Elaterites dicrepidioides* Deichmüller, 1881**

*Elaterites dicrepidioides* Deichmüller, 1881: 308 [21].

Type material. Unknown number of type specimens, probably only one, sex unknown, compression fossil (type depository unknown).

Fossil deposit/age. Czech Republic: Kučlín (u Bíliny); 37.2–33.9 Ma (Eocene).

Literature. Deichmüller (1881: 308): original description [21]; Scudder (1891: 518): catalogue [24]; Handlirsch (1907: 748): catalogue [127].

 

***Elaterites laconoides* Cockerell, 1920**

*Elaterites laconoides* Cockerell, 1920: 457 [34].

Type material. Holotype, sex unknown, elytron, compression fossil, No. 18,998 (BMNH).

Fossil deposit/age. United Kingdom: Poole Formation, Bournemouth; 47.8–41.3 Ma (Eocene).

Literature. Cockerell (1920: 457): original description [34].

Remark. The description of *E. laconoides* was based on an isolated elytron, which makes the generic attribution of this species rather problematic.

 

***Elaterites lavateri* Heer, 1847**

*Elaterites lavateri* Heer, 1847: 141 [14].

Type material. Holotype, sex unknown, compression fossil (ETH).

Fossil deposit/age. Germany: Upper Freshwater-Molasse Formation, Upper Öhningen beds, Öhningen; 12.7–11.608 Ma (Miocene).

Literature. Heer (1847: 141): original description [14]; Giebel (1852: 651): catalogue [126]; Giebel (1856: 94): revision, redescription [16]; Scudder (1891: 518): catalogue [24]; Handlirsch (1907: 747): catalogue [127]; Tillyard (1918: 751): remark [38]; Cockerell (1920: 457): remark [34]; Kundrata et al. (2020: 17): generic catalogue [12].

Remark. This species might represent Dendrometrinae based on the drawings in the original description [14]. It looks especially similar to genera such as *Elathous* Reitter, 1890, *Limonius* and *Pheletes* Kiesenwetter, 1858.

 

***Elaterites longus* Haupt, 1956**

*Elaterites longus* Haupt, 1956: 48 [147].

Type material. Holotype, sex unknown, exoskeleton, compression fossil, G55/53 (GPIUH).

Fossil deposit/age. Germany: Geiseltal; 47.8–41.3 Ma (Eocene).

Literature. Haupt (1956: 48): original description [147].

Remark. The description of *E. longus* was based only on the characters of elytra and abdomen, which makes the generic attribution of this species rather problematic.

 

***Elaterites microstictus* Cockerell, 1926**

*Elaterites microstictus* Cockerell, 1926: 320 [35].

Type material. Holotype, sex unknown, elytron, compression fossil (part + counterpart) (BMNH, YPM).

Fossil deposit/age. Argentina: Margas Verdes Formation, Station 2; Sunchal; 66.0–56.0 Ma (Paleocene).

Literature. Cockerell (1926: 320): original description [35]; Cockerell (1936: 1): checklist [216].

Remark. The description of *E. microstictus* was based on an isolated elytron, which makes the generic attribution of this species rather problematic.

 

***Elaterites murchisoni* (Giebel, 1856)**

*Elaterium murchisoni* Giebel, 1856: 93 [16].

*Elaterites murchisoni*: Cockerell, 1920: 456 [34].

Type material. Holotype, sex unknown, elytron, compression fossil, No. 18,996 (BMNH).

Fossil deposit/age. United Kingdom: Poole Formation, Dorset, Creech, between Corfe and Wareham; 47.8–41.3 Ma (Eocene).

Literature. Giebel (1856: 93): original description [16]; Scudder (1891: 518): catalogue [24]; Handlirsch (1907: 748): catalogue [127]; Cockerell (1920: 456): revision [34].

Remark. The description of *E. murchisoni* was based on an isolated elytron, which makes the generic attribution of this species rather problematic. It was treated as a member of Buprestidae by Westwood [15].

 

***Elaterites obsoletus* Heer, 1847**

*Elaterites obsoletus* Heer, 1847: 142 [14].

Type material. Holotype, sex unknown, compression fossil, No. 7969 (ETH).

Fossil deposit/age. Germany: Upper Freshwater-Molasse Formation, Upper Öhningen; 12.7–11.608 Ma (Miocene).

Literature. Heer (1847: 142): original description [14]; Giebel (1852: 651): catalogue [126]; Giebel (1856: 94): revision, redescription [16]; Scudder (1891: 518): catalogue [24]; Handlirsch (1907: 747): catalogue [127].

 

***Elaterites palaeophilus* Cockerell, 1920**

*Elaterites palaeophilus* Cockerell, 1920: 458 [34].

Type material. Holotype, sex unknown, elytron, compression fossil, No. 1467 (BMNH).

Fossil deposit/age. United Kingdom: Lambeth Group, Peckham; 56.0–47.8 Ma (Eocene).

Literature. Cockerell (1920: 458): original description [34].

Remark. The description of *E. palaeophilus* was based on an isolated elytron, which makes the generic attribution of this species rather problematic.

 

***Elaterites perditulus* Cockerell, 1920**

*Elaterites perditulus* Cockerell, 1920: 457 [34].

Type material. Holotype, sex unknown, elytron, compression fossil, No. 10,418 (BMNH).

Fossil deposit/age. United Kingdom: Poole Formation, Dorset, Corfe, Isle of Purbeck; 47.8–41.3 Ma (Eocene).

Literature. Cockerell (1920: 457): original description [34].

Remark. The description of *E. perditulus* was based on an isolated elytron, which makes the generic attribution of this species rather problematic.

 

***Elaterites sculptilis* Cockerell, 1920**

*Elaterites sculptilis* Cockerell, 1920: 458 [34].

*Elater sculptilis*: Birket-Smith, 1977: 20 [154].

Type material. Holotype, sex unknown, elytron, compression fossil, No. 10420, 10,422 (two impressions of the same specimen [34]) (BMNH).

Fossil deposit/age. United Kingdom: Poole Formation, Dorset, Corfe, Isle of Purbeck; 47.8–41.3 Ma (Eocene).

Literature. Cockerell (1920: 458): original description [34]; Birket-Smith (1977: 20): taxonomic remark [154].

Remark. Remark. The description of *E. sculptilis* was based on an isolated elytron, which makes the generic attribution of this species rather problematic. Birket-Smith [154] suggested that this species is presumably congeneric with *Semiotus ehrenswaerdi* but the size of both elytra differs significantly.

 

- **Genus *Elaterium* Westwood, 1854**

*Elaterium* Westwood, 1854: 387/393 [15]. Type species: *Elaterium pronaeus* Westwood, 1854: 387/393 [15]. For more information, see Kundrata et al. [12].

Remark. The description of the type species of *Elaterium* is based on a part of an isolated elytron, which makes the systematic placement of this genus rather problematic. A species described by Dunstan [39] is most probably not congeneric with *E. pronaeus*.

 

***Elaterium bipunctatum* Dunstan, 1923**

*Elaterium bipunctatum* Dunstan, 1923: 47 [39].

Type material. Holotype, sex unknown, elytra, compression fossil, No. 292 (QM; ex Geological Survey of Queensland).

Fossil deposit/age. Australia: Blackstone Formation (Ipswich Coal Measures Group), Denmark Hill Insect Bed; 228.0–208.5 Ma (Triassic).

Literature. Dunstan (1923: 47): original description [39]; Handlirsch (1938: 14): catalogue [212]; Jell (2004: 76): remark [215].

Remark. According to the description and drawing in the original publication [39], elytron of this species bears strong costae which are usually not present in Elateridae. The placement of this species not only in *Elaterium* but even in the family Elateridae needs further examination.

 

***Elaterium pronaeus* Westwood, 1854**

*Elaterium pronaeus* Westwood, 1854: 393 [15].

Type material. Holotype, sex unknown, compression fossil (BMNH).

Fossil deposit/age. United Kingdom: Lulworth Formation, Durlston Bay, Lower Purbeck, Swanage; 145.0–140.2 Ma (Cretaceous).

Literature. Westwood (1854: 393): original description [15]; Giebel (1856: 92): revision, redescription [16]; Scudder (1891: 205): catalogue [24]; Handlirsch (1906: 553): catalogue [26]; Cockerell (1920: 456): catalogue [34]; Coram and Jepson (2012: 60): catalogue [217]; Jell (2004: 76): remark [215]; Kundrata et al. (2020: 17): generic catalogue [12].

 

- **Genus *Gripecolous* Lin, 1986**

*Gripecolous* Lin, 1986: 80 [55]. Type species: *Gripecolous enallus* Lin, 1986: 80 [55]. For more information, see Kundrata et al. [12].

Remark. Muona et al. [99] suggested that this genus belongs either to Elateridae: Protagrypninae or to Eucnemidae. According to the images in Dong and Huang [81], *Gripecolous* resembles members of tribe Agrypnini, especially *Agrypnus* (note that drawing in that publication does not fully correspond with the photograph).

 

***Gripecolous enallus* Lin, 1986**

*Gripecolous enallus* Lin, 1986: 80 [55].

Type material. Holotype, female (see Muona et al. [99]), exoskeleton, impression, No. 70,073 (NIGP).

Fossil deposit/age. China: Shiti Formation, KHG 201, Xiwan coal mine, Hezhou City; 170.3–168.3 Ma (Jurassic).

Literature. Lin (1986: 80): original description [55]; Dong et al. (2011: 482): revision [214]; Dong and Huang (2011: 1228): remark [81]; Ponomarenko et al. (2012: 482): revision [88]; Kundrata et al. (2020: 18): generic catalogue [12]; Muona et al. (2020: 11): revision [99].

 

- **Genus *Ludiophanes* Wickham, 1916**

*Ludiophanes* Wickham, 1916: 522 [28]. Type species: *Ludiophanes haydeni* Wickham, 1916: 522 [28]. For more information, see Kundrata et al. [12].

 

***Ludiophanes haydeni* Wickham, 1916**

*Ludiophanes haydeni* Wickham, 1916: 522 [28].

*Ludiophanes hayden*: Carpenter, 1992: 305 [68] [unavailable name, incorrect subsequent spelling not in prevailing usage; [129], Art. 33.3].

Type material. Holotype, sex unknown, compression fossil, No. 90,386 (part + counterpart) (USNM).

Fossil deposit/age. USA: Colorado, Florissant Formation, Wilson Ranch, Florissant; 37.2–33.9 Ma (Eocene).

Literature. Wickham (1916: 522): original description [28]; Wickham (1920: 354): catalogue [29]; Carpenter (1992: 305): generic catalogue [68]; Kundrata et al. (2020: 18): generic catalogue [12].

 

- **Genus *Mercata* Lin, 1986**

*Mercata* Lin, 1986: 79 [55]. Type species: *Mercata festira* Lin, 1986: 79 [55]. For more information, see Kundrata et al. [12].

Remark. Chang et al. [77] placed *Mercata* in Cerophytidae but this was not followed by subsequent authors [12,88,89,218]. Ponomarenko et al. [88] placed this genus in Elateridae: Protagrypninae.

 

***Mercata festira* Lin, 1986**

*Mercata festira* Lin, 1986: 79 [55].

Type material. Holotype, sex unknown, exoskeleton, impression, No. 70,072 (NIGP).

Fossil deposit/age. China: Shiti Formation, KHG 201, Xiwan coal mine, Hezhou City; 170.3–168.3 Ma (Jurassic).

Literature. Lin (1986: 79): original description [55]; Chang et al. (2011: 33): remark [77]; Dong et al. (2011: 486): revision [214]; Ponomarenko et al. (2012: 481): revision [88]; Kundrata et al. (2020: 18): generic catalogue [12]; Muona et al. (2020: 11): remark [99].

 

- **Genus *Mionelater* Becker, 1963**

*Mionelater* Becker, 1963: 125 [46]. Type species: *Mionelater planatus* Becker, 1963: 126 [46]. For more information, see Douglas [108] and Kundrata et al. [12].

*Minonelater* Schimmel, 2005: 27 [91] [unavailable name, incorrect subsequent spelling not in prevailing usage; [129], Art. 33.3].

Remark. Becker [46] placed this genus in Cardiophorinae. However, Douglas [108] suggested that this genus might belong to Dendrometrinae or another subfamily based on the serrate antennae, large eyes, shelf-like supra-antennal carina, elongate posterior angles of pronotum, and open mesocoxal cavities. Additionally, *Mionelater* has a pronotum more elongated than in typical Cardiophorinae, and almost not arcuate at sides, and according to the description [46], the prosternal process in this genus is long and rather narrow, while in Cardiophorinae, it is rather short and thick. The combination of characters, such as small body size, shape of supra-antennal carina, pronotum rather large compared to elytra, and elongate posterior angles of pronotum, suggests that *Mionelater* might be related to Hypnoidini. However, we keep it tentatively without a subfamilial assignment until further, more detailed, study is conducted.

 

***Mionelater planatus* Becker, 1963**

*Mionelater planatus* Becker, 1963: 126 [46].

Type material. Holotype, probably male, exoskeleton, amber inclusion, No. 12,734 (UCMP).

Fossil deposit/age. Mexico: Simojovel region, Mexican (Chiapas) amber; 23.03–15.97 Ma (Miocene).

Literature. Becker (1963: 126): original description [46]; Spahr (1981: 49): catalogue [49]; Keilbach (1982: 247): catalogue [133]; Zaragoza Caballero (1990: 147): remark [61]; Carpenter (1992: 305): generic catalogue [68]; Schimmel (2005: 27): remark [91]; Solórzano Kraemer (2007: 119): catalogue [90]; Schimmel and Tarnawski (2010: 363): remark [131]; Schimmel and Tarnawski (2012: 265): remark [132]; Kundrata et al. (2020: 6): generic catalogue [12]; Kundrata et al. (2020: 8): remark [94].

 

- **Genus *Ovivagina* Zhang, 1997**

*Ovivagina* Zhang, 1997: 71 [56]. Type species: *Ovivagina longa* Zhang, 1997: 72 [56]. For more information, see Kundrata et al. [12].

Remark. The placement of this genus in Elateridae is uncertain and needs further research [78,81,219].

 

***Ovivagina longa* Zhang, 1997**

*Ovivagina longa* Zhang, 1997: 72 [56].

Type material. Holotype, sex unknown, exoskeleton, impression, 93-NA-3/K7,8 (?NIGP).

Fossil deposit/age. China: Badaowan Formation, Xinjiang, Shawan County, Nan’anchihai; 201.3–190.8 Ma (Jurassic).

Literature. Zhang (1997: 72): original description [56]; Yan and Zhang (2010: 451): remark [219]; Dong and Huang (2011: 1228): remark [81]; Kundrata et al. (2020: 18): generic catalogue [12]; Muona et al. (2020: 11): remark [99].

 

- **Genus *Paralithomerus* Chang, Zhang and Ren, 2008**

*Paralithomerus* Chang, Zhang and Ren, 2008: 55 [85]. Type species: *Paralithomerus exquisitus* Chang, Zhang and Ren, 2008: 55 [85]. For more information, see Kundrata et al. [12].

Remark. This genus was considered Protagrypninae *incertae sedis* [12] but Muona et al. [99] placed it to Elateridae *incertae sedis*.

 

***Paralithomerus exquisitus* Chang, Zhang and Ren, 2008**

*Paralithomerus exquisitus* Chang, Zhang and Ren, 2008: 55 [85].

*Paralithomerus exquisitius*: Muona et al., 2020: 10 [99] [unavailable name, incorrect subsequent spelling not in prevailing usage; [129], Art. 33.3].

Type material. Holotype, sex unknown, exoskeleton, impression, CNU-C-LB2006874-1, CNU-C-LB2006874-2 (CNU).

Fossil deposit/age. China: Liaoning Province, Shangyuan County, Beipiao City, Yixian Formation, Huangbanjigou, near Chaomidian Village; 125.45–122.46 Ma (Cretaceous).

Literature. Chang et al. (2008: 55): original description [85]; Kirejtshuk et al. (2010: 792): checklist [87]; Dong and Huang (2011: 1225): checklist [81]; Yu et al. (2019: 382): remark [89]; Kundrata et al. (2020: 15): generic catalogue [12]; Muona et al. (2020: 10): revision [99].

 

***Paralithomerus parallelus* Chang, Zhang and Ren, 2008**

*Paralithomerus parallelus* Chang, Zhang and Ren, 2008: 58 [85].

Type material. Holotype, sex unknown, exoskeleton, impression, CNU-C-LB2006872 (CNU).

Fossil deposit. China: Liaoning Province, Shangyuan County, Beipiao City, Yixian Formation, Huangbanjigou, near Chaomidian Village; 125.45–122.46 Ma (Cretaceous).

Literature. Chang et al. (2008: 58): original description [85]; Kirejtshuk et al. (2010: 792): checklist [87]; Dong and Huang (2011: 1225): checklist [81]; Muona et al. (2020: 10): revision [99].

 

- **Genus *Protocardiophorus* Dolin, 1976**

*Protocardiophorus* Dolin, 1976: 71 [52]. Type species: *Protocardiophorus ancestralis* Dolin, 1976: 73 [52]. For more information, see Douglas [108] and Kundrata et al. [12].

*Photocardiophorus*: Dolin, 1980: legend to Figure 84 [53] [unavailable name, incorrect subsequent spelling not in prevailing usage; [129], Art. 33.3].

Remark. Species of this genus do not seem to be congeneric. What is more, the type material should be studied in order to confirm that they belong to Elateridae and not to other clicking elateroid lineages, especially Eucnemidae.

 

***Protocardiophorus ancestralis* Dolin, 1976**

*Protocardiophorus ancestralis* Dolin, 1976: 73 [52].

Type material. Holotype, sex unknown, exoskeleton, compression fossil, No. 2066/2571 (PIN).

Fossil deposit/age. Kazakhstan: Karabastau Formation, Karatau, Mikhailovka; 166.1–157.3 Ma (Jurassic).

Literature. Dolin (1976: 73): original description [52]; Dolin (1980: 78): key, additional specimen No. 2997/1973 [53]; Carpenter (1992: 305): generic catalogue [68]; Korneev and Cate (2005: 10): checklist [120]; Kundrata et al. (2020: 19): generic catalogue [12].

 

***Protocardiophorus jurassicus* Dolin, 1980**

*Protocardiophorus jurassicus* Dolin, 1980: 78 [53].

*Photocardiophorus* [sic!] *jurassicus*: Dolin, 1980: legend to Figure 84 [53].

Type material. Holotype, sex unknown, exoskeleton, compression fossil, No. 2997/2020 (PIN).

Fossil deposit/age. Kazakhstan: Karabastau Formation, Karatau, Mikhailovka; 166.1–157.3 Ma (Jurassic).

Literature. Dolin (1980: 78): original description [53]; Korneev and Cate (2005: 17): checklist [120].

Remark. *Protocardiophorus jurassicus* is morphologically similar to the species of *Idiomerus* which were transferred to Cerophytidae [77,218].

 

- **Genus Pseudocardiophorites Dolin, 1976**

*Pseudocardiophorites* Dolin, 1976: 73 [52]. Type species: *Pseudocardiophorites fragilis* Dolin, 1976: 73 [52]. For more information, see Douglas [108] and Kundrata et al. [12].

Remark. This genus needs a revision since some species differ from the type species (and also from each other) in the body proportions, the shape and structure of thorax, etc. We cannot exclude the possibility that at least some species currently classified in this genus might belong to Eucnemidae.

 

***Pseudocardiophorites angustatus* Dolin, 1980**

*Pseudocardiophorites angustatus* Dolin, 1980: 80 [53].

Type material. Holotype, sex unknown, exoskeleton, compression fossil, No. 2997/2019 (PIN).

Fossil deposit/age. Kazakhstan: Karabastau Formation, Karatau, Mikhailovka; 166.1–157.3 Ma (Jurassic).

Literature. Dolin (1980: 80): original description [53]; Korneev and Cate (2005: 11): checklist [120].

 

***Pseudocardiophorites fragilis* Dolin, 1976**

*Pseudocardiophorites fragilis* Dolin, 1976: 73 [52].

*Cardiophorites* [sic!] *fragilis*: Dolin, 1976: 72 [52] (figure legend).

Type material. Holotype, sex unknown, exoskeleton, compression fossil, No. 2554/688 (PIN).

Fossil deposit/age. Kazakhstan: Karabastau Formation, Karatau, Mikhailovka; 166.1–157.3 Ma (Jurassic).

Literature. Dolin (1976: 73): original description [52]; Dolin (1980: 79): revision [53]; Carpenter (1992: 305): generic catalogue [68]; Korneev and Cate (2005: 10): checklist [120]; Kundrata et al. (2020: 19): generic catalogue [12].

 

***Pseudocardiophorites hayeki* Dolin, 1976**

*Pseudocardiophorites hayeki* Dolin, 1976: 73 [52].

*Cardiophorites* [sic!] *hayeki*: Dolin, 1976: 72 [52] (figure legend).

*Pseudocardiophorites hayekae*: Dolin, 1980: 80 [53] [unavailable name, incorrect subsequent spelling not in prevailing usage; [129], Art. 33.4].

Type material. Holotype, sex unknown, exoskeleton, compression fossil, No. 2066/2930 (PIN).

Fossil deposit/age. Kazakhstan: Karabastau Formation, Karatau, Mikhailovka; 166.1–157.3 Ma (Jurassic).

Literature. Dolin (1976: 73): original description [52]; Dolin (1980: 80): key [53]; Korneev and Cate (2005: 16): checklist [120].

 

***Pseudocardiophorites infractus* Dolin, 1976**

*Pseudocardiophorites infractus* Dolin, 1976: 74 [52].

*Cardiophorites* [sic!] *infractus* Dolin, 1976: 72 [52] (figure legend).

Type material. Holotype, sex unknown, exoskeleton, compression fossil, No. 2239/1468 (PIN).

Fossil deposit/age. Kazakhstan: Karabastau Formation, Karatau, Mikhailovka; 166.1–157.3 Ma (Jurassic).

Literature. Dolin (1976: 74): original description [52]; Dolin (1980: 80): key [53]; Korneev and Cate (2005: 17): checklist [120].

 

***Pseudocardiophorites quadricollis* Dolin, 1976**

*Pseudocardiophorites quadricollis* Dolin, 1976: 74 [52].

Type material. Holotype, sex unknown, exoskeleton, compression fossil, No. 2239/1422 (PIN). Paratype, sex unknown, exoskeleton, compression fossil, No. 2239/1435 (PIN).

Fossil deposit/age. Kazakhstan: Karabastau Formation, Karatau, Mikhailovka (PIN collection 2239); 166.1–157.3 Ma (Jurassic).

Literature. Dolin (1976: 74): original description [52]; Dolin (1980: 80): key [53]; Korneev and Cate (2005: 22): checklist [120].

 

- **Genus *Silicernius* Heyden, 1859**

*Silicernius* Heyden, 1859: 6 [220]. Type species: *Silicernius spectabilis* Heyden, 1859: 6 [220]. For more information, see Kundrata et al. [12].

Remark. This genus might be related to Oxynopterini based on the body proportions and shapes of pronotum and elytra.

 

***Silicernius spectabilis* Heyden, 1859**

*Silicernius spectabilis* Heyden, 1859: 6 [220].

Type material. Holotype, sex unknown, compression fossil (GPIBO).

Fossil deposit/age. Germany: Rott Formation; 28.4–23.03 Ma (Oligocene).

Literature. Heyden (1859: 6): original description [220]; Scudder (1885: 797): catalogue [22]; Scudder (1891: 580): catalogue [24]; Handlirsch (1907: 747): catalogue [127]; Hyslop (1921: 669): generic catalogue [111]; Kundrata et al. (2020: 19): generic catalogue [12].

Remark. This species resembles Oxynopterini in the broadened and almost roundly campaniform pronotum, with moderately long posterior angles, and elytron notably elongated and attenuate to apex.

 

- **Genus *Sinoelaterium* Ping, 1928**

*Sinoelaterium* Ping, 1928: 22 [40]. Type species: *Sinoelaterium melanocolor* Ping, 1928: 23 [40]. For more information, see Kundrata et al. [12].

Remark. This genus needs a thorough re-examination as it is not clear whether it belongs to Elateridae [12,68]. Some authors suggested that it might belong to Artematopodidae [221,222], but it also resembles Cerophytidae by the habitus, shape of antennae, and structure of head and thorax.

 

***Sinoelaterium melanocolor* Ping, 1928**

*Sinoelaterium melanocolor* Ping, 1928: 23 [40].

*Sinoelaterium melanovolor*: Carpenter, 1992: 305 [68] [unavailable name, incorrect subsequent spelling not in prevailing usage; [129], Art. 33.3].

Type material. Holotype, sex unknown, exoskeleton, compression fossil, No. 2132 (W. H. Wong coll., Geological Survey of China [40]).

Fossil deposit/age. China: Liaoning Province, Yixian Formation, Beipiao City, Locality 228; 125.45–122.46 Ma (Cretaceous).

Literature. Ping (1928: 23): original description [40]; Handlirsch (1938: 167, 169): catalogue [212]; Carpenter (1992: 305): generic catalogue [68]; Dolin and Nel (2002: 345): remark [82]; Dong and Huang (2009: 102): remark [80]; Dong and Huang (2011: 1227): remark [81]; Kundrata et al. (2020: 19): generic catalogue [12]; Muona et al. (2020: 11): remark [99].

 

- **Genus *Tetraraphes* Iablokoff-Khnzorian, 1961**

*Tetraraphes* Iablokoff-Khnzorian, 1961: 95 [47]. Type species: *Tetraraphes ebersini* Iablokoff-Khnzorian, 1961: 96 [47]. For more information, see Douglas [108] and Kundrata et al. [12].

 

***Tetraraphes ebersini* Iablokoff-Khnzorian, 1961**

*Tetraraphes ebersini* Iablokoff-Khnzorian, 1961: 96 [47].

Type material. Holotype, sex unknown, exoskeleton, amber inclusion, No. 364/712 (PIN).

Fossil deposit/age. Baltic amber; 38.0–33.9 Ma (Eocene).

Literature. Iablokoff-Khnzorian (1961: 96): original description [47]; Larsson (1978: 153): catalogue [48]; Spahr (1981: 49): catalogue [49]; Keilbach (1982: 247): catalogue [133]; Carpenter (1992: 305): generic catalogue [68]; Alekseev (2013: 7): checklist [92]; Chang et al. (2010: 867): remark [86]; Kundrata et al. (2020: 19): generic catalogue [12].

 

- **Genus *Turonelater* Alekseev, 2011**

*Turonelater* Alekseev, 2011: 430 [75]. Type species: *Turonelater giganteus* Alekseev, 2011: 430 [75]. For more information, see Alekseev [75] and Kundrata et al. [12].

Remark. This genus probably belongs to Dendrometrinae based on the morphology of prothorax.

 

***Turonelater giganteus* Alekseev, 2011**

*Turonelater giganteus* Alekseev, 2011: 430 [75].

Type material. Holotype, sex unknown, impression, No. 2383/252 (PIN).

Fossil deposit/age. Kazakhstan: Kzylorda region, Pond mudstone, Kzyl-Zhar; 93.9–89.8 Ma (Cretaceous).

Literature. Alekseev (2011: 430): original description [75]; Kundrata et al. (2020: 20): generic catalogue [12].

Remark. *Turonelater giganteus* has a broad pronotum, with arcuate sides, and moderately long and carinate posterior angles. Such pronotum can be found in the widely delimited Dendrometrinae, especially within Prosternini, Selatosomini, Dimini and Oxynopterini. This species might actually belong to Oxynopterini based on the large body size, the pronotum deeply arcuated anteriorly, and the prosternal lobe seemingly weakly developed.

 

- **Genus incertae sedis**

 

***Acmaeodera burmitina* Cockerell, 1917**

*Acmaeodera burmitina* Cockerell, 1917: 323 [33].

Type material. Holotype, sex unknown, amber inclusion, No. PI In. 19,107 (BMNH).

Fossil deposit/age. Myanmar: Burmese amber; 99.6–93.5 Ma (Cretaceous).

Literature. Cockerell (1917: 14): remark [as Elateridae] [32]; Cockerell (1917: 323): original description [as Buprestidae] [33]; Fletcher (1920: 987): remark [186]; Štys (1969: 357): remark [as Buprestidae] [223]; Zherikhin (1978: 114): remark [187]; Spahr (1981: 14): catalogue [as Buprestidae] [49]; Keilbach (1982: 248): checklist [as Buprestidae] [133]; Poinar (1992: 136): remark [as Buprestidae] [188]; Bellamy (1995: 175): review [as Elateridae] [224]; Ross (1998: 13) remark [225]; Ross and York (2000: 12): catalogue [as Elateridae] [189]; Bellamy (2008: 41): catalogue [as Elateridae] [226]; Ding et al. (2014: Table ES1): checklist [as Buprestidae] [227]; Peris and Háva (2016: 496): remark [as Elateridae] [190]; Otto (2019: 2): remark [as Elateridae] [96].

Remark. Although this species was described in the buprestid genus *Acmaeodera* Eschscholtz, 1829, it represents a member of Elateridae [32,33,189,224,226].

## 4. Discussion

In this study, we summarized information on all described fossil species in Elateridae. Altogether, 261 fossil species classified in 99 genera and nine subfamilies are currently listed in this family. Nevertheless, our results show that our knowledge of click-beetle palaeodiversity varies widely with respect to systematic, spatial and temporal elements.

The highest diversity of fossil Elateridae lies in the only exclusively fossil subfamily Protagrypninae [12,51,53]. It contains 94 species in 31 genera classified in four tribes (Table A1), which is more than a third of the described species diversity of fossil Elateridae. Not surprisingly, the next most diverse subfamilies include Agrypninae (13 genera/35 spp.), Dendrometrinae (11/35) and Elaterinae (13/29), which are three most species-rich extant click-beetle subfamilies based on numbers of described species [1,228]. The remaining subfamilies are represented only by a few species each. It should be noted, however, that another 50 species are currently considered *incertae sedis*, without a subfamily assignment (Table A1).

Regarding the geographic origin of fossil click-beetle species, the highest diversity comes from Eurasian deposits. By far the richest locality is the famous Late Jurassic Karatau in Kazakhstan from which 100 species have been described in 29 genera [51,52,53]. Other, at least moderately rich localities, include the Jurassic Daohugou in Inner Mongolia of China (4 genera/4 spp.), the Lower Cretaceous Yixian Formation in northeastern China (7/10) and the Zaza Formation of Baissa in Siberia (2/6), and the Miocene Shanwang Formation in eastern China (2/6). In Europe, the highest diversity of click-beetles has been described from Eocene Baltic amber, which has been redeposited from its original stratigraphic positions mainly in marine sediments and fluvial deposits, and contains the most diverse assemblage of fossil insects to date, including 17 click-beetle species classified in 16 genera [47,48,229]. Other important localities include the world-famous Eocene Grube Messel Pit (2 genera/11 spp.) and the Miocene Öhningen within the Upper Freshwater-Molasse Formation in Germany (8/10). The world-famous Florissant in Colorado, USA (Eocene) is the richest deposit in North America, with 38 click-beetle species classified in 17 genera [28,29]. Five species in five genera are known from the Eocene Green River Formation in USA, and four species in three genera were described from Mexican “Chiapas” amber, which is Miocene in age [46,61]. Several fossil click-beetle lineages were reported also from South America. However, the genera *Babuskaya* Martins-Neto and Gallego, 2009, *Cardiosyne* Martins-Neto and Gallego, 2006, and *Gemelina* Martins-Neto and Gallego, 2006 from the Mesozoic Argentinian deposits [71,72] were recently transferred from Elateridae to Coleoptera *incertae sedis* [12], and two Paleocene species were described based on elytra only, and their placement in Elateridae is dubious [35]. The Australian click-beetle fossil fauna includes five species in four genera from Mesozoic deposits; however, at least four species highly probably do not belong to Elateridae (see, e.g., Muona et al. [99]). Nevertheless, considering the relatively unique and rich extant Australian click-beetle fauna [228], as well as the fact that at least some lineages were among the early splits in the elaterid phylogeny [5,11], a Mesozoic fossil record of that family in Australia would make sense. Indeed, Oberprieler et al. [98] reported a possible undescribed elaterid from the Jurassic Talbragar Fish Bed but this record needs further investigation. Thus far, there are no fossil Elateridae described from African deposits.

Our knowledge of the click-beetle fossil record should help us to better estimate the origin of the group and understand the evolutionary changes throughout its history. Based on the available data, it is possible that the Elateridae originated as early as the Triassic; however, this has to be confirmed by further research on the already described specimens and new material from Triassic deposits from around the globe. Currently, six described species are reported from the Triassic, most of them of highly doubtful family attribution, especially those from the Australian Blackstone Formation [39]. The highest diversity of fossil Elateridae is reported from the Jurassic (113 species/39 genera/five subfamilies), with the vast majority of lineages described from the rich Karatau deposit [51,52,53]. Only 24 species were described from Cretaceous localities, mainly from China and Russia, but we can expect that many more species will be discovered in Burmese amber in the near future. While 143 click-beetle species are recorded from the Mesozoic, only 118 described species are known from Cenozoic deposits. Most of them were from the Eocene Epoch, mainly due to the rich sources such as the North American Florissant Formation and European Baltic amber. It should be noted that all Mesozoic click-beetle species belong to fossil genera, i.e., those in which no extant representatives are included, with the apparently wrongly classified Burmese-amber species *Elater burmitinus* and “*Acmaeodera*” *burmitina* being exceptions [32,33]. On the other hand, slightly more than half of the elaterid genera known from the Paleogene (i.e., Paleocene, Eocene and Oligocene) also contain extant species, and among 12 genera known from the Neogene (Miocene) only two include exclusively fossil species.

Although our current study is the first comprehensive overview of described fossil species in Elateridae and is intended to serve as a solid basis for all future studies of the click-beetle fossil record, it is an annotated catalogue rather than a taxonomic revision. Therefore, it must be treated with caution and interpreted carefully. Our results clearly show that the major problem with the click-beetle fossil record lies in the highly questionable family placement of many lineages, incorrectly interpreted morphological characters for fossil higher click-beetle taxa, and dubious or sometimes clearly erroneous generic assignments of many species across the whole classification of Elateridae. Incorrect identification of Mesozoic specimens can obscure our understanding of the origin of Elateridae and greatly affect the accuracy of the dating of phylogenetic trees in various studies. Click-beetle fossils from the rich Karatau deposit were used as one of the calibration points for a recent Coleoptera phylogeny [230], and *Elaterophanes* was used as an important calibration point in a dated molecular phylogenetic analysis of Elateroidea [231] or even of the whole of Coleoptera [232]. Kusy et al. [10] showed that analyses using different datasets, applied models and calibrations often come to different age estimates for the major splits as well as for the origin of bioluminescence in Elateroidea.

Detailed investigation of the family placement of most Mesozoic taxa currently assigned to Elateridae is crucial for our understanding of the origin, early evolution and past diversity of the group. One of the problems is that some of the oldest fossils are known only from a single elytron or its fragment and therefore, their placement even to a (super) family remains questionable [31,37,39]. Another problem is the uncertain family placement of many compression fossils from the Mesozoic Asian deposits, mainly from China and Karatau in Kazakhstan. The systematic placement of some Chinese click-beetles was already questioned in several [86,233,234]. Recently, Muona et al. [99] studied the Mesozoic clicking Elateroidea from Chinese deposits and discussed the external characters for recognizing Eucnemidae from other clicking elateroids, especially Elateridae. They showed that only about a third, i.e., 12 of 27, described fossil click-beetle species from China can be attributed to Elateridae with more or less certainty. One species was transferred to Throscidae, six to Eucnemidae, three could be either Elateridae or Eucnemidae based on the available characters, and five could not be studied due to the unavailability of the type material. As correctly pointed out by the authors [99], this drastically changed our view of the Mesozoic clicking elateroid fauna in China.

Moreover, a similar or even more dramatic situation may occur after the putative click-beetle taxa from the extremely rich Karatau deposit are re-examined in detail based on the type material. Dolin [51,52,53] focused on that deposit and reported from there an exceptionally high diversity of Elateridae, with 107 described species in 31 genera from five subfamilies. Interestingly, the vast majority species from Karatau are classified in the only fossil elaterid subfamily, Protagrypninae (Table A1). It was proposed by Dolin [51] for the earlier defined tribe Protagrypnini, which he originally described based on two genera from the Dzhil Formation in Kyrgyzstan and placed it in Agrypninae [50], and two other tribes, Desmatini and Hypnomorphini, from Karatau. He defined the subfamily based on the presence of longitudinal furrows (sutures) definining a medial field on the prosternum, a transverse suture on the mesoventrite, and the additional division of the apex of the radial cell on the hind wing [51,53]. However, it was evident even from the descriptions and illustrations that many of the species originally assigned to the click-beetle subfamily Protagrypninae may in fact represent some other clicking elateroid lineages. Indeed, Chang et al. [77] removed several species of one genus from Hypnomorphini to Cerophytidae, and many other species with potentially eucnemid- or throscid-like characters should be re-examined (see Muona et al. [99] for extensive discussion on such characters, and remarks under various protagrypnine taxa in the overview of fossil species above). The diagnosis and monophyly of Protagrypninae are questionable, and especially problematic is the inclusion of Desmatini, which do not fully fit into the subfamily diagnosis as their representatives lack a clearly defined transverse suture on the mesoventrite [51,53]. They also have considerably broadened metacoxal plates, a character common in Eucnemidae ([86,234] but see [99]). Potential transfer of a number of taxa currently listed in Elateridae to Eucnemidae or Throscidae would make sense considering the earlier origin and longer evolutionary history of eucnemids, cerophytids and throscids [230,231,235]. Indeed, many recent studies confirm a high diversity of these families in the Mesozoic fossil record [96,99,218,233,234,236,237,238,239,240]. On the other hand, there might be some opposite cases. For example, Alekseev [74] described the monotypic genus *Cretopoena* from the Lower Cretaceous of Mongolia and attributed it to Eucnemidae; however, Li et al. [234] treated it as Elateriformia *incertae sedis* due to the lack of characters clearly separating Eucnemidae and Elateridae. Based on the general body shape and the structure of thorax this genus might indeed belong to Elateridae. Moreover, its thorax and elytra bear strong granulation which might be possible traces of scale-like setae typical for the Agrypnini. However, *Cretopoena* clearly differs from Agrypnini by its closed pronotosternal sutures and, therefore, its systematic position should be further investigated. Additionally, the suggested close relationship between Elateridae and recently discovered Mysteriomorphidae from the Cretaceous Burmese amber needs to be investigated using an analytical approach [241,242].

After the family placements of Mesozoic (and also younger) taxa are investigated in detail, it would be important to classify all fossil species to the proper genera and subfamilies. This will, however, be hampered not only by the lack of visible diagnostic characters on fossil specimens but also by the constantly changing definitions of the higher taxa and apparently unstable suprageneric classification of Elateridae [1,6,11,228]. The presence and character of the putative median plate-like structure on the prosternum laterally defined by furrows or sutures in Protagrypninae was discussed and questioned by Muona et al. [99] who concluded based on a study of fossil and extant specimens of clicking elateroids that it might be a place which is abruptly lower than the surrounding portions of the prosternum. The second important character, i.e., the transverse suture on the mesoventrite, which mainly defines Protagrypnini and Hypnomorphini, may in fact represent the line between the mesoventrite body and the depressions on the anterior edge of the mesoventrite and mesanepisternum, which are common in clicking elateroids and for which we use the terms “procoxal rests” [243] or “anterior articulating surface” [244]. The secondarily divided radial cell in the hind wing venation needs further investigation as this character is usually difficult to observe in compression fossils. Among the Cenozoic click-beetle fossils, those which are in the most urgent need of revision are species described by Scudder [19] and Wickham [27,28], especially those from the Green River and Florissant Formations [245], and species described by Heer from Öhningen [14], for which similarly inaccurate generic attributions were also reported in other beetle families [246,247]. Of special interest are the waste-basket genera such as *Elater* or *Ctenicera*. Further, maximum effort should be put into the study of the genera *incertae sedis* which include 50 species from various geological ages (Table A1), and also into description of the as-yet formally undescribed click-beetle fossils reported from various deposits [229,248,249], as they may provide further information on the diversity of the main click-beetle lineages.

Last but not least, Elateridae students should pay special attention to the study of fossils included in amber deposits. Although the research of beetles (and other taxa) from various ambers is nowadays very popular [92,188,189,250,251,252] and scientists were even able to describe within a short time span several new beetle families based on amber material [241,253,254,255,256,257], the diversity of click-beetles in fossilized plant resins has been highly understudied. A study of the Elateridae diversity in amber is of great importance due to the three-dimensional preservation of specimens which allows us to compare the fossil fauna with extant specimens in much greater detail than in the case of compression fossils [188,229]. With the use of modern techniques such as micro-CT, researchers are able to reconstruct the morphology of a particular beetle even when the imporant diagnostic characters are obscured by opaque bubbles or suboptimal body position [94,242,258]. Regarding the click-beetle diversity in amber, only a few formally undescribed Elateridae were reported from Cretaceous Lebanese and Oligocene/Miocene Dominican ambers [54,95,188,253]; personal observations of authors]. Becker [46] and Zaragoza Caballero [61] described four species from the Miocene Mexican “Chiapas” amber. Cretaceous Burmese amber contains a high number of Elateridae from various lineages [249,259]; personal observations of authors] but only two species were described by Cockerell [33] (although one originally in Bupestidae) and a single species by Otto [96]. Elateridae were among the most-represented beetle families in Eocene Baltic amber [30,48]; hence it is not surprising that most click-beetle species were described from that amber, mainly due to the work by Iablokoff-Khnzorian [47]. However, the vast majority of click-beetles known from all ambers remain undescribed.

## 5. Conclusions

An understanding of the origin, evolution, and past diversity of click-beetles is hampered by the lack of detailed knowledge on their fossil record. We summarized the current knowledge on all described fossil species in Elateridae, and assessed each species based on its description and available illustrations to conclude whether its position in Elateridae and its generic attribution can be considered reliable or not. Our results suggest that the Triassic records based largely on isolated elytra are mostly dubious and may belong to different beetle families, and numerous Jurassic and Cretaceous lineages currently listed in Elateridae might belong to Eucnemidae or Throscidae. The vast majority of the fossil click-beetle species are in urgent need of revision, and the *incertae sedis* genera should be investigated to correctly assign them to subfamilies and tribes. We can expect many more lineages to be discovered mainly from the more and more intensively studied amber inclusions, especially from Eocene Baltic amber and Cretaceous Burmese amber, which both include a relatively high proportion of elaterid fossils.

## Data Availability

Not applicable.

## Appendix A

**Table A1:** Overview of fossil Elateridae. Genera marked with an asterisk (*) also contain recent species. Geographic origin includes country and formation when available. Period/Epoch: T, Triassic; J, Jurassic; C, Cretaceous; P, Paleocene; E, Eocene; O, Oligocene; M, Miocene.

Subfamily, Tribe, Genus	Species	Geographic Origin	Age (Ma), Period/Epoch
**Agrypninae**			
**Agrypnini**			
*Adelocera* Latreille, 1829 *	*A. perantiqua* Cockerell and LeVeque, 1931	USA: Green River	50.3–46.2 (E)
*Ageratus* Dolin, 1980	*A. delicatus* Dolin, 1980	Kazakhstan: Karabastau	166.1–157.3 (J)
	*A*. p *onomarenkoi* Dolin, 1980	Kazakhstan: Karabastau	166.1–157.3 (J)
*Agrypnus* Eschscholtz, 1829 *	*A. exhumatus* (Wickham, 1916)	USA: Florissant	37.2–33.9 (E)
*Compsoderus* Dolin, 1980	*C. priscus* Dolin, 1980	Kazakhstan: Karabastau	166.1–157.3 (J)
*Lacon* Laporte, 1838 *	*L. granulatus* (Heer, 1847)	Germany: Upper Freshwater-Molasse	12.7–11.608 (M)
	*L. jungi* (Piton, 1940)	France: Menat	61.6–59.2 (P)
	*L. primordialis* Heer, 1847	Germany: Upper Freshwater-Molasse	12.7–11.608 (M)
*Litholacon* Dolin, 1980	*L. conicicollis* Dolin, 1980	Kazakhstan: Karabastau	166.1–157.3 (J)
	*L. derumpens* Dolin, 1980	Kazakhstan: Karabastau	166.1–157.3 (J)
	*L. exilis* Dolin, 1980	Kazakhstan: Karabastau	166.1–157.3 (J)
	*L. major* Dolin, 1980	Kazakhstan: Karabastau	166.1–157.3 (J)
	*L. panphilovi* Dolin, 1980	Kazakhstan: Karabastau	166.1–157.3 (J)
	*L. ohiri* Dolin, 1980	Kazakhstan: Karabastau	166.1–157.3 (J)
	*L. petrorsus* Dolin, 1980	Kazakhstan: Karabastau	166.1–157.3 (J)
*Macropunctum* Tröster, 1991	*M. angulosum* Tröster 1999	Germany: Messel	48.6–40.4 (E)
	*M. angustiscutellum* Tröster, 1994	Germany: Messel	48.6–40.4 (E)
	*M. densipunctum* Wappler, 2003	Germany: Eifel	48.6–40.4 (E)
	*M. eckfeldi* Tröster, 1992	Germany: Eifel	48.6–40.4 (E)
	*M. eocaenicum* (Meunier, 1921)	Germany: Messel	48.6–40.4 (E)
	*M. latiscutellum* Tröster	Germany: Messel	48.6–40.4 (E)
	*M. messelense* Tröster, 1991	Germany: Messel	48.6–40.4 (E)
	*M. meunieri* Tröster, 1991	Germany: Messel	48.6–40.4 (E)
	*M. minutum* (Meunier, 1921)	Germany: Messel	48.6–40.4 (E)
	*M. promptum* (Meunier, 1921)	Germany: Messel	48.6–40.4 (E)
	*M. rebugense* Tröster, 1994	Germany: Messel	48.6–40.4 (E)
	*M. rossi* Alekseev, 2019	United Kingdom: Bouldnor	38.0–33.9 (E)
	*M. senckenbergi* Tröster, 1994	Germany: Messel	48.6–40.4 (E)
*Plagioraphes* Iablokoff-Khnzorian, 1961	*P. fasciatus* Iablokoff-Khnzorian, 1961	Europe: Baltic amber	38.0–33.9 (E)
**Cryptocardiini**			
*Cryptocardius* Dolin, 1980	*C. mirabilis* Dolin, 1980	Kazakhstan: Karabastau	166.1–157.3 (J)
**Hemirhipini**			
*Alaus* Eschscholtz, 1829 *	*A. spectabilis* (Heer, 1865)	Germany: Upper Freshwater-Molasse	12.7–11.608 (M)
**Oophorini**			
*Monocrepidius* Eschscholtz, 1829 *	*M. dubiosus* Wickham, 1916	USA: Florissant	37.2–33.9 (E)
**Pseudomelanactini**			
*Lanelater* Arnett, 1952 *	*L. nicoleae* Wappler, 2003	Germany: Eifel	48.6–40.4 (E)
	*L. verae* Tröster, 1993	Germany: Messel	48.6–40.4 (E)
**Pyrophorini**			
*Eopyrophorus* Haupt, 1950	*E. mixtus* Haupt, 1950	Germany: Geiseltal	47.8–41.3 (E)
**Cardiophorinae**			
*Cardiophorus* Eschscholtz, 1829 *	*C. braunii* Heer, 1847	Germany: Upper Freshwater-Molasse	12.7–11.608 (M)
	*C. cockerelli* Wickham, 1916	USA: Florissant	37.2–33.9 (E)
	*C. deprivatus* Wickham, 1916	USA: Florissant	37.2–33.9 (E)
	*C. exhumatus* Cockerell, 1926	USA: Green River	50.3–46.2 (E)
	*C. florissantensis* Wickham, 1916	USA: Florissant	37.2–33.9 (E)
	*C. lithographus* Wickham, 1916	USA: Florissant	37.2–33.9 (E)
	*C. requiescens* Wickham, 1916	USA: Florissant	37.2–33.9 (E)
	*C. yatsenkokhmelevskyi* Iablokoff-Khnzorian, 1961	Europe: Baltic amber	38.0–33.9 (E)
*Horistonotus* Candèze, 1860 *	*H. coloradensis* Wickham, 1916	USA: Florissant	37.2–33.9 (E)
**Dendrometrinae**			
**Dendrometrini**			
*Athous* Eschscholtz, 1829 *			
Subg. *Athousiomorphus* Iablokoff-Khnzorian, 1961	*A*. (*A.*) *olgae* Iablokoff-Khnzorian, 1961	Europe: Baltic amber	38.0–33.9 (E)
Subg. *incertae sedis*	*A. contusus* Wickham, 1916	USA: Florissant	37.2–33.9 (E)
	*A. fractus* Wickham, 1916	USA: Florissant	37.2–33.9 (E)
	*A. holmgreni* (Heer, 1870)	Norway: Firkanten	66.0–59.2 (P)
	*A. lethalis* Wickham, 1916	USA: Florissant	37.2–33.9 (E)
*Limonius* Eschscholtz, 1829 *			
Subg. *Paralimonius* Iablokoff-Khnzorian, 1961	*L*.(*P.*) *barovskyi* Iablokoff-Khnzorian, 1961	Europe: Baltic amber	38.0–33.9 (E)
Subg. *incertae sedis*	*L. aboriginalis* Wickham, 1916	USA: Florissant	37.2–33.9 (E)
	*L. florissantensis* Wickham, 1916	USA: Florissant	37.2–33.9 (E)
	*L. impunctus* Scudder, 1895	Canada: Allenby	56.0–47.8 (E)
	*L. optabilis* Heer, 1847	Germany: Upper Freshwater-Molasse	12.7–11.608 (M)
	*L. praecursor* Wickham, 1916	USA: Florissant	37.2–33.9 (E)
	*L. shoshonis* Wickham, 1916	USA: Florissant	37.2–33.9 (E)
	*L. volans* Wickham, 1916	USA: Florissant	37.2–33.9 (E)
**Dimini**			
*Alaodima* Dolin, 1980	*A. grandis* Dolin, 1980	Kazakhstan: Karabastau	166.1–157.3 (J)
**Hypnoidini**			
*Ligmargus* Stibick, 1976 *	*L. terrestris* (Scudder, 1879)	Canada: Nicola river	56.0–47.8 (E)
**Oxynopterini**			
*Campsosternus* Latreille, 1834 *	*C. atavus* Deichmüller, 1881	Czech Republic: Kučlín	37.2–33.9 (E)
*Melanactes* LeConte, 1853 *	*M. cockerelli* Wickham, 1908	USA: Florissant	37.2–33.9 (E)
**Prosternini**			
*Ctenicera* Latreille, 1829 *	*C. emblemoelytra* (Zhang, 1989)	China: Shanwang	20.44–15.97 (M)
	*C. euprepes* (Zhang et al., 1994)	China: Shanwang	20.44–15.97 (M)
	*C. granulicollis* (Wickham, 1908)	USA: Florissant	37.2–33.9 (E)
	*C. primitiva* (Wickham, 1908)	USA: Florissant	37.2–33.9 (E)
	*C. prophetica* (Wickham, 1916)	USA: Florissant	37.2–33.9 (E)
	*C. restructa* (Wickham, 1916)	USA: Florissant	37.2–33.9 (E)
	*C. sincera* (Zhang et al., 1994)	China: Shanwang	20.44–15.97 (M)
	*C. submersa* (Wickham, 1916)	USA: Florissant	37.2–33.9 (E)
	*C. sutor* (Heer, 1847)	Germany: Upper Freshwater-Molasse	12.7–11.608 (M)
	*C. velata* (Scudder, 1876)	USA: Green River	50.3–46.2 (E)
*Eanus* LeConte, 1861 *	*E. exanimatus* (Wickham, 1916)	USA: Florissant	37.2–33.9 (E)
	*E. heeri* (Wickham, 1916)	USA: Florissant	37.2–33.9 (E)
	*E. laevissimus* (Wickham, 1916)	USA: Florissant	37.2–33.9 (E)
*Oxygonus* LeConte, 1863 *	*O. mortuus* Scudder, 1876	USA: Green River	50.3–46.2 (E)
	*O. primus* Wickham, 1916	USA: Florissant	37.2–33.9 (E)
**Selatosomini**			
*Selatosomus* Stephens, 1830 *	*S. miegi* Theobald, 1937	Germany: Middle Member	33.9–28.4 (O)
**Semiotini**			
*Semiotus* Eschscholtz 1829 *	*S. ehrenswaerdi* (Heer, 1870)	Norway: Firkanten	66.0–59.2 (P)
	*S. menatensis* Piton, 1940	France: Menat	61.6–59.2 (P)
**Elaterinae**			
**Agriotini**			
*Agriotes* Eschscholtz, 1829 *	*A. comminutus* Wickham, 1916	USA: Florissant	37.2–33.9 (E)
	*A. nearcticus* Wickham, 1916	USA: Florissant	37.2–33.9 (E)
	*A. succiniferus* Becker, 1963	Mexico: Chiapas amber	23.03–15.97 (M)
**Ampedini**			
*Ampedus* Dejean, 1833 *			
Subg. *Octamenogonoides* Iablokoff-Khnzorian, 1961	*A. (O.) gebleri* Iablokoff-Khnzorian, 1961	Europe: Baltic amber	38.0–33.9 (E)
Subg. *Ampedus* Dejean, 1833 *	*A. seyfriedii* Heer, 1847	Germany: Upper Freshwater-Molasse	12.7–11.608 (M)
*Ischnodes* Germar, 1844 *	*I. gracilis* Heer, 1847	Germany: Upper Freshwater-Molasse	12.7–11.608 (M)
**Elaterini**			
*Diaraphes* Iablokoff-Khnzorian, 1961	*D. kozhantshikovi* Iablokoff-Khnzorian, 1961	Europe: Baltic amber	38.0–33.9 (E)
*Elater* Linnaeus, 1758 *	*E. asmodeus* Zhang, 1989	China: Shanwang	20.44–15.97 (M)
	*E. berryi* Wickham, 1929	USA: Cockfield	41.3–38.0 (E)
	*E. burmitinus* Cockerell, 1917	Myanmar: Burmese amber	99.6–93.5 (C)
	*E. canabinus* Zhang, 1989	China: Shanwang	20.44–15.97 (M)
	*E. florissantensis* Wickham, 1916	USA: Florissant	37.2–33.9 (E)
	*E. mitrus* Zhang, 1989	China: Shanwang	20.44–15.97 (M)
	*E. naumanni* Giebel, 1856	Europe: Baltic amber	38.0–33.9 (E)
	*E. rohweri* Wickham, 1916	USA: Florissant	37.2–33.9 (E)
	*E. scudderi* Wickham, 1916	USA: Florissant	37.2–33.9 (E)
	*E. wisniowskii* Lomnicki, 1902	Ukraine: Bashkev	13.65–12.7 (M)
*Elatron* Iablokoff-Khnzorian, 1961	*E. semenovi* Iablokoff-Khnzorian, 1961	Europe: Baltic amber	38.0–33.9 (E)
*Holopleurus* Iablokoff-Khnzorian, 1961	*H. succineus* Iablokoff-Khnzorian, 1961	Europe: Baltic amber	38.0–33.9 (E)
*Orthoraphes* Iablokoff-Khnzorian, 1961	*O. reichardti* Iablokoff-Khnzorian, 1961	Europe: Baltic amber	38.0–33.9 (E)
**Megapenthini**			
*Abelater* Fleutiaux, 1947 *	*A. succineus* Schimmel, 2005	Europe: Baltic amber	38.0–33.9 (E)
*Megapenthes* Kiesenwetter, 1858 *	*M. groehni* Schimmel, 2005	Europe: Baltic amber	38.0–33.9 (E)
	*M. primaevus* Wickham, 1916	USA: Florissant	37.2–33.9 (E)
	*M. voigti* Schimmel, 2005	Europe: Baltic amber	38.0–33.9 (E)
**Physorhinini**			
*Anchastus* LeConte, 1853 *	*A. diluvialis* Wickham, 1916	USA: Florissant	37.2–33.9 (E)
	*A. eruptus* Wickham, 1916	USA: Florissant	37.2–33.9 (E)
**Synaptini**			
*Glyphonyx* Candèze, 1863 *	*G. chiapasensis* Zaragoza Caballero, 1990	Mexico: Chiapas amber	23.03–15.97 (M)
	*G. punctatus* Becker, 1963	Mexico: Chiapas amber	23.03–15.97 (M)
**Elaterinae** *incertae sedis*			
*Crioraphes* Iablokoff-Khnzorian, 1961	*C. rohdendorfi* Iablokoff-Khnzorian, 1961	Europe: Baltic amber	38.0–33.9 (E)
**Lissominae**			
**Lissomini**			
*Lissomus* Dalman, 1824 *	*L. taxodii* (Heer, 1870)	Norway: Firkanten	66.0–59.2 (P)
**Protelaterini**			
*Baltelater* Kundrata et al., 2020	*B. bipectinatus* Kundrata et al., 2020	Europe: Baltic amber	38.0–33.9 (E)
**Negastriinae**			
*Ganestrius* Dolin, 1976	*G. elongatus* Dolin, 1976	Kazakhstan: Karabastau	166.1–157.3 (J)
	*G. stibicki* Dolin, 1976	Kazakhstan: Karabastau	166.1–157.3 (J)
*Paradonus* Stibick, 1971 *	*Paradonus exterminatus* (Wickham, 1916)	USA: Florissant	37.2–33.9 (E)
	*Paradonus hesperus* (Wickham, 1916)	USA: Florissant	37.2–33.9 (E)
*Protoquasimus* Dolin, 1976	*P. brevicollis* Dolin, 1976	Kazakhstan: Karabastau	166.1–157.3 (J)
**Omalisinae**			
*Jantarokrama* Kirejtshuk and Kovalev, 2015	*J. utilis* Kirejtshuk and Kovalev, 2015	Europe: Baltic amber	38.0–33.9 (E)
**Pityobiinae**			
*Cretopityobius* Otto, 2019	*C. pankowskiorum* Otto, 2019	Myanmar: Burmese amber	99.6–93.5 (C)
**Protagrypninae**			
**Desmatini**			
*Desmatinus* Chang et al., 2010	*D. cognatus* Chang et al., 2010	China: Yixian	125.45–122.46 (C)
*Desmatus* Dolin, 1975	*D. affinis* Dolin, 1975	Kazakhstan: Karabastau	166.1–157.3 (J)
	*D. beckeri* Dolin, 1975	Kazakhstan: Karabastau	166.1–157.3 (J)
	*D. lapidarius* Dolin, 1975	Kazakhstan: Karabastau	166.1–157.3 (J)
	*D. ponomarenkoi* (Chang et al., 2009)	China: Jiulongshan	166.1–157.3 (J)
	*D. protensus* Dolin, 1980	Kazakhstan: Karabastau	166.1–157.3 (J)
*Plesiorhaphes* Dolin, 1980	*P. scaber* Dolin, 1980	Kazakhstan: Karabastau	166.1–157.3 (J)
**Hypnomorphini**			
*Abrotus* Dolin, 1980	*A. reconditus* Dolin, 1980	Kazakhstan: Karabastau	166.1–157.3 (J)
	*A. sepultus* Dolin, 1980	Kazakhstan: Karabastau	166.1–157.3 (J)
*Adiagnostus* Dolin, 1980	*A. ambiguus* Dolin, 1980	Kazakhstan: Karabastau	166.1–157.3 (J)
	*A. cardiophorinus* Dolin, 1980	Kazakhstan: Karabastau	166.1–157.3 (J)
	*A. minutulus* Dolin, 1980	Kazakhstan: Karabastau	166.1–157.3 (J)
*Codemus* Dolin, 1980	*C. alatus* Dolin, 1980	Kazakhstan: Karabastau	166.1–157.3 (J)
	*C. carinatus* Dolin, 1980	Kazakhstan: Karabastau	166.1–157.3 (J)
	*C. jejunus* Dolin, 1980	Kazakhstan: Karabastau	166.1–157.3 (J)
	*C. martynovi* Dolin, 1980	Kazakhstan: Karabastau	166.1–157.3 (J)
	*C. micros* Dolin, 1980	Kazakhstan: Karabastau	166.1–157.3 (J)
	*C. quadricolis* Dolin, 1980	Kazakhstan: Karabastau	166.1–157.3 (J)
	*C. sharovi* Dolin, 1980	Kazakhstan: Karabastau	166.1–157.3 (J)
	*C. synaptoides* Dolin, 1980	Kazakhstan: Karabastau	166.1–157.3 (J)
	*C. teres* Dolin, 1980	Kazakhstan: Karabastau	166.1–157.3 (J)
	*C. zherichini* Dolin, 1980	Kazakhstan: Karabastau	166.1–157.3 (J)
*Dolinelater* Huber et al., 2017	*D. asperatus* (Dolin, 1980)	Kazakhstan: Karabastau	166.1–157.3 (J)
	*D. singularis* (Dolin, 1980)	Kazakhstan: Karabastau	166.1–157.3 (J)
*Elaterophanes* Handlirsch, 1906	*E. acutus* Cockerell, 1916	United Kingdom: Wainlode Cliff	208.5–201.3 (T)
	*E. regius* Whalley, 1985	United Kingdom: Charmouth Mudstone	196.5–189.6 (J)
	*E. vetustus* (Brodie, 1845)	United Kingdom: Lilstock	208.5–201.3 (T)
*Graciolacon* Dolin, 1980	*G. aeternus* Dolin, 1980	Kazakhstan: Karabastau	166.1–157.3 (J)
*Hypnomorphoides* Dolin, 1980	*H. angularis* Dolin, 1980	Kazakhstan: Karabastau	166.1–157.3 (J)
	*H. catachtonius* Dolin, 1980	Kazakhstan: Karabastau	166.1–157.3 (J)
	*H. latus* Dolin, 1980	Kazakhstan: Karabastau	166.1–157.3 (J)
	*H. procerulus* Dolin, 1980	Kazakhstan: Karabastau	166.1–157.3 (J)
*Hypnomorphus* Dolin, 1975	*H. aemulus* Dolin, 1975	Kazakhstan: Karabastau	166.1–157.3 (J)
	*H. angulosus* Dolin, 1980	Kazakhstan: Karabastau	166.1–157.3 (J)
	*H. carpolithus* Dolin, 1975	Kazakhstan: Karabastau	166.1–157.3 (J)
	*H. confusus* Dolin, 1975	Kazakhstan: Karabastau	166.1–157.3 (J)
	*H. curtus* Dolin, 1980	Kazakhstan: Karabastau	166.1–157.3 (J)
	*H. distinctus* Dolin, 1975	Kazakhstan: Karabastau	164.7–155.7 (J)
	*H. dubius* Dolin, 1975	Kazakhstan: Karabastau	166.1–157.3 (J)
	*H. gigas* Dolin, 1980	Kazakhstan: Karabastau	166.1–157.3 (J)
	*H. imperspicuus* Dolin, 1975	Kazakhstan: Karabastau	166.1–157.3 (J)
	*H. induratus* Dolin, 1975	Kazakhstan: Karabastau	166.1–157.3 (J)
	*H. inventus* Dolin, 1975	Kazakhstan: Karabastau	166.1–157.3 (J)
	*H. minutus* Dolin, 1975	Kazakhstan: Karabastau	166.1–157.3 (J)
	*H. rasnitzyni* Dolin, 1980	Kazakhstan: Karabastau	166.1–157.3 (J)
	*H. rohdendorfi* Dolin, 1975	Kazakhstan: Karabastau	166.1–157.3 (J)
*Lapidiconides* Dolin, 1980	*L. brevis* Dolin, 1980	Kazakhstan: Karabastau	166.1–157.3 (J)
	*L. excellens* Dolin, 1980	Kazakhstan: Karabastau	166.1–157.3 (J)
	*L. innatus* Dolin, 1980	Kazakhstan: Karabastau	166.1–157.3 (J)
*Lapidostenus* Dolin, 1980	*L. infossus* Dolin, 1980	Kazakhstan: Karabastau	166.1–157.3 (J)
	*L. insignis* Dolin, 1980	Kazakhstan: Karabastau	166.1–157.3 (J)
	*L. longicornis* Dolin, 1980	Kazakhstan: Karabastau	166.1–157.3 (J)
	*L. scutellaris* Dolin, 1980	Kazakhstan: Karabastau	166.1–157.3 (J)
	*L. tarbinskyi* Dolin, 1980	Kazakhstan: Karabastau	166.1–157.3 (J)
*Lithoptychus* Dolin, 1980	*L. carinatissimus* Dolin, 1980	Kazakhstan: Karabastau	166.1–157.3 (J)
	*L. handlirschi* Dolin, 1980	Kazakhstan: Karabastau	166.1–157.3 (J)
	*L. incertus* Dolin, 1980	Kazakhstan: Karabastau	166.1–157.3 (J)
	*L. minutus* Dolin, 1980	Kazakhstan: Karabastau	166.1–157.3 (J)
*Lithosomus* Dolin, 1980	*L. erosus* Dolin, 1980	Kazakhstan: Karabastau	166.1–157.3 (J)
	*L. longicollis* Dolin, 1980	Kazakhstan: Karabastau	166.1–157.3 (J)
*Necrocoelus* Dolin, 1980	*N. aselloides* Dolin, 1980	Kazakhstan: Karabastau	166.1–157.3 (J)
*Negastrioides* Dolin, 1980	*N. globicollis* Dolin, 1980	Kazakhstan: Karabastau	166.1–157.3 (J)
	*N. tenuicornis* Dolin, 1980	Kazakhstan: Karabastau	166.1–157.3 (J)
	*N. tenuis* Dolin, 1980	Kazakhstan: Karabastau	166.1–157.3 (J)
	*N. tscherepanovi* Dolin, 1980	Kazakhstan: Karabastau	166.1–157.3 (J)
*Parahypnomorphus* Dolin, 1980	*P. jurassicus* Dolin, 1980	Kazakhstan: Karabastau	166.1–157.3 (J)
	*P. longicornis* Dolin, 1980	Kazakhstan: Karabastau	166.1–157.3 (J)
	*P. similis* Dolin, 1980	Kazakhstan: Karabastau	166.1–157.3 (J)
*Platyelater* Dolin, 1980	*P. figeratus* Dolin, 1980	Kazakhstan: Karabastau	166.1–157.3 (J)
	*P. quiescentus* Dolin, 1980	Kazakhstan: Karabastau	166.1–157.3 (J)
	*P. reflexicollis* Dolin, 1980	Kazakhstan: Karabastau	166.1–157.3 (J)
	*P. sukatschevae* Dolin, 1980	Kazakhstan: Karabastau	166.1–157.3 (J)
**Pollostelaterini**			
*Pollostelater* Alekseev, 2011	*P. baissensis* Alekseev, 2011	Russia: Zaza	125.0–113.0 (C)
**Protagrypnini**			
*Acheonus* Dolin, 1980	*A. abbreviatus* Dolin, 1980	Kazakhstan: Karabastau	166.1–157.3 (J)
	*A. gracilis* Dolin, 1980	Kazakhstan: Karabastau	166.1–157.3 (J)
	*A. minutissimus* Dolin, 1980	Kazakhstan: Karabastau	166.1–157.3 (J)
*Clavelater* Dong and Huang, 2011	*C. ningchengensis* Dong and Huang, 2011	China: Jiulongshan	166.1–157.3 (J)
*Koreagrypnus* Sohn and Nam, 2019	*K. jinju* Sohn and Nam, 2019	South Korea: Jinju	113.0–100.5 (C)
*Lithocoelus* Dolin, 1975	*L. detrusus* Dolin, 1975	Kazakhstan: Karabastau	166.1–157.3 (J)
	*L. karatavicus* Dolin, 1975	Kazakhstan: Karabastau	166.1–157.3 (J)
*Lithomerus* Dolin, 1980	*L. brachycollis* Dolin, 1980	Kazakhstan: Karabastau	166.1–157.3 (J)
	*L. brevicollis* Dolin, 1980	Kazakhstan: Karabastau	166.1–157.3 (J)
	*L. buyssoni* Dolin and Nel, 2002	China: Yixian	125.45–122.46 (C)
	*L. cockerelli* Dolin, 1980	Kazakhstan: Karabastau	166.1–157.3 (J)
	*L. contiguus* Dolin, 1980	Kazakhstan: Karabastau	166.1–157.3 (J)
	*L. longulus* Dolin, 1980	Kazakhstan: Karabastau	166.1–157.3 (J)
	*L. wunda* Martin, 2010	Australia: Cattamarra Coal Measures	182.7–174.1 (J)
*Megalithomerus* Sohn and Nam, 2019	*M. magohalmii* Sohn and Nam, 2019	South Korea: Jinju	113.0–100.5 (C)
*Micragrypnites* Dolin, 1973	*M. issykiensis* Dolin, 1973	Kyrgyzstan: Dzhil	201.3–190.8 (J)
*Paragrypnites* Dolin, 1980	*P. jagemanni* Dolin, 1980	Kazakhstan: Karabastau	166.1–157.3 (J)
*Paraprotagrypnus* Chang et al., 2009	*P. superbus* Chang et al., 2009	China: Jiulongshan	166.1–157.3 (J)
*Protagrypnus* Dolin, 1973	*P. exoletus* Dolin, 1973	Kyrgyzstan: Dzhil	201.3–190.8 (J)
	*P. robustus* Chang et al., 2009	China: Jiulongshan	166.1–157.3 (J)
*Sinolithomerus* Dong and Huang, 2009	*S. dolini* Dong and Huang, 2009	China: Haifanggou	166.1–157.3 (J)
**Elateridae** *incertae sedis*			
*Adocetus* Scudder, 1900	*A. buprestoides* Scudder, 1900	USA: Green River	55.8–50.3 (E)
*Artinama* Lin, 1986	*A. qinghuoensis* Lin, 1986	China: Zaoshang	199.3–190.8 (J)
*Bilineariselater* Chang and Ren, 2008	*B. foveatus* Chang and Ren, 2008	China: Yixian	125.45–122.46 (C)
*Cretoelaterium* Alekseev, 2008	*C. kazanovense* Alekseev, 2008	Russia: Mirsanovo	129.4–125.0 (C)
*Cryptagriotes* Wickham, 1916	*C. minusculus* Wickham, 1916	USA: Florissant	37.2–33.9 (E)
*Cryptocoelus* Dolin and Nel, 2002	*C. baissensis* Alekseev, 2011	Russia: Zaza	125.0–113.0 (C)
	*C. buffoni* Dolin and Nel, 2002	China: Yixian	125.45–122.46 (C)
	*C. dolini* Alekseev, 2011	Russia: Zaza	125.0–113.0 (C)
	*C. gianteus* Chang et al., 2007	China: Yixian	125.45–122.46 (C)
	*C. lukashevichae* Alekseev, 2011	Russia: Zaza	125.0–113.0 (C)
	*C. major* Dolin and Nel, 2002	China: Yixian	125.45–122.46 (C)
	*C. shcherbakovi* Alekseev, 2011	Russia: Zaza	125.0–113.0 (C)
	*C. sinitshenkovae* Alekseev, 2011	Russia: Zaza	125.0–113.0 (C)
*Curtelater* Chang and Ren, 2008	*C. wui* Chang and Ren, 2008	China: Yixian	125.45–122.46 (C)
*Elateridium* Tillyard, 1918	*E. subulatum* (Dunstan, 1923)	Australia: Blackstone	228.0–208.5 (T)
	*E. transversum* (Dunstan, 1923)	Australia: Blackstone	228.0–208.5 (T)
	*E. wianamattense* (Tillyard, 1916)	Australia: Ashfield Shales	247.2–242.0 (T)
*Elaterites* Heer, 1847	*E. amissus* Heer, 1847	Switzerland: Greith coal mine	28.4–23.03 (O)
	*E. bruchi* Cockerell, 1926	Argentina: Margas Verdes	66.0–56.0 (P)
	*E. dicrepidioides* Deichmüller, 1881	Czech Republic: Kučlín	37.2–33.9 (E)
	*E. laconoides* Cockerell, 1920	United Kingdom: Poole	47.8–41.3 (E)
	*E. lavateri* Heer, 1847	Germany: Upper Freshwater-Molasse	12.7–11.608 (M)
	*E. longus* Haupt, 1956	Germany: Geiseltal	47.8–41.3 (E)
	*E. microstictus* Cockerell, 1926	Argentina: Margas Verdes	66.0–56.0 (P)
	*E. murchisoni* (Giebel, 1856)	United Kingdom: Poole	47.8–41.3 (E)
	*E. obsoletus* Heer, 1847	Germany: Upper Freshwater-Molasse	12.7–11.608 (M)
	*E. palaeophilus* Cockerell, 1920	United Kingdom: Peckham	56.0–47.8 (E)
	*E. perditulus* Cockerell, 1920	United Kingdom: Poole	47.8–41.3 (E)
	*E. sculptilis* Cockerell, 1920	United Kingdom: Poole	47.8–41.3 (E)
*Elaterium* Westwood, 1854	*E. bipunctatum* Dunstan, 1923	Australia: Blackstone	228.0–208.5 (T)
	*E. pronaeus* Westwood, 1854	United Kingdom: Lulworth	145.0–140.2 (C)
*Gripecolous* Lin, 1986	*G. enallus* Lin, 1986	China: Shiti	170.3–168.3 (J)
*Ludiophanes* Wickham, 1916	*L. haydeni* Wickham, 1916	USA: Florissant	37.2–33.9 (E)
*Mercata* Lin, 1986	*M. festira* Lin, 1986	China: Shiti	170.3–168.3 (J)
*Mionelater* Becker, 1963	*M. planatus* Becker, 1963	Mexico: Chiapas amber	23.03–15.97 (M)
*Ovivagina* Zhang, 1997	*O. longa* Zhang, 1997	China: Badaowan	201.3–190.8 (J)
*Paralithomerus* Chang et al., 2008	*P. exquisitus* Chang et al., 2008	China: Yixian	125.45–122.46 (C)
	*P. parallelus* Chang et al., 2008	China: Yixian	125.45–122.46 (C)
*Protocardiophorus* Dolin, 1976	*P. ancestralis* Dolin, 1976	Kazakhstan: Karabastau	166.1–157.3 (J)
	*P. jurassicus* Dolin, 1980	Kazakhstan: Karabastau	166.1–157.3 (J)
*Pseudocardiophorites* Dolin, 1976	*P. angustatus* Dolin, 1980	Kazakhstan: Karabastau	166.1–157.3 (J)
	*P. fragilis* Dolin, 1976	Kazakhstan: Karabastau	166.1–157.3 (J)
	*P. hayeki* Dolin, 1976	Kazakhstan: Karabastau	166.1–157.3 (J)
	*P. infractus* Dolin, 1976	Kazakhstan: Karabastau	166.1–157.3 (J)
	*P. quadricollis* Dolin, 1976	Kazakhstan: Karabastau	166.1–157.3 (J)
*Silicernius* Heyden, 1859	*S. spectabilis* Heyden, 1859	Germany: Rott	28.4–23.03 (O)
*Sinoelaterium* Ping, 1928	*S. melanocolor* Ping, 1928	China: Yixian	125.45–122.46 (C)
*Tetraraphes* Iablokoff-Khnzorian, 1961	*T. ebersini* Iablokoff-Khnzorian, 1961	Europe: Baltic amber	38.0–33.9 (E)
*Turonelater* Alekseev, 2011	*T. giganteus* Alekseev, 2011	Kazakhstan: Kzyl-Zhar	93.9–89.8 (C)
Genus *incertae sedis*	*Acmaeodera burmitina* Cockerell, 1917	Myanmar: Burmese amber	99.6–93.5 (C)
